# Soft QPCs: Biscationic Quaternary Phosphonium Compounds as Soft Antimicrobial Agents

**DOI:** 10.1021/acsinfecdis.2c00624

**Published:** 2023-03-16

**Authors:** Samantha R. Brayton, Zachary E. A. Toles, Christian A. Sanchez, Marina E. Michaud, Laura M. Thierer, Taylor M. Keller, Caitlin J. Risener, Cassandra L. Quave, William M. Wuest, Kevin P. C. Minbiole

**Affiliations:** † Department of Chemistry, Villanova University, Villanova, Pennsylvania 19085, United States; ‡ Department of Chemistry, Emory University, Atlanta, Georgia 30322, United States; § Department of Chemistry Crystallography Facility, University of Pennsylvania, Philadelphia, Pennsylvania 19104, United States; ∥ Molecular and Systems Pharmacology Program, Emory University, Atlanta, Georgia 30322, United States; ⊥ Department of Dermatology, Emory University School of Medicine, Emory University, Atlanta, Georgia 30322, United States

**Keywords:** amphiphiles, disinfectants, phosphorus, QPCs, soft disinfectants

## Abstract

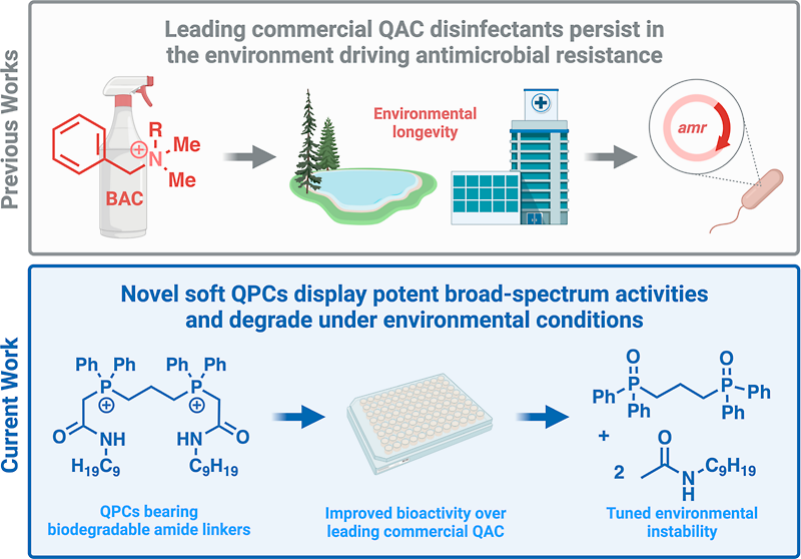

Quaternary ammonium compounds (QACs) serve as a first line of defense against infectious pathogens. As resistance to QACs emerges in the environment, the development of next-generation disinfectants is of utmost priority for human health. Balancing antibacterial potency with environmental considerations is required to effectively counter the development of bacterial resistance. To address this challenge, a series of 14 novel biscationic quaternary phosphonium compounds (bisQPCs) have been prepared as amphiphilic disinfectants through straightforward, high-yielding alkylation reactions. These compounds feature decomposable or “soft” amide moieties in their side chains, anticipated to promote decomposition under environmental conditions. Strong bioactivity against a panel of seven bacterial pathogens was observed, highlighted by single-digit micromolar activity for compounds P6P-12A,12A and P3P-12A,12A. Hydrolysis experiments in pure water and in buffers of varying pH revealed surprising decomposition of the soft QPCs under basic conditions at the phosphonium center, leading to inactive phosphine oxide products; QPC stability (>24 h) was maintained in neutral solutions. The results of this work unveil soft QPCs as a potent and environmentally conscious new class of bisQPC disinfectants.

The relentless emergence of SARS-CoV-2 variants and the looming threat of future pandemics highlight the urgent need to develop potent disinfectants.^1^ To address the spread and transmission of SARS-CoV-2, the Center for Disease Control and Prevention and the Environmental Protection Agency have maintained a registry^2^ of disinfectants to combat current and future outbreaks.^3^ Quaternary ammonium compounds (QACs), which represent the largest share of active agents in the list, have served as effective antiseptics and disinfectants for many decades. QACs act by electrostatically adhering to bacterial membranes via their cationic head and subsequently disrupting the membrane with the insertion of their lipophilic tail. This class of disinfectants has enjoyed tremendous success and usage in a wide number of antimicrobial applications. However, bacteria are increasingly displaying resistance to known QACs, taking advantage of a multitude of resistance mechanisms, including upregulation of antimicrobial efflux pumps, alterations to the bacterial membrane, and enzymatic degradation of QACs.^4,5^ The paucity of structural diversity among the select number of QACs in commercial use has further driven QAC cross-resistance via these mechanisms, attenuating the efficacy of many disinfectants. Moreover, the presence of persistent QACs in the environment at subinhibitory concentrations has been demonstrated to drive the spread of antibiotic resistance in addition to QAC resistance.^6^

Concurrent with this rise in bacterial resistance, robust QACs are being shown to negatively impact the environment. During the COVID-19 pandemic, the use of antimicrobial materials rose to unprecedented levels, and the usage remains elevated.^7^ It was reported that 75% of QACs are released into wastewater treatment plants (WWTPs), and the remaining is directly released into the environment.^8^ In most cases, WWTPs can remove the bulk of QACs through absorption into activated sludge. However, studies show that residual QAC concentrations of 20–300 μg/L have been found in surface water even after treatment.^9^ QACs are still found in aquatic environments, especially at higher concentrations when downstream from municipal/industrial wastewater treatment plants and hospitals.^10^ The most commonplace QAC, benzalkonium chloride (BAC), possesses an environmental half-life of 9 months due to its chemical stability^11^ and slow biodegradation.^12,13^ Long-term environmental exposure to QACs has shown growth inhibition and lethal effects in most aquatic organisms. In addition, increased antimicrobial resistance has emerged as these compounds persist in the environment.^14^ The need for novel antimicrobial agents that balance strong protection against emerging pathogens, while presenting limited stability during environmental exposure, is of paramount importance.

Over the past decade, our collaborative efforts have led to the preparation and biological assessment of over 700 novel QACs.^15,16^ Previously, our groups investigated the assembly of novel hydrolyzable amphiphiles aiming to develop a library of antimicrobial agents with tuned instability to mitigate their environmental persistence.^17^ Exploiting varying multicationic QAC architectures, we reported in 2017 the synthesis of 40 QACs bearing either ester or amide linkers with encouraging antimicrobial and stability data (Figure 1). While the ester-containing QACs were generally short-lived in our hands, the stability of the amide-containing QACs displayed a direct correlation with the overall pH of the sample. Under acidic conditions, the amides immediately decomposed, but the stability of over 24 h was apparent in deionized water and buffered solutions of pH ≥ 7. Importantly, these amide-based QACs displayed potent antimicrobial activity, with minimum inhibitory concentration (MIC) values at single-digit micromolar values. Our work added to a growing literature of QACs with designed instability (thus “soft QACs”), joining the efforts of Bodor, who coined the term “soft antimicrobials,”^18^ as well as Ahlstrom,^19^ Haldar,^20,21^ and others (Figure 1A).

**Figure 1:**
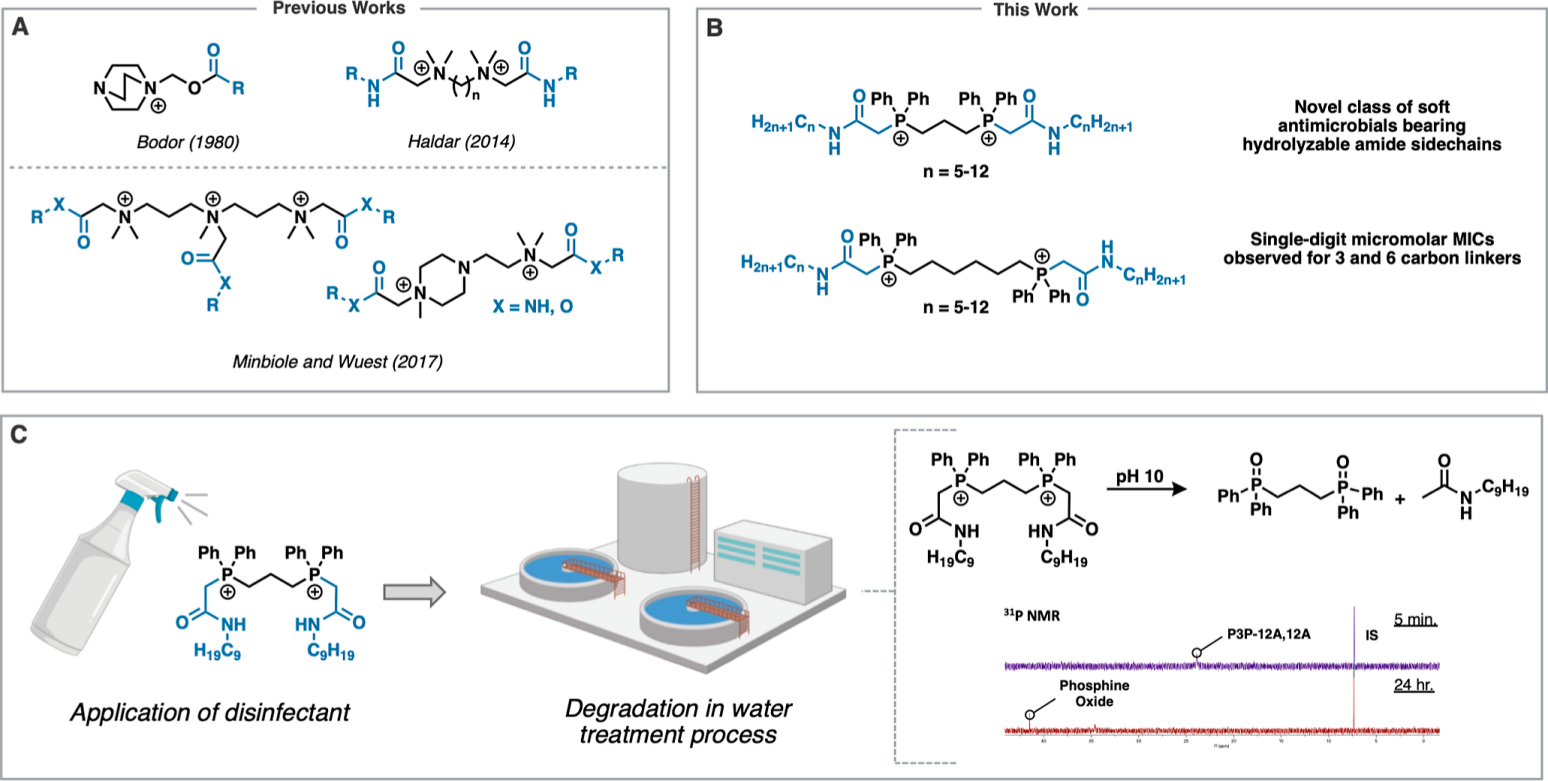
(A) Previous ester- and amide-containing antimicrobial QACs.^15−19^ (B) Present work of novel amide-containing soft QPCs. (C) Proposed water treatment plant breakdown strategy of soft QPCs. Images prepared with BioRender.

More recently, our efforts have turned to the investigation of quaternary phosphonium compounds (QPCs) as an alternative set of antimicrobial agents to QACs with diverse architectures and potent broad-spectrum activities. Initial results have shown that biscationic QPCs (bisQPCs) exhibit high potency against both Gram-positive and Gram-negative pathogenic bacterial strains with the ability to evade QAC resistance mechanisms while still allowing for straightforward construction.^22^ Due to the lack of decomposition studies on such a disinfectant class, we turned our focus to the construction of a library of soft antimicrobial QPCs (soft QPCs, Figure 1B, C). Accordingly, we prepared a series of bisQPCs that bear amide linkages in the side chains, anticipating that amide hydrolysis would lead to the formation of nonamphiphilic residues, and evaluated these compounds for bioactivity, toxicity, and stability in varying buffer solutions, facilitating comparison to the analogous soft QACs previously reported.^17^

## Results and Discussion

**Scheme 1:**
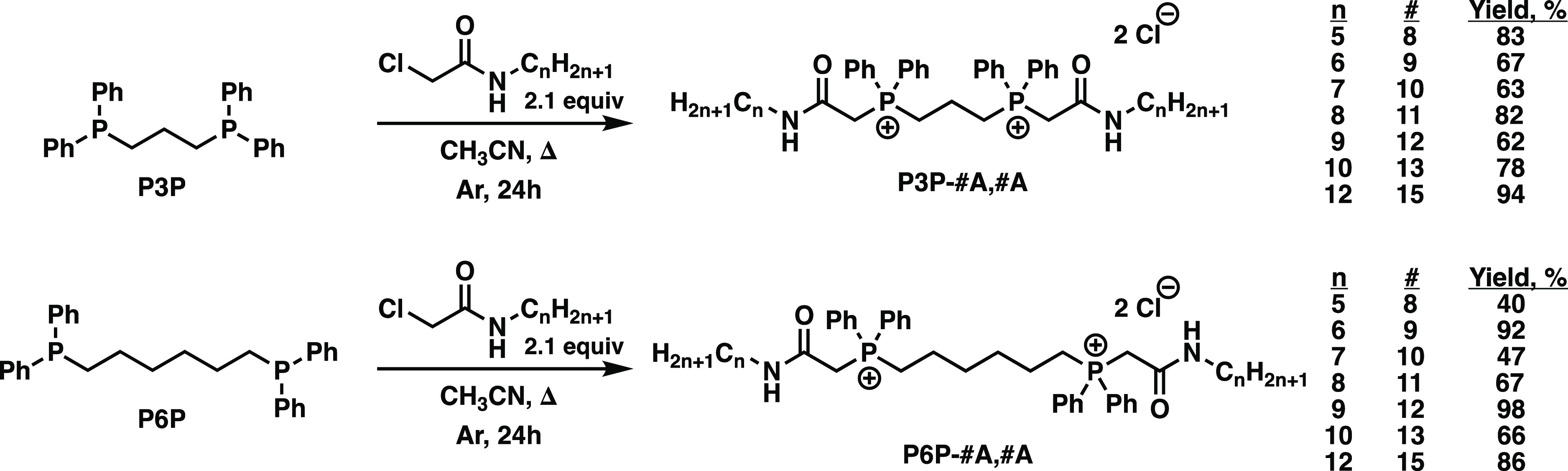
Synthesis of Soft QPC Structures

We successfully synthesized 14 amide-based soft QPCs, varying the distance between the phosphonium cations and alkyl chain lengths, inspired by literature precedents.^13,14^ In the first step of the synthesis, the amide-bearing lipophilic group was constructed by exposing long-chained amines to chloroacetyl chloride.^23^ The resulting *N*-alkyl-2-chloroacetamide building blocks were then reacted with two readily available bisphosphine nucleophiles: 1,3-bis(diphenylphosphino)propane (dppp) and 1,6-bis(diphenylphosphino)hexane (dpph). The bisalkylation reactions were successful under standard S_N_2 conditions (acetonitrile, reflux, 24 h, argon atmosphere), affording 14 QPCs in good yields (40–98%; Scheme 1). Purification of the crude products was accomplished via trituration with cyclohexane. We continued a naming system from our previous works using a PmP format, where m reflects the number of carbons linking the two phosphorus atoms (*m* = 3, 6; P3P or P6P with propyl or hexyl connections, respectively). The length of the side chain is identified by the total number of atoms in the chain (#), which includes the number of alkyl carbons (*n*) plus three atoms reflecting the acetamide residue (total # = *n* + 3). Finally, the addition of the letter A reflects the inclusion of amide functionality in the chain, resulting in a format of **PmP-#A,#A**. Detailed synthetic procedures and compound characterization data are provided in the Supporting Information. To our surprise, synthetic investigations using *N*-alkyl-2-bromoacetamide building blocks led to unpredictable production of impurities in addition to the desired product. The similarity in structural features of the impurities to the product as determined by ^1^H NMR spectroscopy is suspected to arise from counterion modification, as noted in a previous report.^24^ Attempts to avoid the formation of these impurities or separate these impurities from the desired products were unsuccessful in our hands.

**Figure 2:**
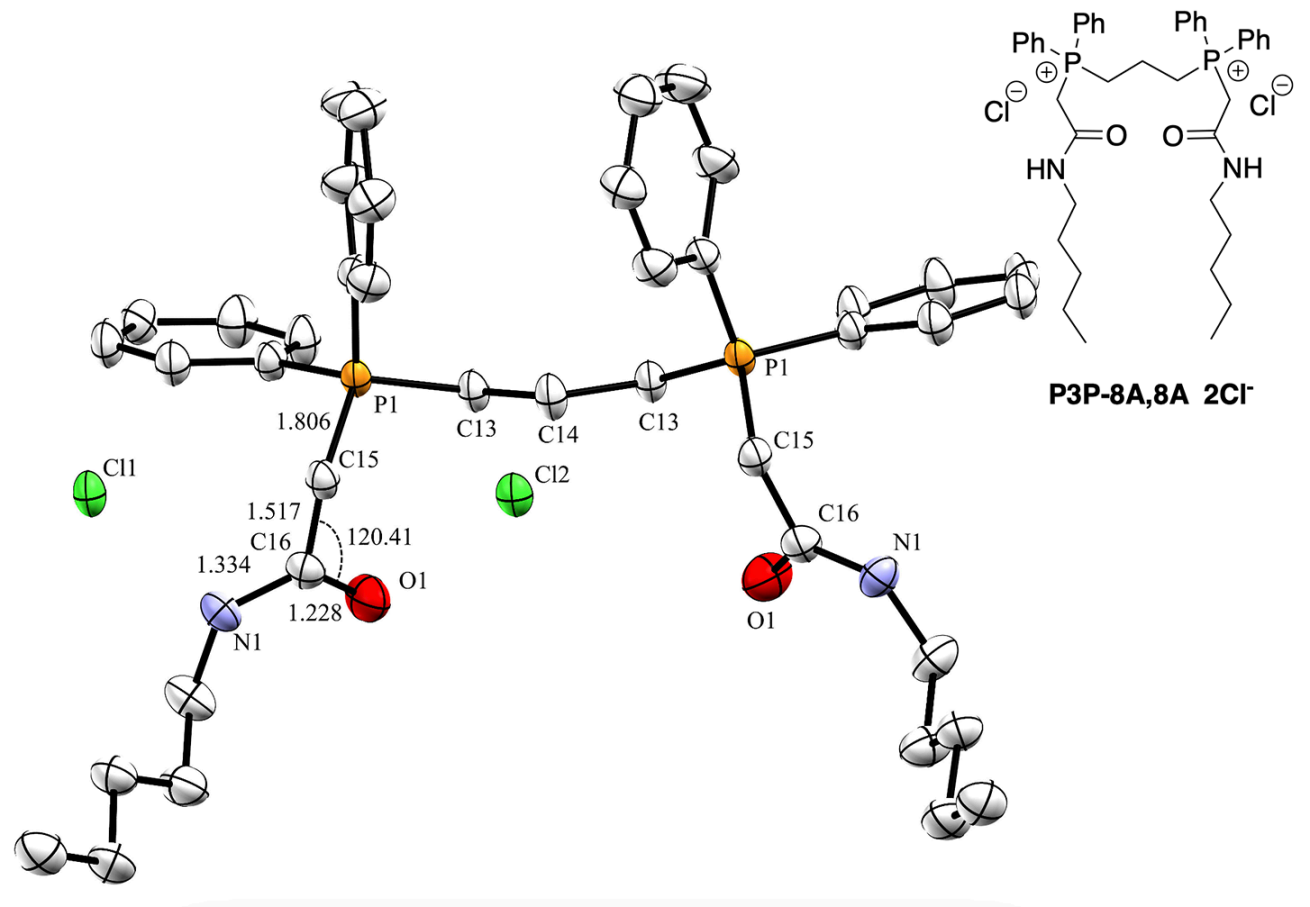
ORTEP diagrams for P3P-8A,8A (CCDC reference number 2196105). The atoms in the structure are color coded using the system of white (C), orange (P), green (Cl), red (O), and purple (N). Bond lengths for select P–C, N–C, O–C, and C–C bonds are labeled in Å, and the O–C–C and angle for the amide is labeled in degrees. Thermal ellipsoids are shown at the 50% probability level. Hydrogens and cocrystallized solvents are omitted for clarity.

To obtain further structural insight into these compounds, colorless needle crystals of P3P-8A,8A suitable for single-crystal X-ray analysis were grown by layering of diethyl ether onto a solution of the compound in acetonitrile at room temperature (Figure 2). Interestingly, in the solid state, the two nonpolar groups are disposed in a nearly parallel trajectory, and two of the aromatic rings are nearly coplanar. Evaluation of the experimental bond angles and lengths for the amide groups present in P3P-8A,8A demonstrates a structure which follows suit with a simple model amide, formamide.^25^ This structural similarity indicates that the amide is unperturbed by the proximity of the phosphonium center and, by extension, should maintain the expected hydrolyzable functionality of the amide.^26^

The stability of these compounds was assessed through exposure of P3P-9A,9A and P6P-9A,9A to deionized water as well as buffered solutions of pH 4, 6, 7, and 10.

**Figure 3:**
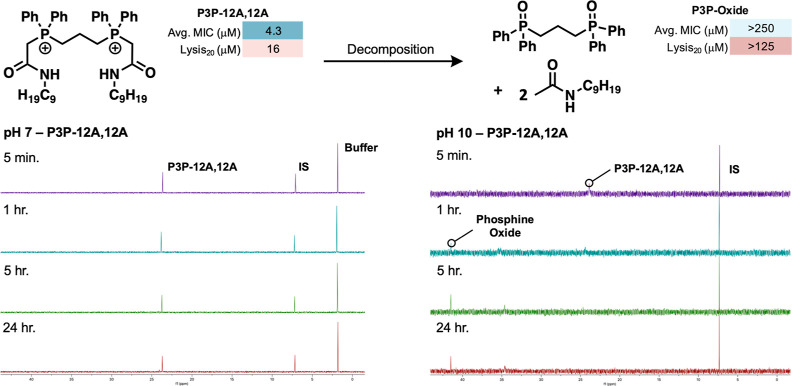
Top: Illustration of change in bioactivity before and after decomposition, from P3P-12A,12A to hydrolysis product, P3P-oxide. Bottom: representative hydrolysis experiment, as monitored by ^31^P NMR spectroscopy. Left: exposure of P3P-12A,12A to pH = 7 buffer and monitoring over 24 h. Right: exposure of P3P-12A,12A to pH = 10 buffer and monitoring over 24 h. IS = internal standard, sodium hypophosphate pentahydrate.

These conditions were selected to reflect different environmental conditions, including the variable pH of the soil. Decomposition was determined using ^31^P NMR spectroscopy through the qualitative loss of QPC product signal. Solutions were prepared at a concentration of 10 mg/mL of sample with 1 mg/mL sodium hypophosphate pentahydrate (NaH_2_PO_2_·5H_2_O) used as a nonreactive phosphorus-containing internal standard. We hypothesized that the soft QPCs would degrade rapidly under acidic conditions due to expected reactivity at the amide and similar to the fate of soft QACs^15^ while displaying relative stability in neutral and basic conditions. However, instead of observing the decomposition of the QPCs at pH = 4, both P3P-9A,9A and P6P-9A,9A immediately precipitated out of solution leaving no observable ^31^P NMR signals for product or decomposition products (for full details, see Supporting Information). ^1^H NMR spectroscopy and LCMS analysis confirmed that the precipitate was the corresponding intact P3P-9A,9A and P6P-9A,9A products: no decomposition products were observed from amide hydrolysis. At the more mildly acidic conditions of pH = 6 and neutral conditions of pH = 7, both P3P-9A,9A and P6P-9A,9A remained unchanged over the 24 h testing window. Under basic conditions of pH = 10, P3P-9A,9A showed the emergence of a new phosphorus signal at the 5 h mark which continued to develop over 24 h (Figure S4). Interestingly and in contrast to P3P-9A,9A and our previous amide-containing amphiphile results,^15^ a ∼24 h stability was observed for P6P-9A,9A at pH = 10 (Figure S8). Over the course of a week, the samples of P6P-9A,9A at pH = 10 began to form a precipitate which was identified by LCMS as the oxidized dpph. In light of these observations for P3P-9A,9A and P6P-9A,9A the stability studies were repeated for P3P-12A,12A and P6P-12A,12A under neutral (pH = 7) and basic (pH = 10) conditions. In neutral conditions, both P3P-12A,12A and P6P-12A,12A remained unchanged over a 24 h period (Figure 3, bottom left and S11). Under basic conditions, both P3P-12A,12A and P6P-12A,12A completely decomposed within 1 h and 5 h, respectively (Figure 3, bottom right and S12). In support of these ^31^P NMR data, LCMS analysis indicated that the soft QPCs underwent hydrolysis at the phosphonium center rather than at the amide; the amide side chain was in fact observed completely intact (Figures S13 and S14). The major phosphorus decomposition product was identified to be a phosphine oxide (Figure 3, bottom). Both the P3P- and P6P-phosphine oxides were prepared according to previous literature reports^27,28^ and were confirmed to be a match in both the mass and the elution time observed in the LCMS for the decomposition products. The behavior of these compounds at different pH levels suggests at least two possible treatment avenues to remediate persistence of these QPCs in aqueous waste streams: filtration of precipitated products at low pH and decomposition at higher pH. Prior reports using photocatalytic decomposition methods have indicated a greater susceptibility for QPC degradation compared to QACs, suggesting a third potential decomposition pathway for these materials outside of the scope tested herein.^29^

The bioactivities of the compounds were assessed via MIC and hemolysis (lysis_20_) assays, wherein the latter was used as a proxy for cytotoxicity. In addition to the soft bisQPC compounds, our previously synthesized best-in-class bisQPC (P6P-10,10)^22^ and two commercial QACs [BAC (70% benzyldimethyldodecylammonium chloride and 30% benzyldimethyltetradecylammonium chloride) and didecyldimethylammonium chloride (DDAC)] were used as controls for comparison. To determine the MIC values, the compounds were each screened against a panel of seven bacterial strains, including four Gram-positive strains [methicillin-susceptible *Staphylococcus aureus* (MSSA; SH1000), community-acquired methicillin-resistant *S. aureus* (CA-MRSA; USA 300-0114), hospital-acquired methicillin-resistant *S. aureus* (HA-MRSA; ATCC 33591), and *Enterococcus faecalis* (OG1RF)], as well as three Gram-negative strains [*Escherichia coli* (MC41000, *Acinetobacter baumannii* (ATCC 17948), and *Pseudomonas aeruginosa* (PAO1)]. To evaluate the hemolysis activities, the compound concentrations resulting in 20% red blood cell (RBC) lysis (lysis_20_) were determined. The results of the MIC and hemolysis assays are reported below in Table 1.

**Table 1:**
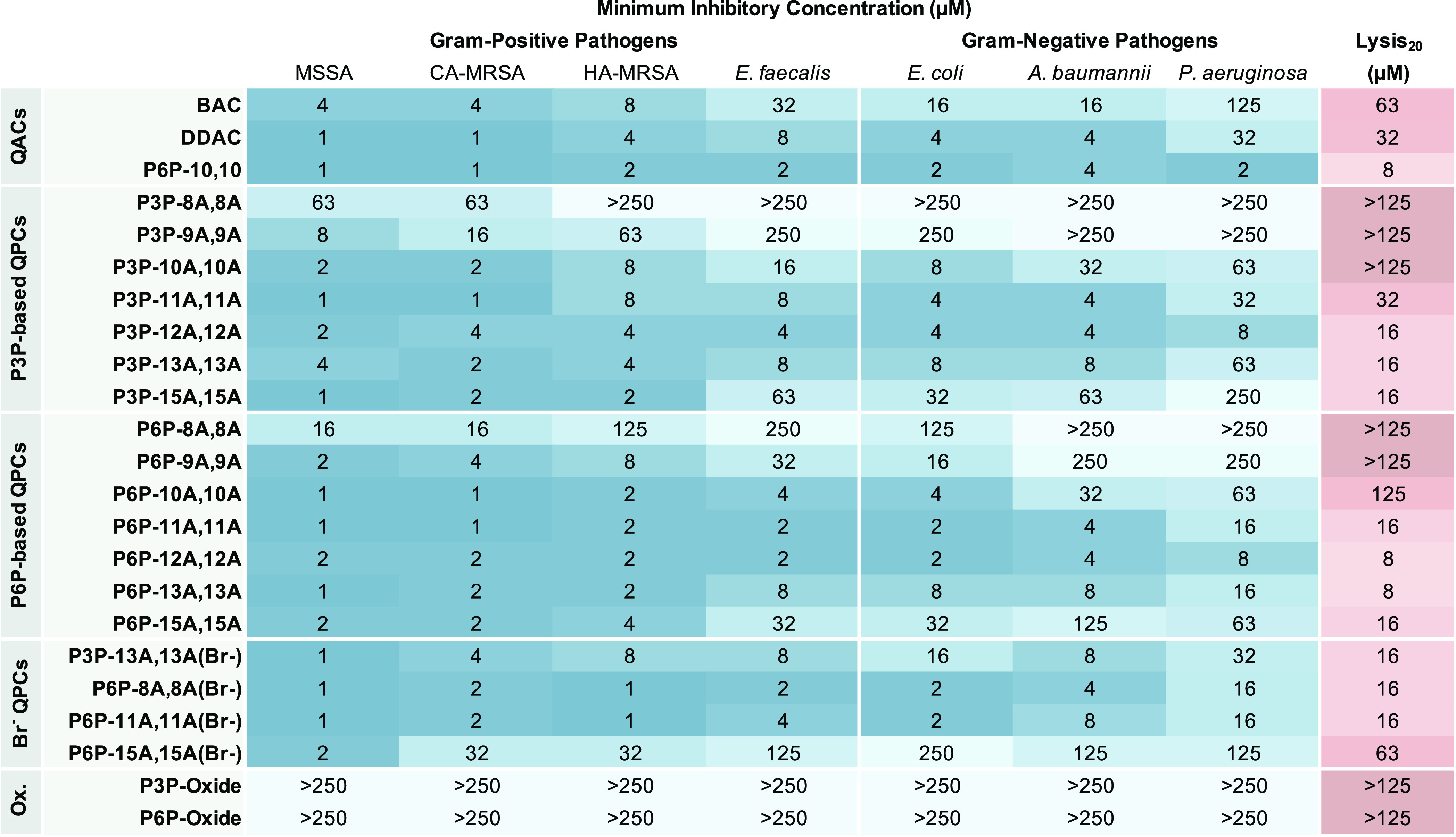
Antimicrobial Activity (MIC) and Hemolysis (lysis_20_) of the Prepared bisQPCs Compared to Commercially Available QACs against Gram-Positive Strains MSSA, CA-MRSA, HA-MRSA, and *E. faecalis*, as well as Gram-Negative Strains *E. coli*, *A. baumannii*, and *P. aeruginosa*

Several interesting bioactivity trends emerge for these soft QPCs. Strong antimicrobial activities were observed for many compounds, most notably P3P-12A,12A and P6P-12A,12A. The strongest determinant of biological activity in the amphiphilic structures was the length of the nonpolar side chain, wherein amphiphiles with 12-carbon side chains displayed the most potent activities, consistent with several prior reports.^13,14^ As noted previously with bisQPCs,^21^ there is a small but measurable advantage in the bioactivity of the 6-carbon linker (P6P) over the 3-carbon linker (P3P), as P6P-12A,12A boasts MICs between 2 and 8 μM against the entire panel of bacteria and compares quite favorably against the three controls. P6P-12A,12A has improved performance over BAC, particularly against Gram-negative pathogens, with 4–16× greater activity. The potency of P6P-12A,12A is narrowly improved compared to DDAC and approaches the performance of our best amphiphile prepared to date, P6P-10,10.^22^ To our delight, our top compounds P3P-12A,12A and P6P-12A,12A also performed well against resistant Gram-positive *S. aureus* strains as well, with no increase in MIC values for either HA- or CA-MRSA as compared to MSSA.

To assess the bioactivity of these novel soft QPCs more thoroughly, we investigated their ability to eradicate preassembled biofilms and made comparison to both a standard QPC recently reported by our group as well as a commercial standard known to successfully eradicate biofilms, DDAC. We were pleased to observe strong activity in the eradication of Gram-positive HA-MRSA by all QPCs tested at a level slightly preferable to DDAC (Table 2). Interestingly, all compounds tested showed weak inhibition of Gram-negative *P. aeruginosa* biofilms.

**Table 2:** Minimum Biofilm Eradication Concentration Assessment of Three QPCs and a Commercial Standard

	MBEC (μM)	
compounds	HA-MRSA	*P. aeruginosa*
P3P-12A,12A	4	>250
P6P-12A,12A	4	>250
^Me^P2P-12,12	4	>250
DDAC	8	250

Next, probing the cytotoxicity of the molecules, the lysis_20_ concentration for each compound was also determined. A general correlation between bioactivity and toxicity is observed, with the compounds having the most effective bioactivity also having a strong RBC lysis. However, a few compounds indicated a more measured balance between bioactivity and RBC lysis, which could indicate that it may be possible to tailor these compounds further to have high antimicrobial efficacy with lower risks of toxicity. For example, P6P-10A,10A did not demonstrate strong RBC lysis (125 μM), yet maintained low micromolar activity against the bacterial panel. Therefore, to optimize the balance between antimicrobial activity and low mammalian cytotoxicity, compounds of somewhat shorter chain length (# = 9–10) and/or compound mixtures may be exploited. Finally, it should be noted that the lysis_20_ values of the hydrolysis products (i.e., the phosphine oxides of P3P and P6P) were found to be >125 μM, reflecting only modest toxicity in this assay; the antibacterial activity was likewise not observed at the concentrations tested. These noticeable bioactivity differences between the bisQPCs and the decomposition products are viewed as desirable trends toward the goal of mitigating the development of antimicrobial resistance mechanisms due to environmental persistence. In addition to RBC lysis, mitochondrial toxicity was assessed. Quaternary phosphonium species are known to target the mitochondria of human cells.^30^ Due to this known activity, two of the best-in-class soft QPCs, P6P-12A,12A and P3P-12A,12A, were tested for mitochondrial toxicity in human hepatocellular carcinoma cells (HepG2). Pleasingly, no mitochondrial toxicity was observed for the compounds tested (see Figure S23 and Table S2). This is particularly gratifying since some indication of toxicity of certain phosphonium compounds to aquatic flora and fauna has been reported.^31^

## Conclusions

In summary, we have prepared a series of 14 novel biscationic amphiphilic structures, taking advantage of recent advances in the preparation of phosphonium-based structures as soft amphiphiles. Multiple compounds with amide-bearing side chains demonstrated single-digit micromolar antimicrobial activity and biofilm eradication ability, highlighted by the most effective compounds in this series, P6P-12A,12A and P3P-12A,12A. These compounds demonstrate desirable soft characteristics with stabilities of at least 24 h in deionized water and pH = 7 buffer, as would be intended during product use as a disinfectant, but hydrolyze upon longer exposure to basic conditions. With observed basic hydrolysis at the phosphonium center, these compounds are clearly susceptible to environmental breakdown. The observed hydrolysis products, P3P-oxide and P6P-oxide, showed no bioactivity or toxicity at the levels tested, indicating complete deactivation of the bisQPCs under hydrolysis conditions. With designed instability, options for toxicity minimization, and a broad suite of antimicrobial activity, the prepared soft QPCs represent a promising class of soft antimicrobial structures.

## Methods

### General Information

Reagents and solvents were used from Sigma-Aldrich, Acros Organics, TCI Chemicals, and Thermo Fisher Scientific without further purification. Reactions containing phosphorus starting materials were carried out under an argon atmosphere, with reagent grade solvents and magnetic stirring. All yields refer to spectroscopically pure compounds. ^1^H, ^13^C, and ^31^P NMR spectra were measured with a 400 MHz or 500 MHz JEOL spectrophotometer, and chemical shifts were reported on a δ-scale (ppm) downfield from TMS or 85% H_3_PO_4_. Coupling constants were calculated in hertz (Hz). The solvent used was chloroform-*d* (CDCl_3_) using the residual solvent peak as an internal reference of 7.26 ppm for ^1^H NMR and 77.16 ppm for ^13^C NMR. Accurate mass spectrometry data were acquired on an AB Sciex 5600 TripleTOF using electrospray ionization in the positive mode. All cell lines were acquired from the ATCC.

### Representative Synthesis: P6P-12A,12A

To a solution of 1,6-bis(diphenylphosphino)hexane (0.355 g, 1.00 mmol) in acetonitrile (5 mL) was added 2-chloro-*N*-nonylacetamide (0.393 g, 2.10 mmol). The solution was flushed with argon, heated to reflux, and stirred for 21 h. After cooling to room temperature, the excess solvent was removed from the flask using rotary evaporation. The resulting solid was triturated with cyclohexane (∼15 mL) at 45 °C for 1 h and then isolated by vacuum filtration, resulting in **P6P-12A,12A** as an orange, flaky solid (0.684 g, 97.9%); ^1^H NMR (400 MHz, chloroform-*d*): δ 9.24 (t, *J* = 6.0 Hz, 2H), 8.00–7.94 (m, 8H), 7.73–7.69 (m, 4H), 7.64–7.59 (m, 8H), 4.69 (d, *J* = 14.3 Hz, 4H), 3.16 (m, 4H), 3.00 (q, *J* = 6.2 Hz, 4H), 1.60 (s, 8H), 1.28–1.13 (m, 28H), 0.84 (t, *J* = 6.9 Hz, 6H); ^13^C{^1^H} NMR (100.6 MHz, chloroform-*d*), δ 162.8 (d, *J* = 5.3 Hz), 134.7 (d, *J* = 2.9 Hz), 133.5 (d, *J* = 10.1 Hz), 130.1 (d, *J* = 13.0 Hz), 118.4 (d, *J* = 85.3 Hz), 40.2, 31.9, 29.9 (d, *J* = 52.5 Hz), 29.5, 29.34, 29.31, 29.1, 28.7 (d, *J* = 16.9 Hz), 27.0, 22.6 (d, *J* = 51.1 Hz), 22.7, 21.1, 14.2; ^31^P{^1^H} NMR (162.0 MHz, chloroform-*d*): δ 25.58. HRMS (ESI+): 411.2694, C_52_H_76_N_2_O_2_P_2_ [M – 2Cl]^2+^ requires 411.2686.

### Biological Assays

For all biological assays, laboratory strains of MSSA (SH1000), *E. faecalis* (OG1RF), *E. coli* (MC4100), *P. aeruginosa* (PAO1), *A. baumannii* (ATCC 17948), CA-MRSA (USA300-0114), and HA-MRSA (ATCC 33591) were grown with shaking at 37 °C overnight from freezer stocks in 5 mL of the indicated media: SH1000, OG1RF, MC4100, USA300-0114, and PAO1 were grown in BD Mueller–Hinton broth (MHB), whereas ATCC 33591 was grown in BD tryptic soy broth (TSB). Optical density (OD) measurements were obtained using a SpectraMax iD3 plate reader (Molecular Devices, United States).

### MIC

Compounds were serially diluted 2-fold from stock solutions (1.0 mM) to yield 12 100 μL test concentrations, wherein the starting concentration of DMSO was 2.5%. Overnight, *S. aureus*, *E. faecalis*, *E. coli*, *P. aeruginosa*, *A. baumannii*, USA300-0114 (CA-MRSA), and ATCC 33591 (HA-MRSA) cultures were diluted to ca. 106 CFU/mL in MHB or TSB and regrown to midexponential phase, as determined by OD recorded at 600 nm (OD600). All cultures were then diluted again to *ca.* 106 CFU/mL and 100 μL and were inoculated into each well of a U-bottom 96-well plate containing 100 μL of compound solution. Plates were incubated statically at 37 °C for 48 h upon which wells were evaluated visually for bacterial growth. The MIC was determined as the lowest concentration of compound resulting in no bacterial growth visible to the naked eye based on the highest value in three independent experiments. Aqueous DMSO controls were conducted as appropriate for each compound.

### RBC Lysis Assay (lysis_20_)

RBC lysis assays were performed on mechanically defibrinated sheep blood (Hemostat Labs: DSB030). An aliquot of 1.5 mL blood was placed into a microcentrifuge tube and centrifuged at 3800 rpm for 10 min. The supernatant was removed, and the cells were resuspended with 1 mL of phosphate-buffered saline (PBS). The suspension was centrifuged as described above, the supernatant was removed, and the cells were resuspended four additional times in 1 mL of PBS. The final cell suspension was diluted 20-fold with PBS. Compounds were serially diluted with PBS 2-fold from stock solutions (1.0 mM) to yield 100 μL of 12 welve test concentrations on a flat-bottom 96-well plate (Corning, 351172), wherein the starting concentration of DMSO was 2.5%. To each of the wells, 100 μL of the 20-fold suspension dilution was then inoculated. The concentration of DMSO in the first well was 2.5%, resulting in DMSO-induced lysis at all concentrations > 63 μM. TritonX (1% by volume) served as a positive control (100% lysis marker), and sterile PBS served as a negative control (0% lysis marker). Samples were then placed in an incubator at 37 °C and shaken at 200 rpm. After 1 h, the samples were centrifuged at 3,800 rpm for 10 min. The absorbance of the supernatant was measured with a UV spectrometer at 540 nm wavelength. The concentration inducing 20% RBC lysis was then calculated for each compound based upon the absorbances of the TritonX and PBS controls. Aqueous DMSO controls were conducted as appropriate for each compound.^32^

### Mitochondrial Toxicity Assay

Mitochondrial toxicity was evaluated using a Promega Mitochondrial ToxGlo kit. Human hepatocellular carcinoma cells (HepG2) were cultured in RPMI-1640 medium containing 10% FBS at 37 °C and 5% CO_2_. Cells were seeded at a density of 2500 cells/well in 384 well tissue culture plates in either glucose (10 mM) or galactose (10 mM) supplemented media and were incubated overnight to allow for cell adherence. Cells were rinsed and replaced with serum-free media prior to experimentation. The Mitochondrial ToxGlo assay was performed in accordance with the manufacturer’s instructions. Cells were incubated with test compounds for 90 min prior to assay per the manufacturer’s protocol. Cells were incubated for 30 min with a cell impermeable, fluorogenic substrate. Cell integrity was measured by subsequent fluorescence (Ex/Em 485/525 nm) produced by necrosis-associated protease activity upon the substrate. Lysis buffer was then added, and net ATP levels were determined by luminescence measurement from a luciferase reporter. Compounds were serially diluted 2-fold from stock solutions (1.0 mM) to yield 12 100 μL test concentrations, wherein the starting concentration of DMSO was 0.5%. Mitochondrial toxicity, as per the manufacturer guidelines, was defined as a greater than 20% decrease in the ATP measure with a less than 20% increase in cytotoxicity.

### Single-Crystal X-ray Crystallography

X-ray intensity data for P3P-8A,8A (CCDC reference number 2196105) were collected on a Rigaku XtaLAB Synergy-S diffractometer using an HyPix-6000HE HPC area detector. The data collection employed Cu Kα radiation (λ = 1.54184 Å), and the data were collected at a temperature of 100 K. The intensity data were integrated using CrysAlisPro,^33^ which produced a listing of unaveraged *F*^2^ and σ(*F*^2^) values. The structure solution was determined using SHELXT,^34^ and refinement was conducted using SHELXL^33^ with anisotropic refinement of the thermal parameters for non-hydrogen atoms. Hydrogen atoms were placed using a riding model and refined isotropically. Detailed crystallographic data for this structure can be obtained free of charge via e-mail: deposit@ccdc.cam.ac.uk, online at http://www.ccdc.cam.ac.uk/conts/retrieving.html, by fax: (+44) 1223-336-033, or by contacting the Cambridge Crystallographic Data Centre, 12 Union Road, Cambridge CB2 1EZ, UK.

### Biofilm Eradication Assay^35,36^

Biofilm eradication experiments were performed using a pegged-lid microtiter plate assay to determine the MBEC values for compounds of interest, as previously described.^1,2^ Briefly, 125 mL of midlog phase culture diluted to *ca.* 10^6^ CFU/mL in TSB was added to wells of flat-bottom 96-well plates (Thermo Scientific, 266120) and covered with a 96-pegged lid (Thermo Scientific, 445497). Plates were incubated to establish bacterial biofilms after incubation at 37 °C for 24 h. The pegged lid was then removed, washed with PBS, and transferred to another 96-well plate containing 2-fold serial dilutions of the test compounds (the “challenge plate”). The total volume in each well was 150 mL, comprising 75 mL of compound diluted in water/DMSO, with a starting DMSO concentration of 2.5%, and 75 mL of TSB. Plates were incubated statically at 37 °C for 24 h. Next, the pegged lids were transferred to a fresh 96-well plate containing 180 mL of TSB and incubated overnight at 37 °C. MBEC values were determined as the lowest test concentration that resulted in the eradicated biofilm (i.e., wells displaying no turbidity in the final plate).

## Supporting Information Available

Detailed synthetic procedures; characterization data; NMR spectra; decomposition data and detailed procedures; and crystallographic collection details (PDF)

## Abbreviations

QAC: quaternary ammonium compound
QPC: quaternary phosphonium compound
WWTP: wastewater treatment plant
BAC: benzalkonium chloride
NMR: nuclear magnetic resonance
dppp: 1,3-bis(diphenylphosphino)propane
dpph: 1,6-bis(diphenylphosphino)hexane
MSSA: methicillin-susceptible *S. aureus*
CA-MRSA: community-acquired methicillin-resistant *S. aureus*
HA-MRSA: hospital-acquired methicillin-resistant *S. aureus*
MIC: minimum inhibitory concentration
RBC: red blood cell
TSB: tryptic soy broth
OD: optical density
MHB: Mueller-Hinton broth
DMSO: dimethyl sulfoxide
PBS: phosphate-buffered saline
