# Supplementary material for: Soft QPCs: Biscationic Quaternary Phosphonium Compounds as Soft Antimicrobial Agents
Source: ACS Infect Dis. 2023 Mar 16;9(4):943–51. doi: 10.1021/acsinfecdis.2c00624 (PMC10111419; doi:10.1021/acsinfecdis.2c00624)
Supplement: Supplementary file 1 — id2c00624_si_001.pdf [file id2c00624_si_001.pdf]

Supporting Information

Soft QPCs: Biscationic Quaternary Phosphonium Compounds as Soft Antimicrobial Agents

Samantha R. Brayton,[a] Zachary E. A. Toles,[a] Christian A. Sanchez,[b] Marina E. Michaud,[b] Laura M. Thierer,[a] Taylor M. Keller,[c] Caitlin J. Risener,[d] Cassandra L. Quave,[e] William M. Wuest,*[b] Kevin P. C. Minbiole*[a]

[a]Department of Chemistry, Villanova University, Villanova, PA 19085, United States
[b]Department of Chemistry, Emory University, Atlanta, GA 30322, United States
[c]Department of Chemistry X-Ray Crystallography Facility, University of Pennsylvania, Philadelphia, PA 19104, United States
[d]Molecular and Systems Pharmacology Program, Emory University, Atlanta, GA 30322
[e]Department of Dermatology, Emory University School of Medicine, Atlanta, GA 30322, United States

*Corresponding authors
kevin.minbiole@villanova.edu
wwuest@emory.edu

Table of Contents

I. Decomposition Studies (S2)

II. Synthetic Procedures (S10)

III. NMR Spectra (S24)

IV. Crystallographic Data (S66)

V. Mitochondrial Toxicity Data (S67)

I. Decomposition Studies

Samples were analyzed using 400 MHz JEOL spectrophotometer, specifically focusing on ^{31}P NMR (162 Hz Larmor frequency for ^{31}P NMR). The internal standard was comprised of 2 mg/mL solution of sodium hypophosphite pentahydrate ($NaH_2PO_2•5H_2O$) in deionized water. To a 5 mL sample vial, 10 mg of each compound and 0.5 mL of the internal standard was added. The sample was vial was placed under hot running water for roughly 5 mins, and consecutively mixed on a Fischer Digital Vortex Mixer for 30 seconds. This process was repeated until all the material was in solution. A 0.5 mL aliquot of buffer was added to each sample vial and was promptly transferred to an NMR tube and then placed in the NMR for analysis. Data was collected at 5 minutes, 1 hour, 5 hours and 24 hours for each sample. This procedure was performed with buffers of pH = 4, 6, 7, and 10 (Figure S1-12). The organic components were extracted with dichloromethane and then characterized via HRMS; the decomposition products were determined by mass (Figure S13-14).

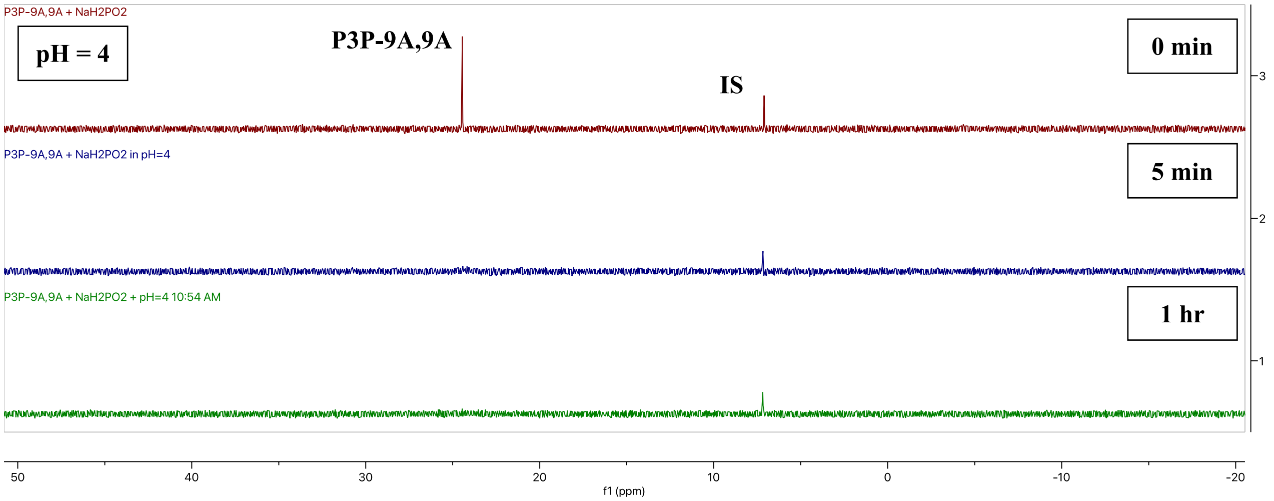

Figure S1. Sample decomposition graphs of P3P-9A,9A with internal standard at pH = 4.

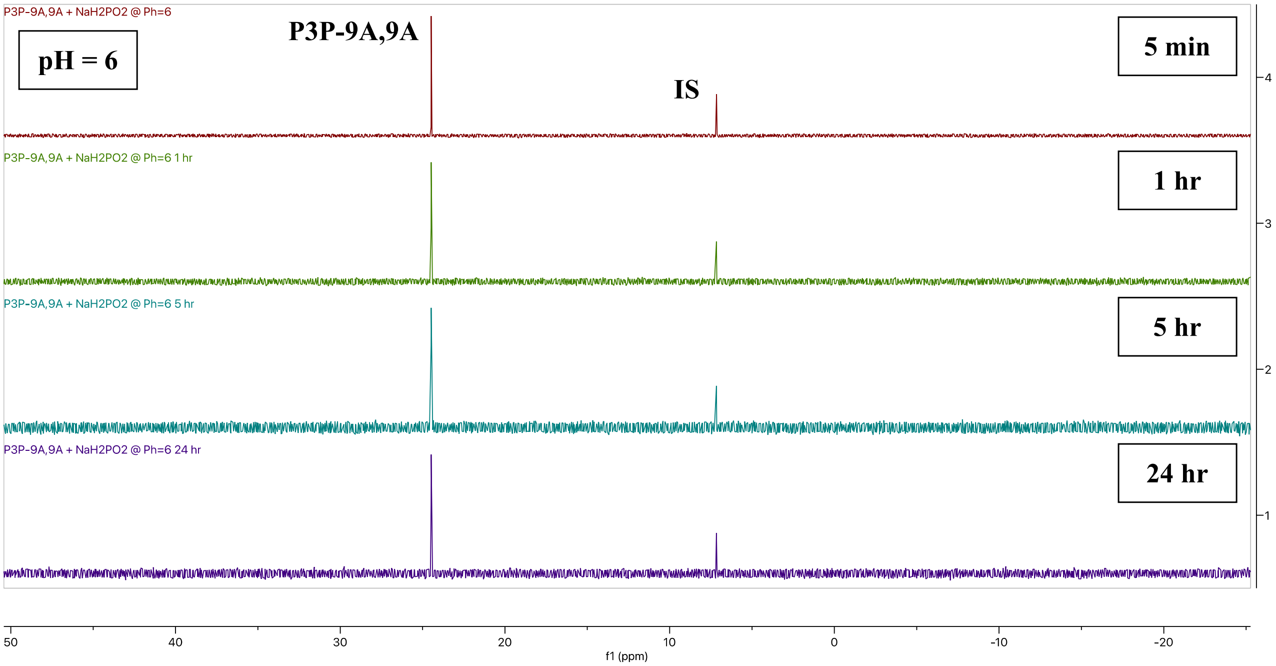

Figure S2. Sample decomposition graphs of P3P-9A,9A with internal standard at pH = 6.

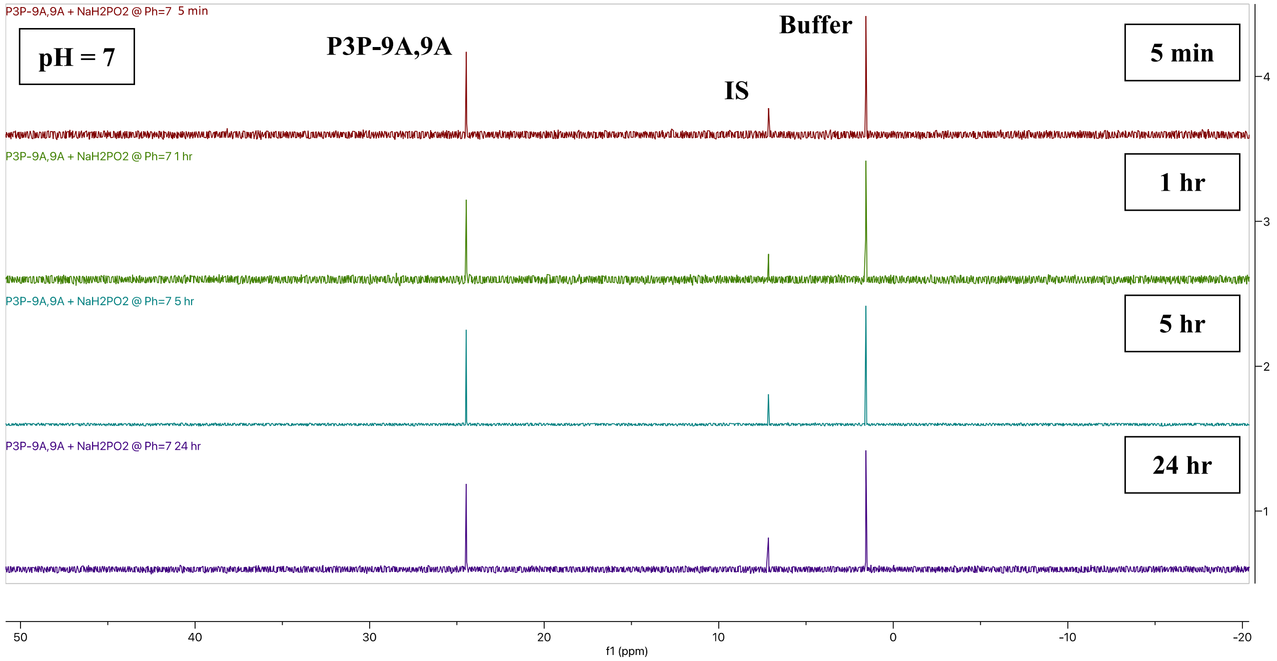

Figure S3. Sample decomposition graphs of P3P-9A,9A with internal standard at pH = 7.

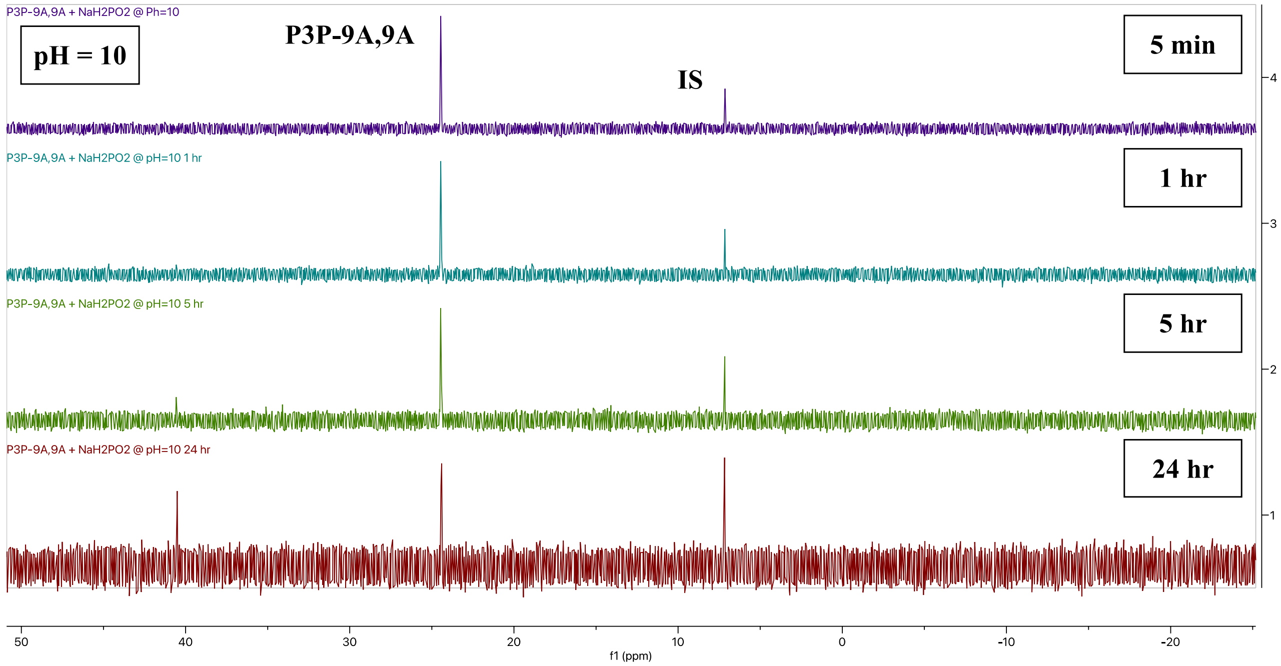

Figure S4. Sample decomposition graphs of P3P-9A,9A with internal standard at pH = 10.

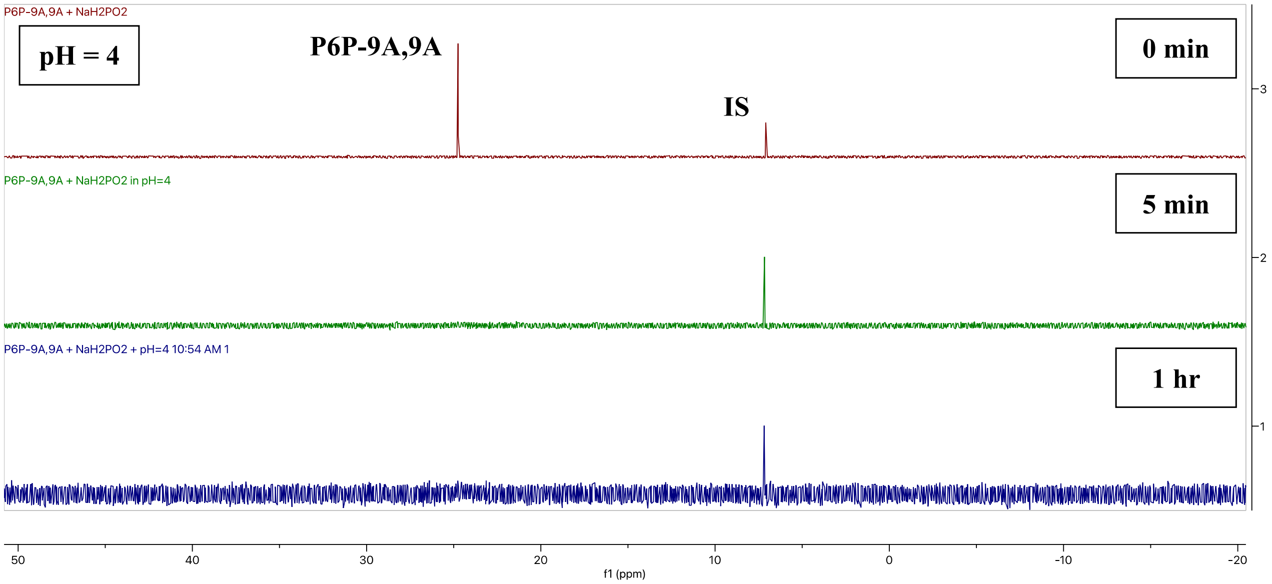

Figure S5. Sample decomposition graphs of P6P-9A,9A with internal standard at pH = 4.

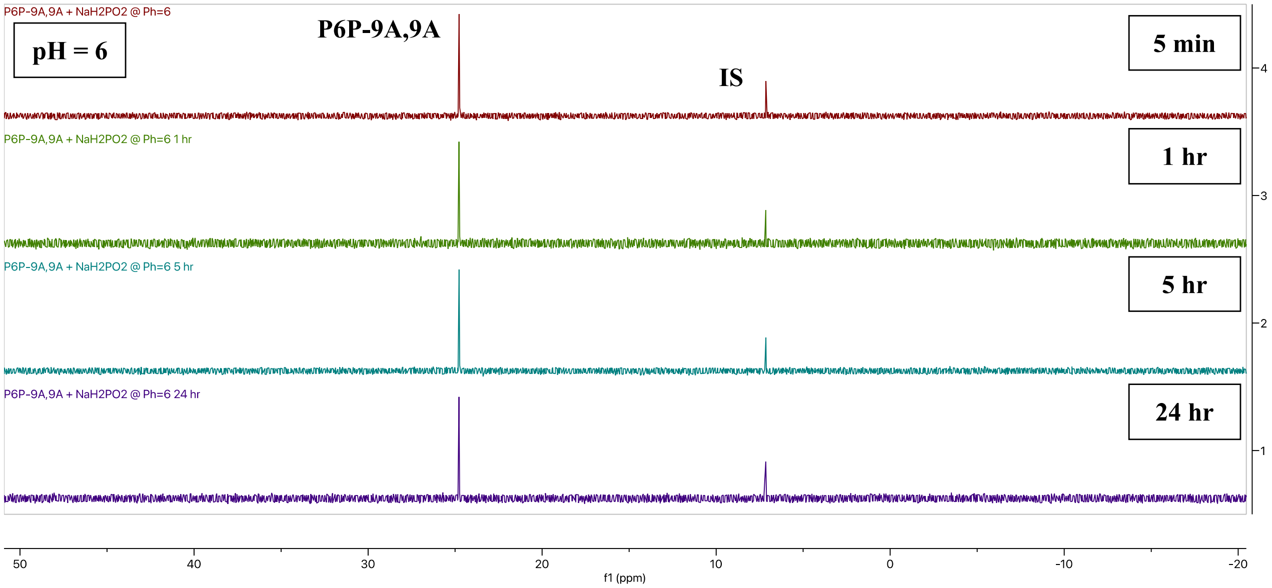

Figure S6. Sample decomposition graphs of P6P-9A,9A with internal standard at pH = 6.

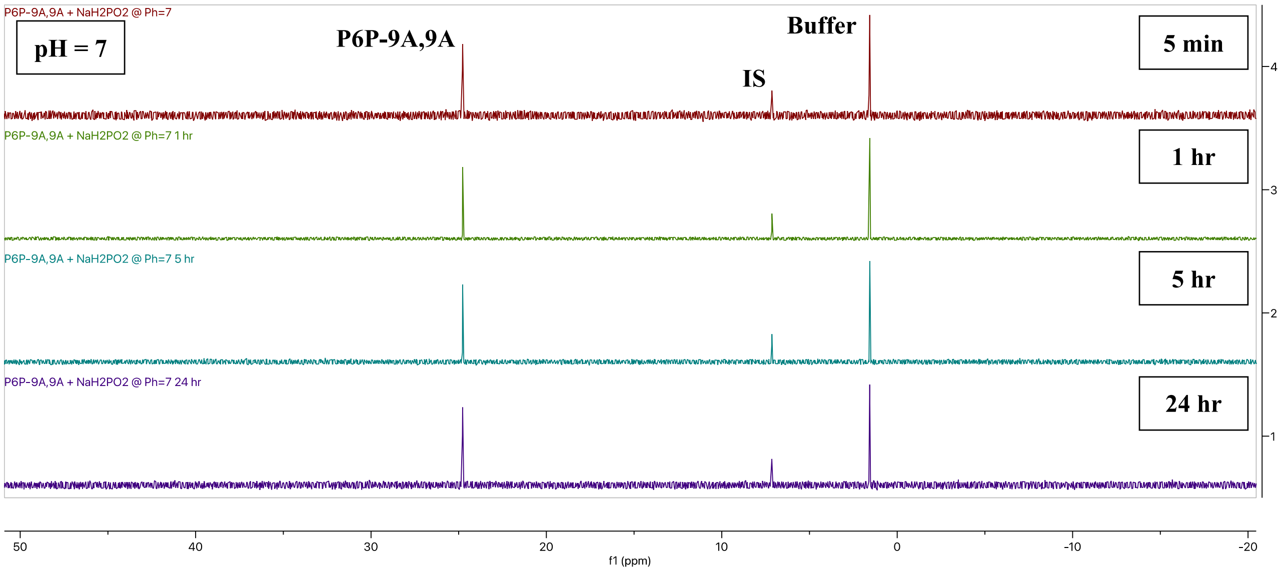

Figure S7. Sample decomposition graphs of P6P-9A,9A with internal standard at pH = 7.

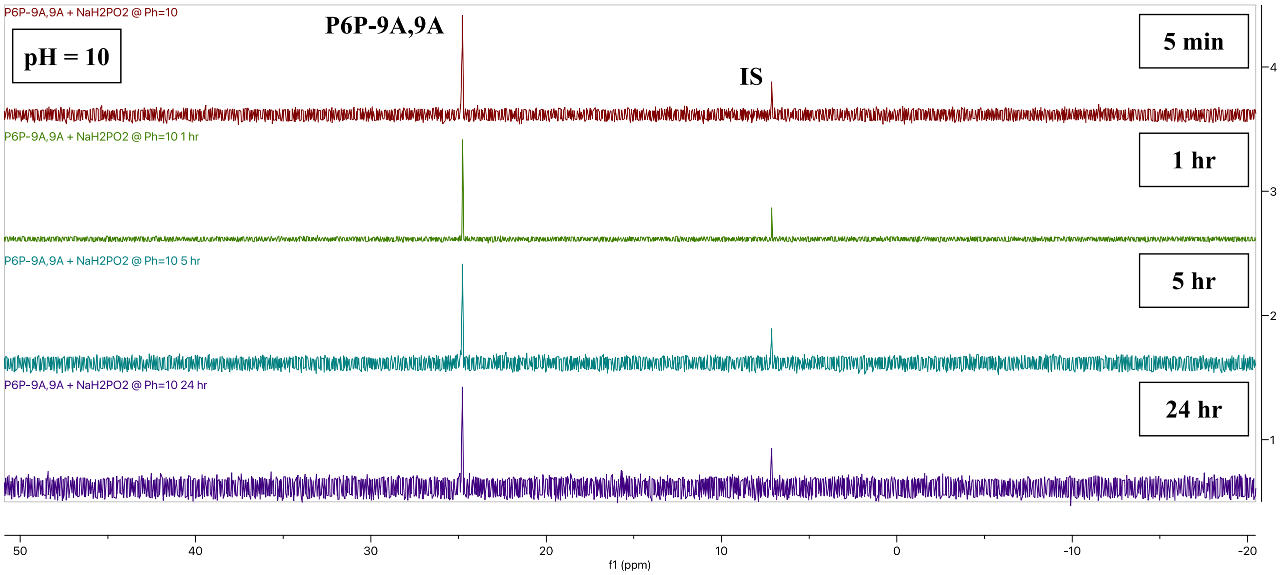

Figure S8. Sample decomposition graphs of P6P-9A,9A with internal standard at pH = 10.

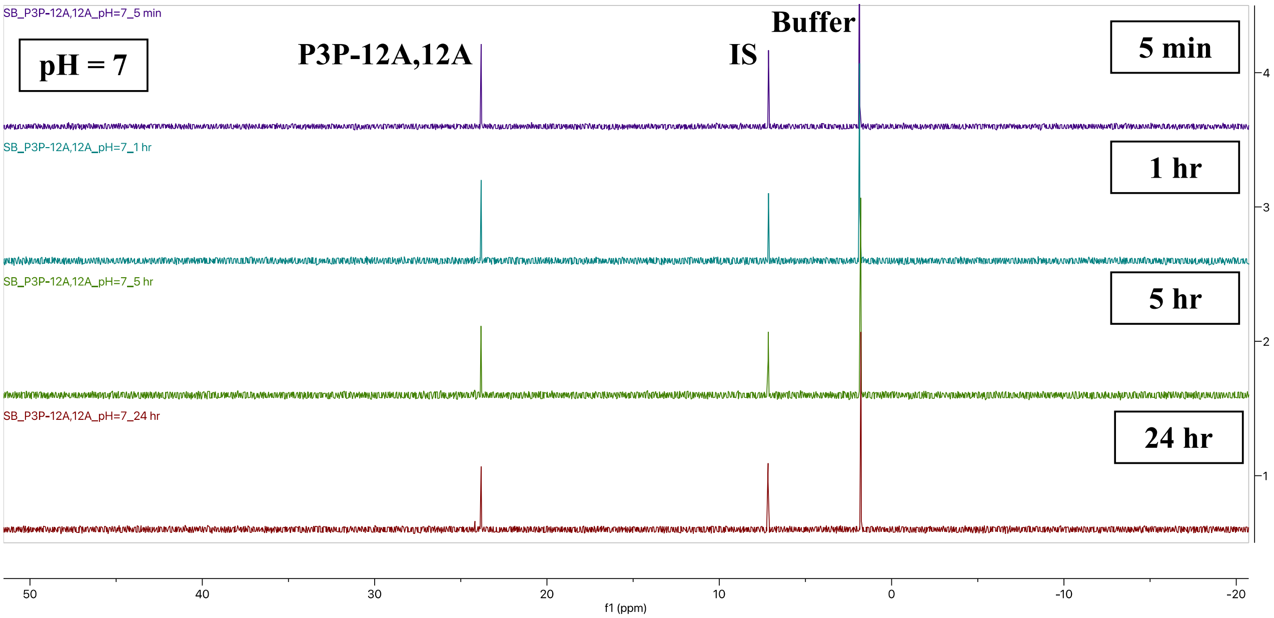

Figure S9. Sample decomposition graphs of P3P-12A,12A with internal standard at pH = 7.

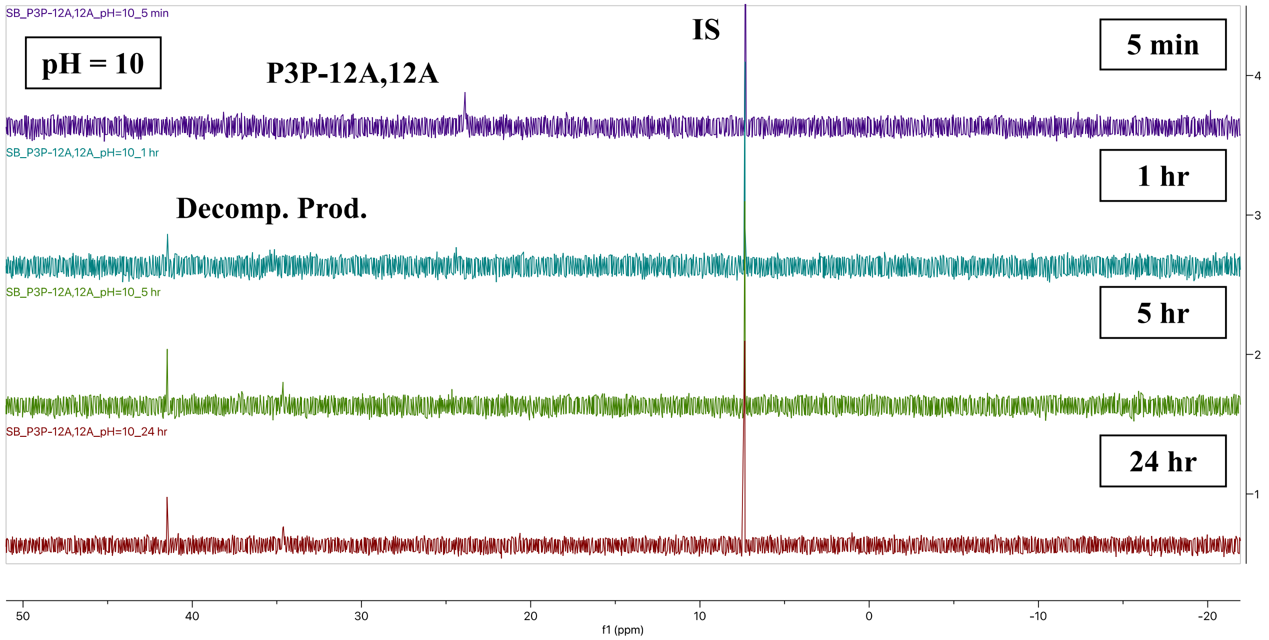

Figure S10. Sample decomposition graphs of P3P-12A,12A with internal standard at pH = 10.

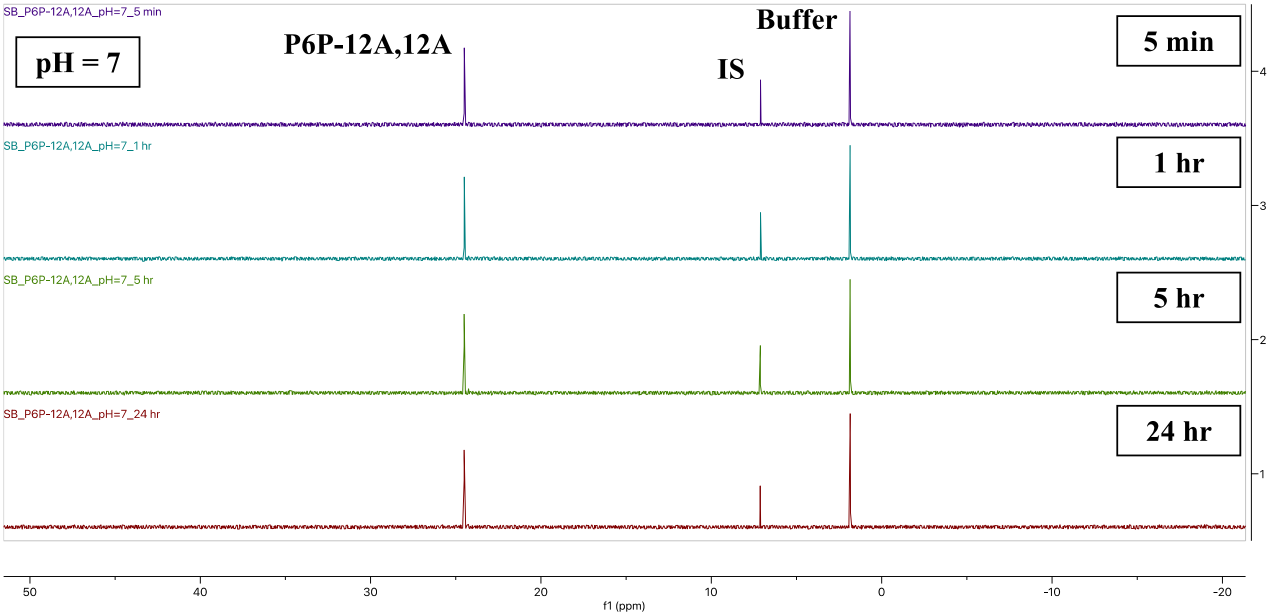

Figure S11. Sample decomposition graphs of P6P-12A,12A with internal standard at pH = 7.

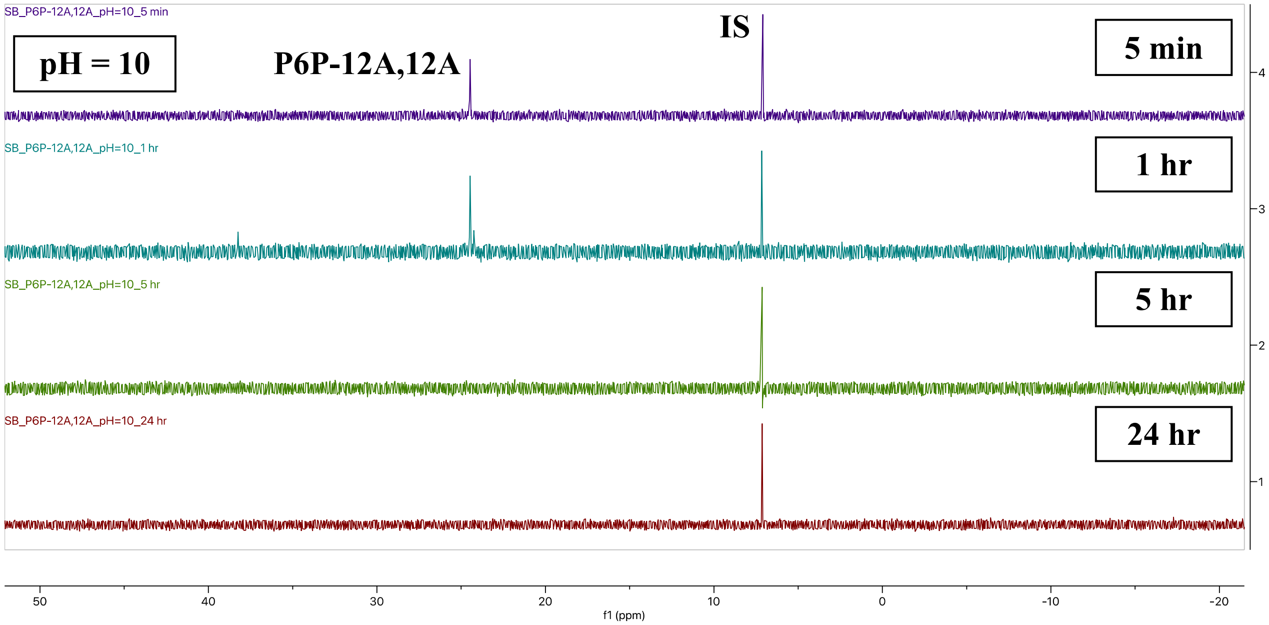

Figure S12. Sample decomposition graphs of P6P-12A,12A with internal standard at pH = 10.

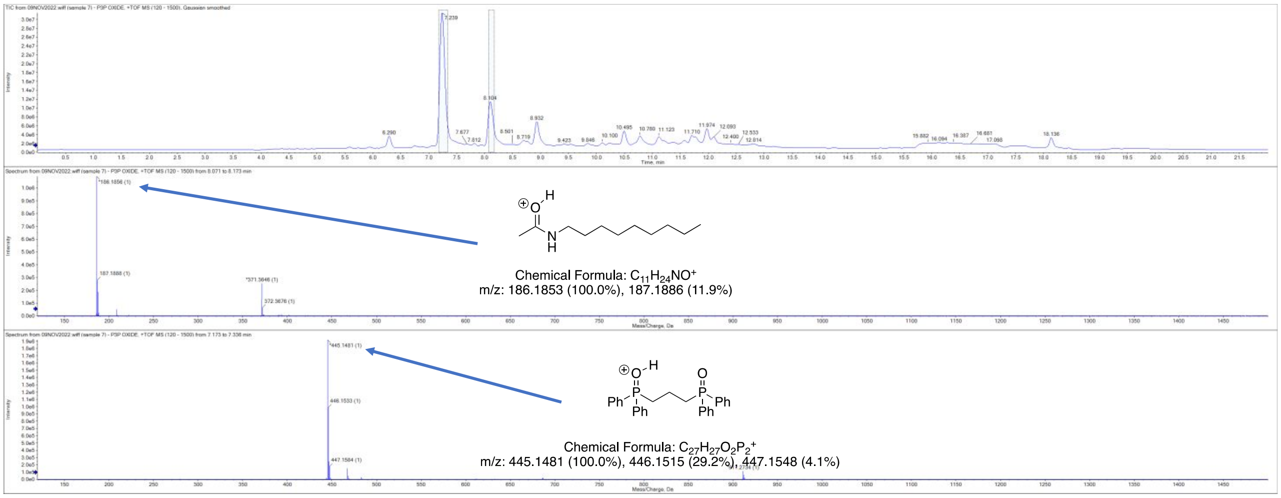

Figure S13. HRMS (ESI+) Total Ion Chromatogram of extracted P3P-12A,12A decomposition products.

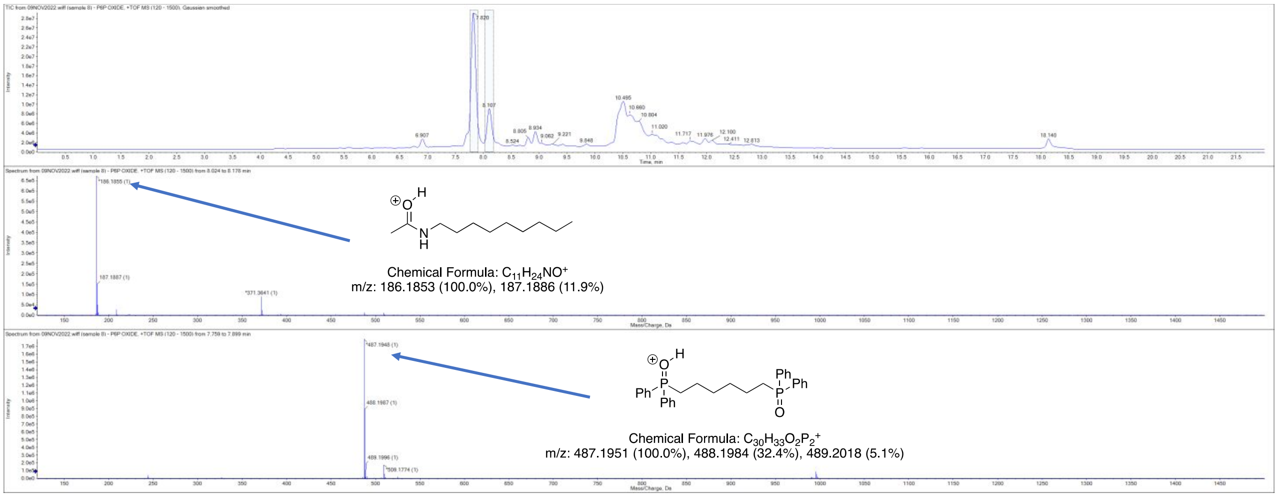

Figure S14. HRMS (ESI+) Total Ion Chromatogram of extracted P6P-12A,12A decomposition products.

II. Synthetic Procedures

Preparation of P3P-8A,8A

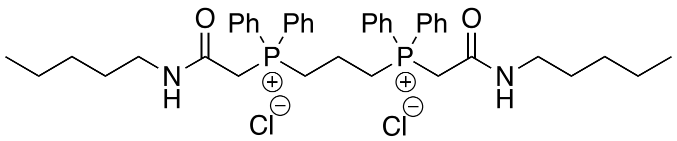

To a solution of 1,3-bis(diphenylphosphino)propane (0.357 g, 1.00 mmol) in acetonitrile (5 mL) was added 2-chloro-N-pentylacetamide (0.295 g, 2.10 mmol). The solution was flushed with argon, heated to reflux, and stirred for 24 hours. After cooling to room temperature, the excess solvent was removed from the flask using rotary evaporation. The resulting solid was triturated with cyclohexane (~15 mL) at 45 ˚C for 1 hour and then isolated by vacuum filtration, resulting in **P3P-8A,8A** as a white, powdery solid (0.533 g, 83.3%); ^{1}H NMR (400 MHz, chloroform-*d*), δ 8.88 (t, *J* = 6.3 Hz, 2H), 8.06-8.01 (m, 8H), 7.73-7.69 (m, 4H), 7.65-7.61 (m, 8H), 4.57 (d, *J* = 14.5 Hz, 4H), 3.74 (m, 4H), 2.93 (q, *J* = 6.7 Hz, 4H), 2.13 (m, 2H), 1.31-1.09 (m, 12H), 0.81 (t, *J* = 7.0 Hz, 6H); ^{13}C{^{1}H} NMR (100.6 MHz, chloroform-*d*), δ 162.5 (t, *J* = 2.9 Hz), 134.9, 133.5 (quint, *J* = 4.8 Hz), 130.2, 117.7 (d, *J* = 86.2 Hz), 40.1, 30.0 (d, *J* = 53.0 Hz), 29.0, 28.7, 23.4 (dd, *J* = 16.9, 53.9 Hz), 22.3, 16.2, 14.0; ^{31}P{^{1}H} NMR (162.0 MHz, chloroform-*d*) δ 24.04. HRMS (ESI+): 334.1834, $C_{41}H_{54}N_2O_2P_2$ $[M-2Cl]^{2+}$ requires 334.1825.

Preparation of P3P-9A,9A

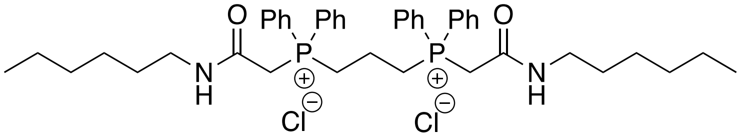

To a solution of 1,3-bis(diphenylphosphino)propane (0.355 g, 1.00 mmol) in acetonitrile (5 mL) was added 2-chloro-N-hexylacetamide (0.320 g, 2.10 mmol). The solution was flushed with argon, heated to reflux, and stirred for 24 hours. After cooling to room temperature, the excess solvent was removed from the flask using rotary evaporation. The resulting solid was triturated with cyclohexane (~15 mL) at 45 °C for 1 hour and then isolated by vacuum filtration, resulting in **P3P-9A,9A** as a white, powdery solid (0.446 g, 67.5%); ^{1}H NMR (400 MHz, chloroform-*d*), δ 8.88 (t, *J* = 6.0 Hz, 2H), 8.06-8.01 (m, 8H), 7.71-7.68 (m, 4H), 7.64-7.59 (m, 8H), 4.58 (d, *J* = 14.3 Hz, 4H), 3.73 (m, 4H), 2.92 (q, *J* = 7.0 Hz, 4H), 2.11 (m, 2H), 1.27-1.12 (m, 16H), 0.82 (t, *J* = 7.2 Hz, 6H); $^{13}C\{^{1}H\}$ NMR (100.6 MHz, chloroform-*d*), δ 162.5 (t, *J* = 2.4 Hz), 134.9, 133.5 (quint, *J* = 5.3 Hz), 130.2, 117.7 (d, *J* = 86.2 Hz), 40.2, 31.4, 30.0 (d, *J* = 52.5 Hz), 29.0, 26.6, 23.7 (dd, *J* = 16.9 Hz), 22.6, 16.2, 14.1; $^{31}P\{^{1}H\}$ NMR (162.0 MHz, chloroform-*d*) δ 24.19. HRMS (ESI+): 348.1991, $C_{43}H_{58}N_2O_2P_2$ $[M-2Cl]^{2+}$ requires 348.1982.

Preparation of P3P-10A,10A

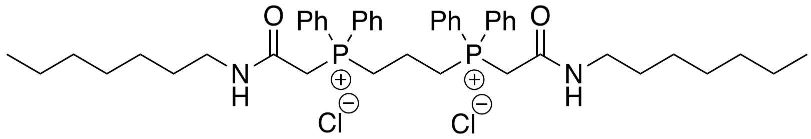

To a solution of 1,3-bis(diphenylphosphino)propane (0.365 g, 1.00 mmol) in acetonitrile (5 mL) was added 2-chloro-N-hepylacetamide (0.362 g, 2.10 mmol). The solution was flushed with argon, heated to reflux, and stirred for 24.5 hours. After cooling to room temperature, the excess solvent was removed from the flask using rotary evaporation. The resulting solid was triturated with cyclohexane (~15 mL) at 45 °C for 1 hour and then isolated by vacuum filtration, resulting in **P3P-10A,10A** as a white, powdery solid (0.590 g, 81.8%); ^{1}H NMR (400 MHz, chloroform-*d*), δ 8.87 (t, *J* = 5.7 Hz, 2H), 8.06-8.01 (m, 8H), 7.73-7.70 (m, 4H), 7.65-7.60 (m, 8H), 4.57 (d, *J* = 14.6 Hz, 4H), 3.73 (m, 4H), 2.92 (q, *J* = 6.1 Hz, 4H), 2.11 (m, 2H), 1.30-1.16 (m, 20H), 0.84 (t, *J* = 7.0 Hz, 6H); ^{13}C{^{1}H} NMR (100.6 MHz, chloroform-*d*), δ 162.4 (t, *J* = 2.4 Hz), 134.9, 133.5 (quint, *J* = 5.3 Hz), 130.2, 117.8 (d, *J* = 86.2 Hz), 40.2, 31.8, 30.0 (d, *J* = 53.0 Hz) 29.1, 28.9, 26.9, 23.4 (dd, *J* = 16.9 Hz) 22.6, 16.2, 14.2; ^{31}P{^{1}H} NMR (162.0 MHz, chloroform-*d*) δ 24.10. HRMS (ESI+): 362.2152, $C_{45}H_{62}N_2O_2P_2$ $[M-2Cl]^{2+}$ requires 362.2138.

Preparation of P3P-11A,11A

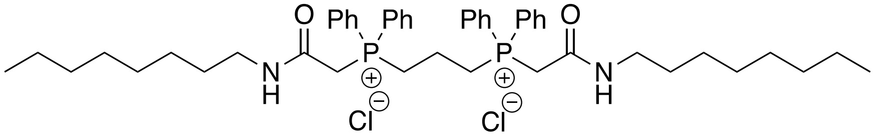

To a solution of 1,3-bis(diphenylphosphino)propane (0.361 g, 1.00 mmol) in acetonitrile (5 mL) was added 2-chloro-N-octylacetamide (0.371 g, 2.10 mmol). The solution was flushed with argon, heated to reflux, and stirred for 24 hours. After cooling to room temperature, the excess solvent was removed from the flask using rotary evaporation. The resulting solid was triturated with cyclohexane (~15 mL) at 45 °C for 1 hour and then isolated by vacuum filtration, resulting in **P3P-11A,11A** as a white, powdery solid (0.589 g, 83.3%); ^{1}H NMR (400 MHz, chloroform-*d*), δ 8.87 (t, J = 5.7 Hz, 2H), 8.06-8.01 (m, 8H), 7.73-7.69 (m, 4H), 7.65-7.60 (m, 8H), 4.57 (d, J = 14.3 Hz, 4H), 3.73 (m, 4H), 2.92 (q, J = 6.2 Hz, 4H), 2.11 (m, 2H), 1.28-1.15 (m, 24H), 0.85 (t, J = 6.9 Hz, 6H); ^{13}C{^{1}H} NMR (100.6 MHz, chloroform-*d*), δ 162.4 (t, J = 2.4 Hz), 134.9, 133.5 (quint, J = 4.8 Hz), 130.2, 117.7 (d, J = 86.2 Hz), 40.2, 31.9, 30.0 (d, J = 52.5 Hz), 29.3, 29.2, 29.1, 27.0, 23.5 (dd, J = 17.8, 53.5 Hz), 22.7, 16.2, 14.2; ^{31}P{^{1}H} NMR (162.0 MHz, chloroform-*d*) δ 24.09. HRMS (ESI+): 376.2301, $C_{47}H_{66}N_2O_2P_2$ $[M-2Cl]^{2+}$ requires 376.2295.

Preparation of P3P-12A,12A

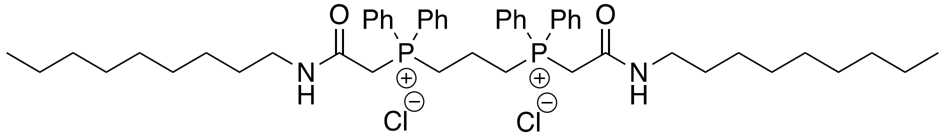

To a solution of 1,3-bis(diphenylphosphino)propane (0.357 g, 1.00 mmol) in acetonitrile (5 mL) was added 2-chloro-N-nonylacetamide (0.393 g, 2.10 mmol). The solution was flushed with argon, heated to reflux, and stirred for 20 hours. After cooling to room temperature, the excess solvent was removed from the flask using rotary evaporation. The resulting solid was triturated with cyclohexane (~15 mL) at 45 ˚C for 1 hour and then isolated by vacuum filtration, resulting in **P3P-12A,12A** as a yellow, flaky solid (0.458 g, 62.1%); ^{1}H NMR (400 MHz, chloroform-*d*), δ 8.87 (t, *J* = 5.5 Hz, 2H), 8.06-8.01 (m, 8H), 7.73-7.69 (m, 4H), 7.65-7.60 (m, 8H), 4.57 (d, *J* = 14.6 Hz, 4H), 3.74 (m, 4H), 2.92 (q, *J* = 6.2 Hz, 4H), 2.12 (m, 2H), 1.28-1.16 (m, 28H), 0.86 (t, *J* = 6.9 Hz, 6H); ^{13}C{^{1}H} NMR (100.6 MHz, chloroform-*d*), δ 162.4 (t, *J* = 2.9 Hz), 134.9, 133.5 (quint, *J* = 4.8 Hz), 130.2, 117.7 (d, *J* = 86.2 Hz), 40.2, 31.9, 30.0 (d, *J* = 53.0 Hz), 29.5, 29.33, 29.28, 29.1, 27.0, 23.4 (dd, *J* = 17.8, 54.0 Hz), 22.7, 16.2, 14.2; ^{31}P{^{1}H} NMR (162.0 MHz, chloroform-*d*) δ 24.08. HRMS (ESI+): 390.2458, $C_{49}H_{70}N_2O_2P_2$ $[M-2Cl]^{2+}$ requires 390.2451.

Preparation of P3P-13A,13A

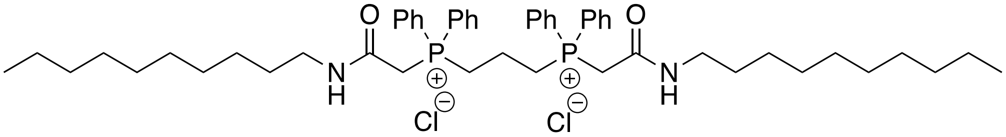

To a solution of 1,3-bis(diphenylphosphino)propane (0.378 g, 1.00 mmol) in acetonitrile (5 mL) was added 2-chloro-N-decylacetamide (0.434 g, 2.10 mmol). The solution was flushed with argon, heated to reflux, and stirred for 20 hours. After cooling to room temperature, the excess solvent was removed from the flask using rotary evaporation. The resulting solid was triturated with cyclohexane (~15 mL) at 45 °C for 1 hour and then isolated by vacuum filtration, resulting in **P3P-13A,13A** as a white, powdery solid (0.628 g, 77.9%); ^{1}H NMR (400 MHz, chloroform-*d*), δ 8.87 (t, *J* = 5.7 Hz, 2H), 8.06-8.01 (m, 8H), 7.73-7.68 (m, 4H), 7.65-7.60 (m, 8H), 4.57 (d, *J* = 14.4 Hz, 4H), 3.74 (m, 4H), 2.92 (q, *J* = 6.8 Hz, 4H), 2.13 (m, 2H), 1.25-1.16 (m, 32H), 0.86 (t, *J* = 5.7 Hz, 6H); ^{13}C{^{1}H} NMR (100.6 MHz, chloroform-*d*), δ 162.4 (t, *J* = 2.4 Hz), 134.9, 133.5 (quint, *J* = 4.8 Hz), 130.3, 117.7 (d, *J* = 86.2 Hz), 40.2, 32.0, 30.0 (d, *J* = 53.0 Hz), 29.63, 29.58, 29.4, 29.3, 29.1, 27.0, 23.4 (dd, *J* = 16.9, 53.5 Hz), 22.8, 16.2, 14.2; ^{31}P{^{1}H} NMR (162.0 MHz, chloroform-*d*) δ 24.04. HRMS (ESI+): 404.2620, $C_{51}H_{74}N_2O_2P_2$ $[M-2Cl]^{2+}$ requires 404.2608.

Preparation of P3P-15A,15A

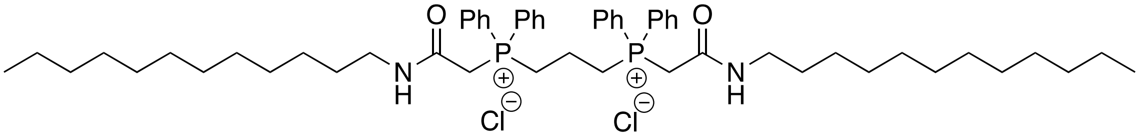

To a solution of 1,3-bis(diphenylphosphino)propane (0.354 g, 1.00 mmol) in acetonitrile (5 mL) was added 2-chloro-N-dodecylacetamide (0.469 g, 2.10 mmol). The solution was flushed with argon, heated to reflux, and stirred for 24.5 hours. After cooling to room temperature, the excess solvent was removed from the flask using rotary evaporation. The resulting solid was triturated with cyclohexane (~15 mL) at 45 °C for 1 hour and then isolated by vacuum filtration, resulting in **P3P-15A,15A** as a white, powdery solid (0.752 g, 93.5%); ^{1}H NMR (400 MHz, chloroform-*d*), δ 8.88 (t, *J* = 6.0 Hz, 2H), 8.06-8.01 (m, 8H), 7.72-7.68 (m, 4H), 7.64-7.60 (m, 8H), 4.58 (d, *J* = 14.4 Hz, 4H), 3.74 (m, 4H), 2.92 (q, *J* = 6.2 Hz, 4H), 2.13 (m, 2H), 1.28-1.15 (m, 40H), 0.86 (t, *J* = 6.8 Hz, 6H); ^{13}C{^{1}H} NMR (100.6 MHz, chloroform-*d*), δ 162.4 (t, *J* = 2.4 Hz), 134.9, 133.5 (quint, *J* = 4.3 Hz), 130.3, 117.7 (d, *J* = 86.2 Hz), 40.2, 32.0, 30.0 (d, *J* = 53.9 Hz), 29.73, 29.71, 29.69, 29.6, 29.4, 29.3, 29.1, 27.0, 23.4 (dd, *J* = 16.9, 53.9 Hz), 22.8, 16.2, 14.2; ^{31}P{^{1}H} NMR (162.0 MHz, chloroform-*d*) δ 24.08. HRMS (ESI+): 432.2929, $C_{55}H_{82}N_2O_2P_2$ $[M-2Cl]^{2+}$ requires 432.2921.

Preparation of P6P-8A,8A

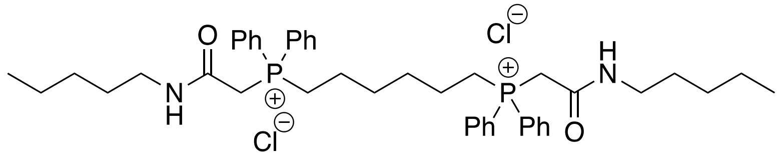

To a solution of 1,6-bis(diphenylphosphino)hexane (0.354 g, 1.00 mmol) in acetonitrile (5 mL) was added 2-chloro-N-pentylacetamide (0.283 g, 2.10 mmol). The solution was flushed with argon, heated to reflux, and stirred for 24 hours. After cooling to room temperature, the excess solvent was removed from the flask using rotary evaporation. The resulting solid was triturated with cyclohexane (~15 mL) at 45 °C for 1 hour and then isolated by vacuum filtration, resulting in **P6P-8A,8A** as a white, powdery solid (0.238 g, 38.9%); ^{1}H NMR (400 MHz, chloroform-*d*), δ 9.33 (t, *J* = 5.3 Hz, 2H), 8.01-7.96 (m, 8H), 7.75-7.71 (m, 4H), 7.66-7.61 (m, 8H), 4.70 (d, *J* = 14.1 Hz, 4H), 3.18 (m, 4H), 3.02 (q, *J* = 6.88 Hz, 4H), 1.62 (s, 8H), 1.33-1.11 (m, 12H), 0.80 (t, *J* = 7.0 Hz, 6H); $^{13}C\{^{1}H\}$ NMR (100.6 MHz, chloroform-*d*), δ 162.4 (d, *J* = 5.3 Hz), 134.8 (d, *J* = 2.9 Hz), 133.5 (d, *J* = 10.1 Hz), 130.2 (d, *J* = 12.5 Hz), 118.4 (d, *J* = 84.8 Hz), 40.1, 30.0 (d, *J* = 52.0 Hz), 29.1, 28.9 (d, *J* = 16.9 Hz), 28.8, 22.7 (d, *J* = 51.1 Hz), 22.3, 21.2, 14.1; $^{31}P\{^{1}H\}$ NMR (162.0 MHz, chloroform-*d*) δ 25.53. HRMS (ESI+): 355.2070, $C_{44}H_{60}N_2O_2P_2$ $[M\text{-}2Cl]^{2+}$ requires 355.2060.

Preparation of P6P-9A,9A

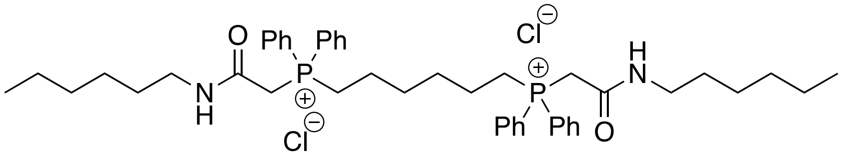

To a solution of 1,6-bis(diphenylphosphino)hexane (0.356 g, 1.00 mmol) in acetonitrile (5 mL) was added 2-chloro-N-hexylacetamide (0.296 g, 2.10 mmol). The solution was flushed with argon, heated to reflux, and stirred for 24.5 hours. After cooling to room temperature, the excess solvent was removed from the flask using rotary evaporation. The resulting solid was triturated with cyclohexane (~15 mL) at 45 ˚C for 1 hour and then isolated by vacuum filtration, resulting in **P6P-9A,9A** as a white, powdery solid (0.584 g, 91.9%); ^{1}H NMR (400 MHz, chloroform-*d*), δ 9.30 (t, *J* = 6.0 Hz, 2H), 8.00-7.96 (m, 8H), 7.74-7.70 (m, 4H), 7.65-7.61 (m, 8H), 4.70 (d, *J* = 14.3 Hz, 4H), 3.17 (m, 4H), 3.01 (q, *J* = 7.0 Hz, 4H), 1.61 (s, 8H), 1.29-1.12 (m, 16H), 0.81 (t, *J* = 7.2 Hz, 6H); ^{13}C{^{1}H} NMR (100.6 MHz, chloroform-*d*), δ 162.7 (d, *J* = 5.3 Hz), 134.7 (d, *J* = 2.9 Hz), 133.5 (d, *J* = 10.1 Hz), 130.2 (d, *J* = 12.5 Hz), 118.4 (d, *J* = 84.8 Hz), 40.1, 31.5, 29.9 (d, *J* = 52.5 Hz), 29.0, 28.7 (d, *J* = 16.9 Hz), 26.7, 22.6, 22.5 (d, *J* = 51.1 Hz), 21.1, 14.1; ^{31}P{^{1}H} NMR (162.0 MHz, chloroform-*d*) δ 25.54. HRMS (ESI+): 369.2236, $C_{46}H_{64}N_2O_2P_2$ $[M-2Cl]^{2+}$ requires 369.2216.

Preparation of P6P-10A,10A

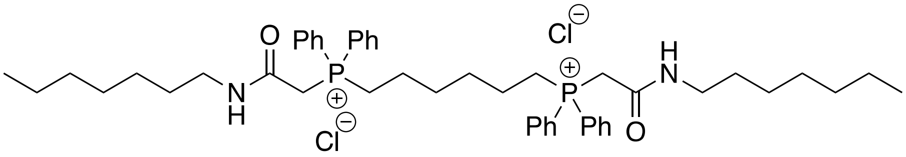

To a solution of 1,6-bis(diphenylphosphino)hexane (0.370 g, 1.00 mmol) in acetonitrile (5 mL) was added 2-chloro-N-heptylacetamide (0.329 g, 2.10 mmol). The solution was flushed with argon, heated to reflux, and stirred for 24.5 hours. After cooling to room temperature, the excess solvent was removed from the flask using rotary evaporation. The resulting solid was triturated with cyclohexane (~15 mL) at 45 °C for 1 hour and then isolated by vacuum filtration, resulting in **P6P-10A,10A** as a white, powdery solid (0.322 g, 47.2%); ^{1}H NMR (400 MHz, chloroform-*d*), δ 9.33 (t, *J* = 5.4 Hz, 2H), 7.99-7.95 (m, 8H), 7.73-7.69 (m, 4H), 7.64-7.60 (m, 8H), 4.69 (d, *J* = 14.3 Hz, 4H), 3.16 (m, 4H), 3.00 (q, *J* = 6.2 Hz, 4H), 1.61 (s, 8H), 1.29-1.15 (m, 20H), 0.83 (t, *J* = 6.9 Hz, 6H); ^{13}C{^{1}H} NMR (100.6 MHz, chloroform-*d*), δ 162.7 (d, *J* = 5.3 Hz), 134.7 (d, *J* = 2.9 Hz), 133.5 (d, *J* = 10.1 Hz), 130.1 (d, *J* = 13.0 Hz), 118.4 (d, *J* = 84.8 Hz), 40.1, 31.8, 29.9 (d, *J* = 53.0 Hz), 29.01, 28.95, 28.7 (d, *J* = 16.9 Hz), 26.9, 22.6 (d, *J* = 51.1 Hz), 22.7, 21.1, 14.2; ^{31}P{^{1}H} NMR (162.0 MHz, chloroform-*d*) δ 25.53. HRMS (ESI+): 383.2388, $C_{48}H_{68}N_2O_2P_2$ [M-2Cl]$^{2+}$ requires 383.2373.

Preparation of P6P-11A,11A

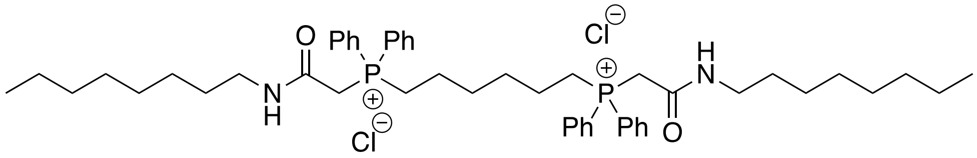

To a solution of 1,6-bis(diphenylphosphino)hexane (0.366 g, 1.00 mmol) in acetonitrile (5 mL) was added 2-chloro-N-octylacetamide (0.345 g, 2.10 mmol). The solution was flushed with argon, heated to reflux, and stirred for 24 hours. After cooling to room temperature, the excess solvent was removed from the flask using rotary evaporation. The resulting solid was triturated with cyclohexane (~15 mL) at 45 °C for 1 hour and then isolated by vacuum filtration, resulting in **P6P-11A,11A** as a white, powdery solid (0.444 g, 66.6%); ^{1}H NMR (400 MHz, chloroform-*d*), δ 9.24 (t, *J* = 5.7 Hz, 2H), 8.01-7.96 (m, 8H), 7.74-7.71 (m, 4H), 7.65-7.61 (m, 8H), 4.70 (d, *J* = 14.6 Hz, 4H), 3.18 (m, 4H), 3.01 (q, *J* = 6.1 Hz, 4H), 1.62 (s, 8H), 1.28-1.14 (m, 24H), 0.83 (t, *J* = 7.0 Hz, 6H); ^{13}C{^{1}H} NMR (100.6 MHz, chloroform-*d*), δ 162.8 (d, *J* = 5.3 Hz), 134.7 (d, *J* = 2.9 Hz), 133.5 (d, *J* = 10.1 Hz), 130.1 (d, *J* = 13.0 Hz), 118.4 (d, *J* = 84.8 Hz), 40.2, 31.9, 29.9 (d, *J* = 52.5 Hz), 29.27, 29.23, 29.1, 28.7 (d, *J* = 16.9 Hz), 27.0, 22.6 (d, *J* = 51.1 Hz), 22.7, 21.1, 14.2; ^{31}P{^{1}H} NMR (162.0 MHz, chloroform-*d*) δ 25.61. HRMS (ESI+): 397.2538, $C_{50}H_{72}N_2O_2P_2$ [M-2Cl]$^{2+}$ requires 397.2529.

Preparation of P6P-12A,12A

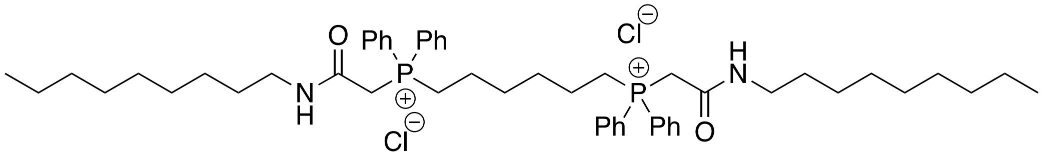

To a solution of 1,6-bis(diphenylphosphino)hexane (0.355 g, 1.00 mmol) in acetonitrile (5 mL) was added 2-chloro-N-nonylacetamide (0.393 g, 2.10 mmol). The solution was flushed with argon, heated to reflux, and stirred for 21 hours. After cooling to room temperature, the excess solvent was removed from the flask using rotary evaporation. The resulting solid was triturated with cyclohexane (~15 mL) at 45 °C for 1 hour and then isolated by vacuum filtration, resulting in **P6P-12A,12A** as an orange, flaky solid (0.684 g, 97.9%); ^{1}H NMR (400 MHz, chloroform-*d*), δ 9.24 (t, J = 6.0 Hz, 2H), 8.00-7.94 (m, 8H), 7.73-7.69 (m, 4H), 7.64-7.59 (m, 8H), 4.69 (d, J = 14.3 Hz, 4H), 3.16 (m, 4H), 3.00 (q, J = 6.2 Hz, 4H), 1.60 (s, 8H), 1.28-1.13 (m, 28H), 0.84 (t, J = 6.9 Hz, 6H); ^{13}C{^{1}H} NMR (100.6 MHz, chloroform-*d*), δ 162.8 (d, J = 5.3 Hz), 134.7 (d, J = 2.9 Hz), 133.5 (d, J = 10.1 Hz), 130.1 (d, J = 13.0 Hz), 118.4 (d, J = 85.3 Hz), 40.2, 31.9, 29.9 (d, J = 52.5 Hz), 29.5, 29.34, 29.31, 29.1, 28.7 (d, J = 16.9 Hz), 27.0, 22.6 (d, J = 51.1 Hz), 22.7, 21.1, 14.2; ^{31}P{^{1}H} NMR (162.0 MHz, chloroform-*d*) δ 25.58. HRMS (ESI+): 411.2694, $C_{52}H_{76}N_2O_2P_2$ [M-2Cl]$^{2+}$ requires 411.2686.

Preparation of P6P-13A,13A

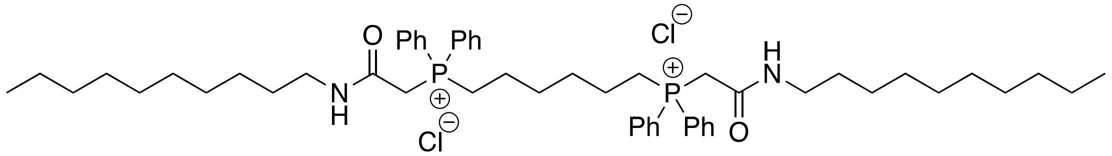

To a solution of 1,6-bis(diphenylphosphino)hexane (0.369 g, 1.00 mmol) in acetonitrile (5 mL) was added 2-chloro-N-decylacetamide (0.391 g, 2.10 mmol). The solution was flushed with argon, heated to reflux, and stirred for 20 hours. After cooling to room temperature, the excess solvent was removed from the flask using rotary evaporation. The resulting solid was triturated with cyclohexane (~15 mL) at 45 °C for 1 hour and then isolated by vacuum filtration, resulting in **P6P-13A,13A** as a white, powdery solid (0.496 g, 66.3%); ^{1}H NMR (400 MHz, chloroform-*d*), δ 9.31 (t, *J* = 5.6 Hz, 2H), 8.02-7.96 (m, 8H), 7.74-7.70 (m, 4H), 7.65-7.61 (m, 8H), 4.70 (d, *J* = 14.4 Hz, 4H), 3.17 (m, 4H), 3.01 (q, *J* = 6.3 Hz, 4H), 1.62 (s, 8H), 1.27-1.15 (m, 32H), 0.85 (t, *J* = 6.9 Hz, 6H); ^{13}C{^{1}H} NMR (100.6 MHz, chloroform-*d*), δ 162.8 (d, *J* = 5.3 Hz), 134.7 (d, *J* = 2.9 Hz), 133.5 (d, *J* = 10.1 Hz), 130.1 (d, *J* = 12.5 Hz), 118.4 (d, *J* = 84.8 Hz), 40.2, 32.0, 29.9 (d, *J* = 53.0 Hz), 29.64, 29.59, 28.4, 29.3, 29.1, 28.7 (d, *J* = 16.9 Hz), 27.0, 22.6 (d, *J* = 51.1 Hz), 22.8, 21.1, 14.2; ^{31}P{^{1}H} NMR (162.0 MHz, chloroform-*d*) δ 25.55. HRMS (ESI+): 425.2861, $C_{54}H_{80}N_2O_2P_2$ $[M-2Cl]^{2+}$ requires 425.2842.

Preparation of P6P-15A,15A

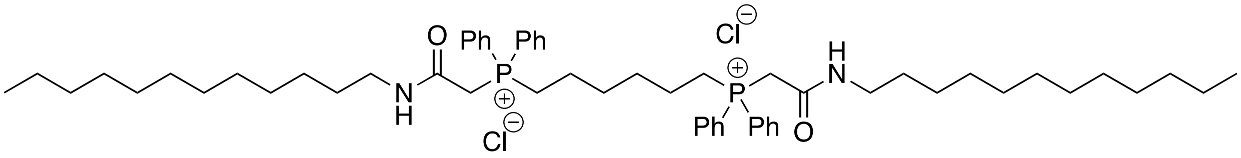

To a solution of 1,6-bis(diphenylphosphino)hexane (0.355 g, 1.00 mmol) in acetonitrile (5 mL) was added 2-chloro-N-dodecylacetamide (0.427 g, 2.10 mmol). The solution was flushed with argon, heated to reflux, and stirred for 24.5 hours. After cooling to room temperature, the excess solvent was removed from the flask using rotary evaporation. The resulting solid was triturated with cyclohexane (~15 mL) at 45 °C for 1 hour and then isolated by vacuum filtration, resulting in **P6P-15A,15A** as a white, powdery solid (0.659 g, 86.2%); ^{1}H NMR (400 MHz, chloroform-*d*), δ 9.31 (t, J = 5.4 Hz, 2H), 8.02-7.96 (m, 8H), 7.74-7.70 (m, 4H), 7.65-7.61 (m, 8H), 4.70 (d, J = 14.4 Hz, 4H), 3.18 (m, 4H), 3.01 (q, J = 6.2 Hz, 4H), 1.62 (m, 8H), 1.27-1.14 (m, 40H), 0.85 (t, J = 6.9 Hz, 6H); ^{13}C{^{1}H} NMR (100.6 MHz, chloroform-*d*), δ 162.8 (d, J = 5.3 Hz), 134.7 (d, J = 3.4 Hz), 133.5 (d, J = 10.1 Hz), 130.1 (d, J = 12.5 Hz), 118.4 (d, J = 85.3 Hz), 40.2, 32.0, 29.9 (d, J = 52.5 Hz), 29.73, 29.71, 29.70, 29.6, 29.4, 29.3, 29.1, 28.7 (d, J = 16.9 Hz), 27.0, 22.6 (d, J = 51.1 Hz), 22.8, 21.1, 14.2; ^{31}P{^{1}H} NMR (162.0 MHz, chloroform-*d*) δ 25.56. HRMS (ESI+): 453.3169, $C_{58}H_{88}N_2O_2P_2$ [M-2Cl]$^{2+}$ requires 453.3155.

III. NMR Spectra

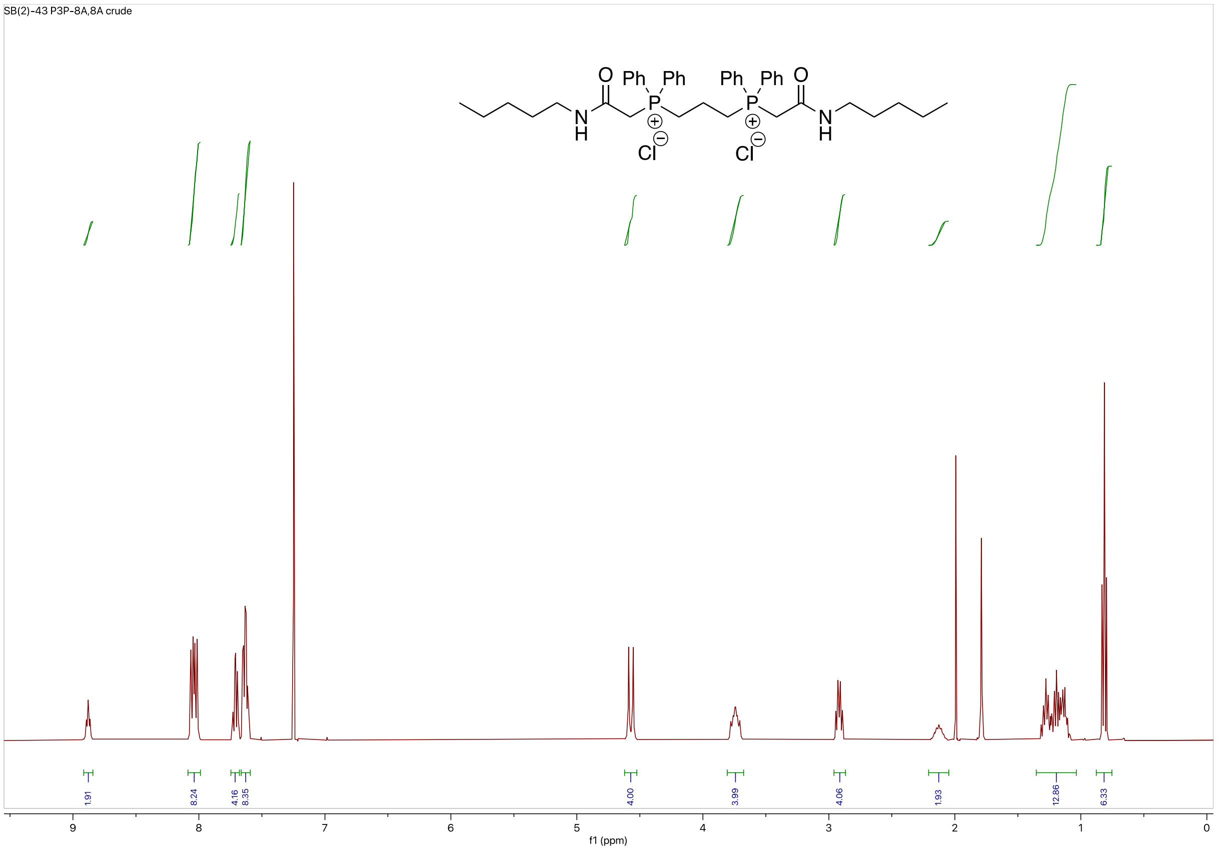

Figure S9a ^{1}H NMR (400 MHz) of **P3P-8A,8A** in $CDCl_3$.

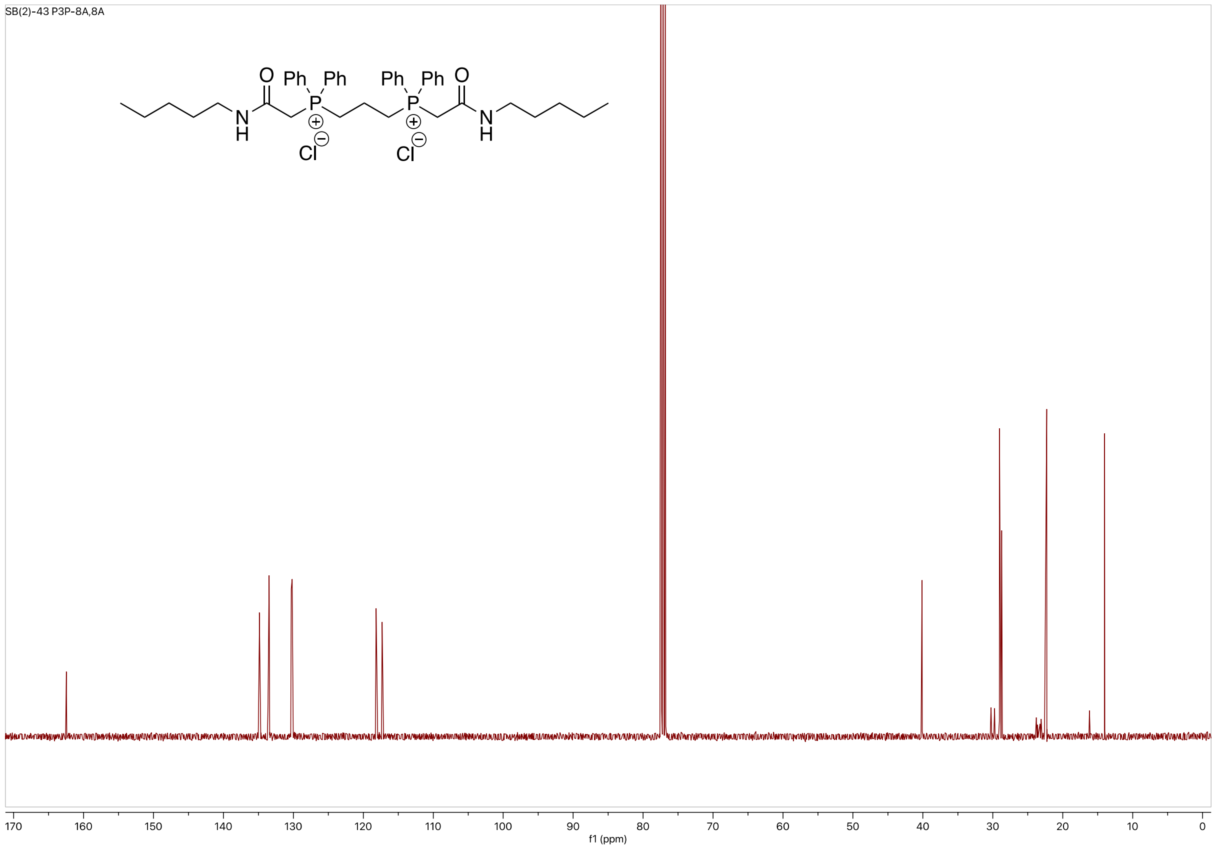

Figure S9b. ^{13}C NMR (100.6 MHz) of **P3P-8A,8A** in $CDCl_3$.

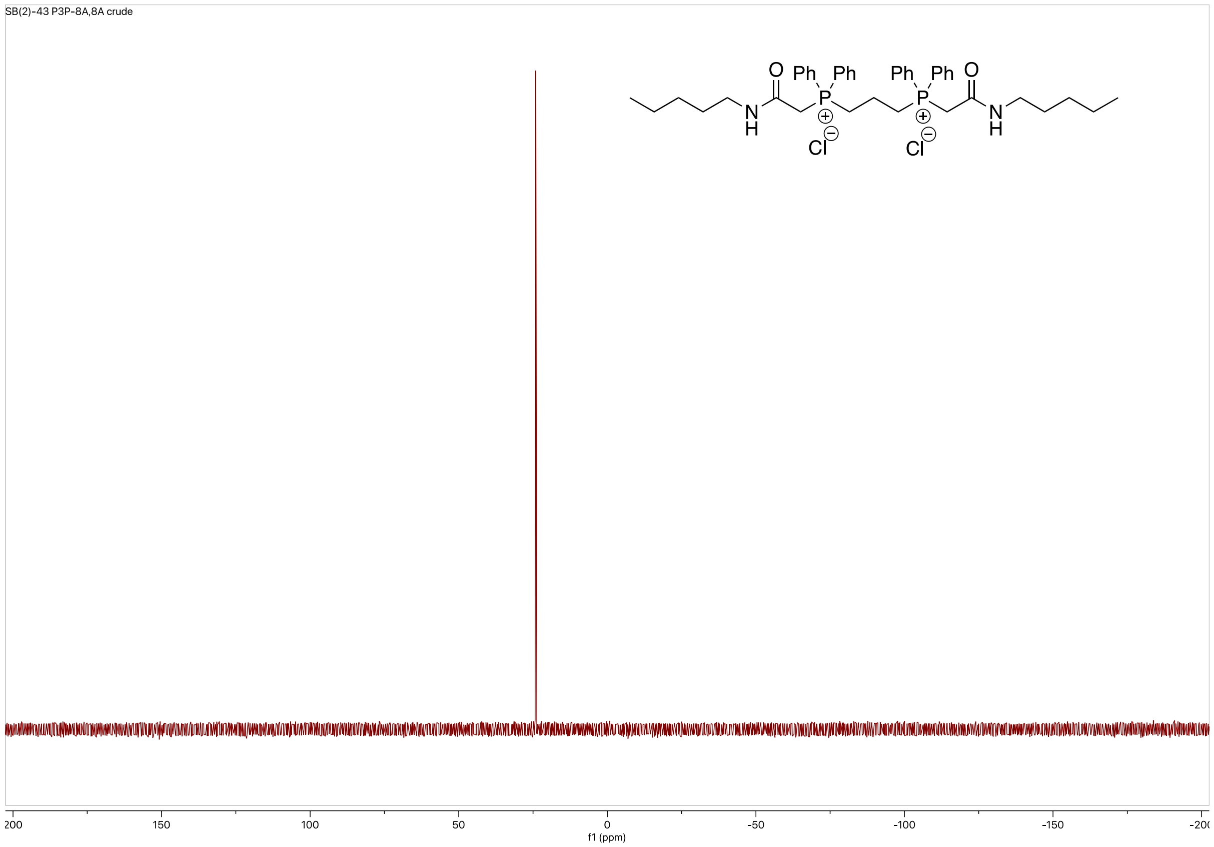

Figure S9c. ^{31}P NMR (162 MHz) of **P3P-8A,8A** in $CDCl_3$.

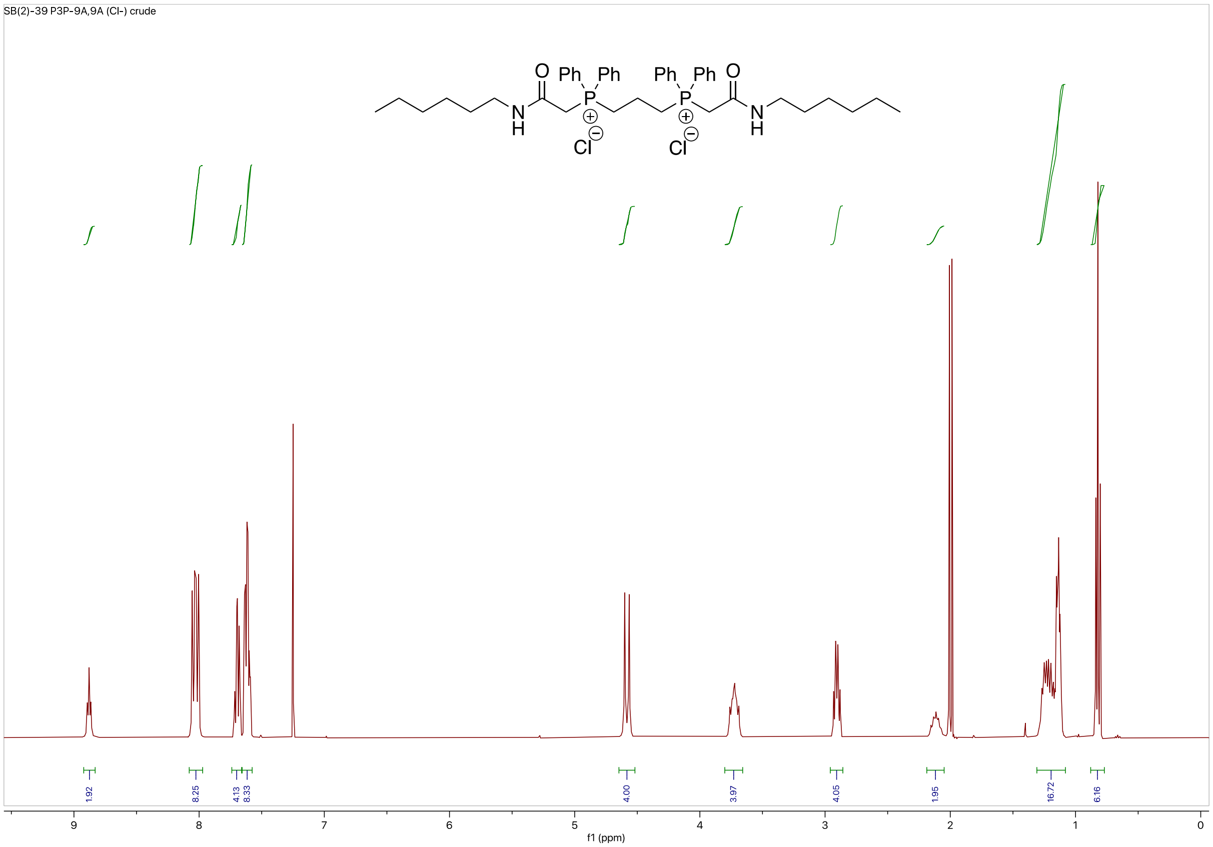

Figure S10a. ^{1}H NMR (400 MHz) of **P3P-9A,9A** in $CDCl_3$.

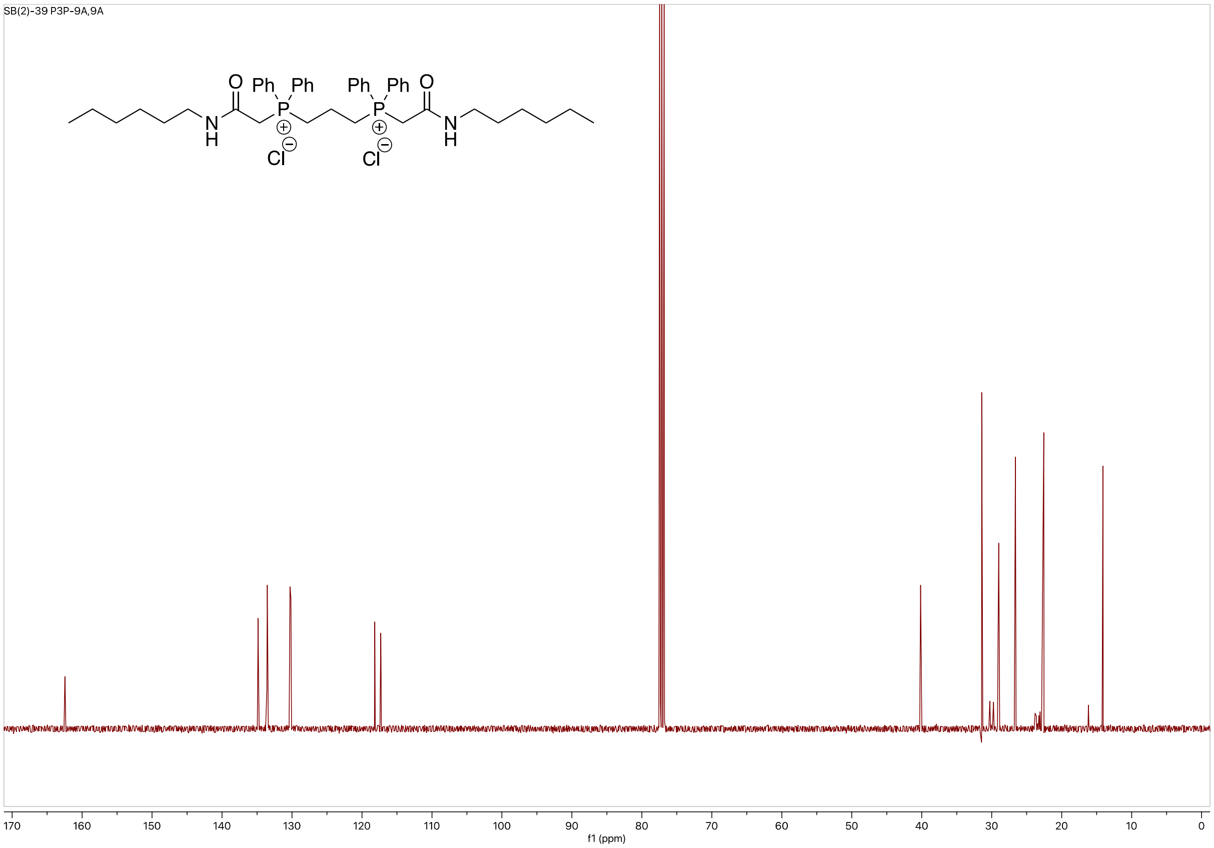

Figure S10b. ^{13}C NMR (100.6 MHz) of **P3P-9A,9A** in $CDCl_3$.

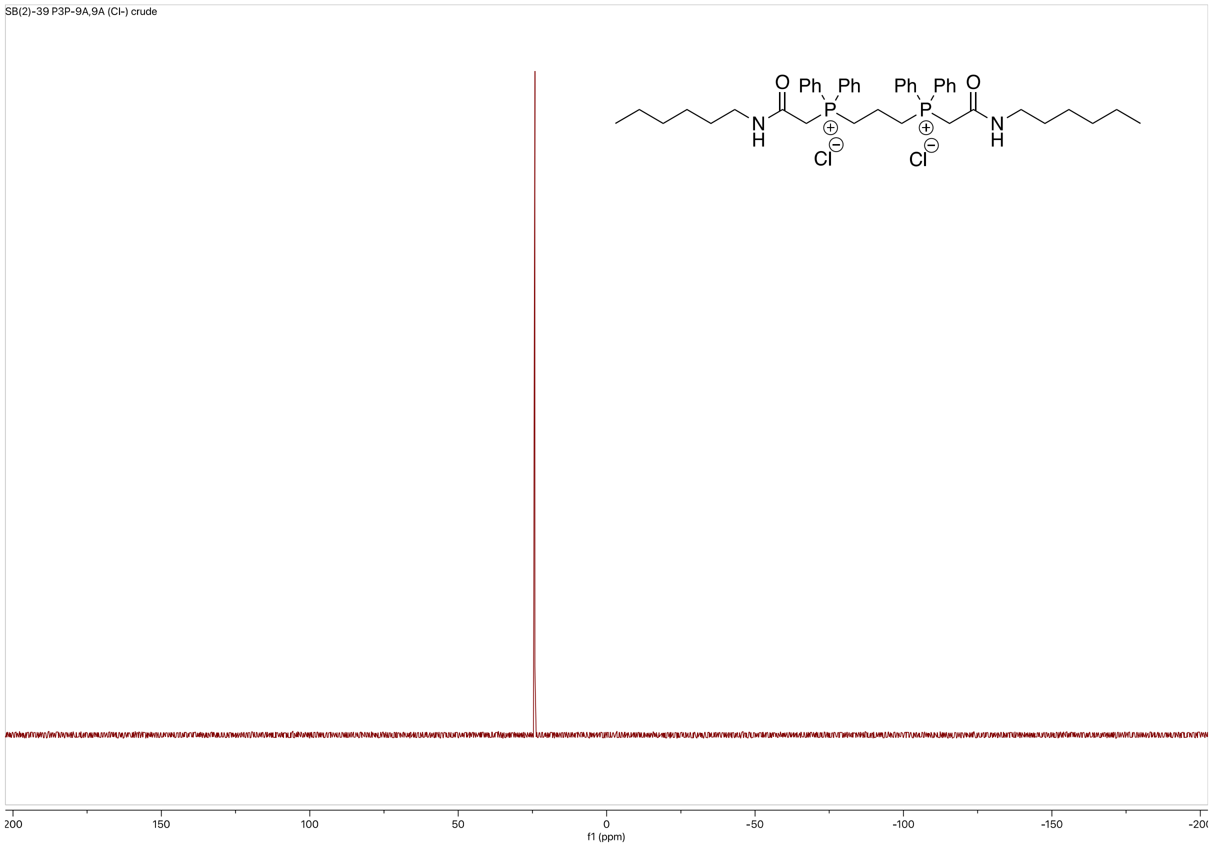

Figure S10c. ^{31}P NMR (162 MHz) of **P3P-9A,9A** in $CDCl_3$.

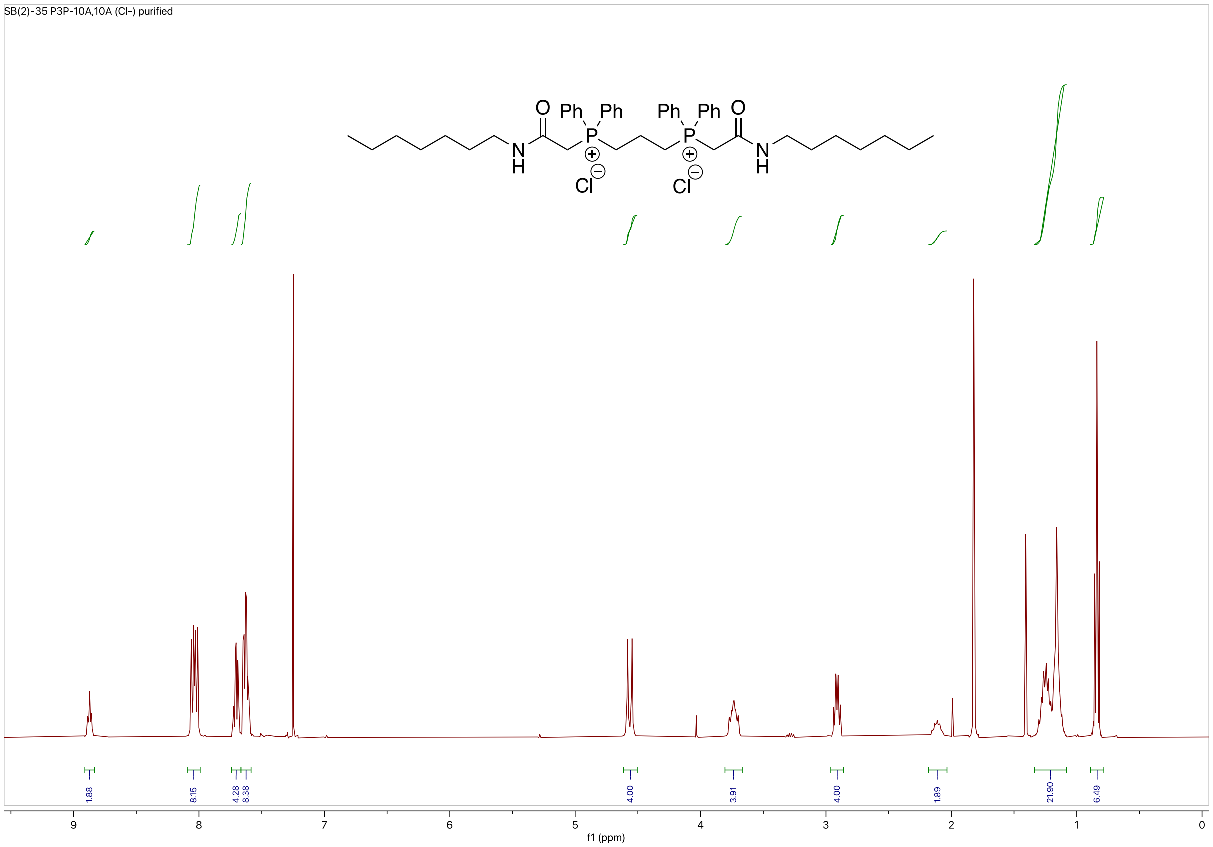

Figure S11a. ^{1}H NMR (400 MHz) of **P3P-10A,10A** in $CDCl_3$.

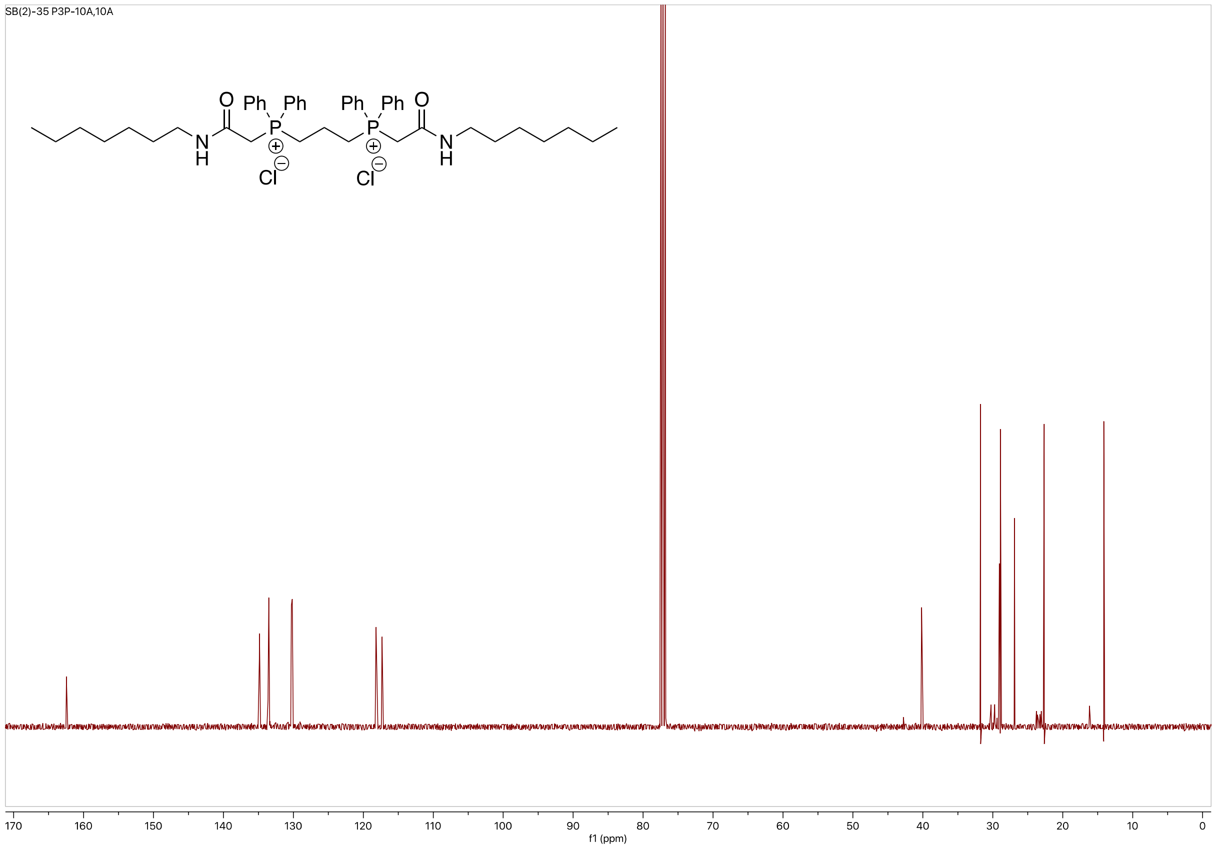

Figure S11b. ^{13}C NMR (100.6 MHz) of **P3P-10A,10A** in $CDCl_3$.

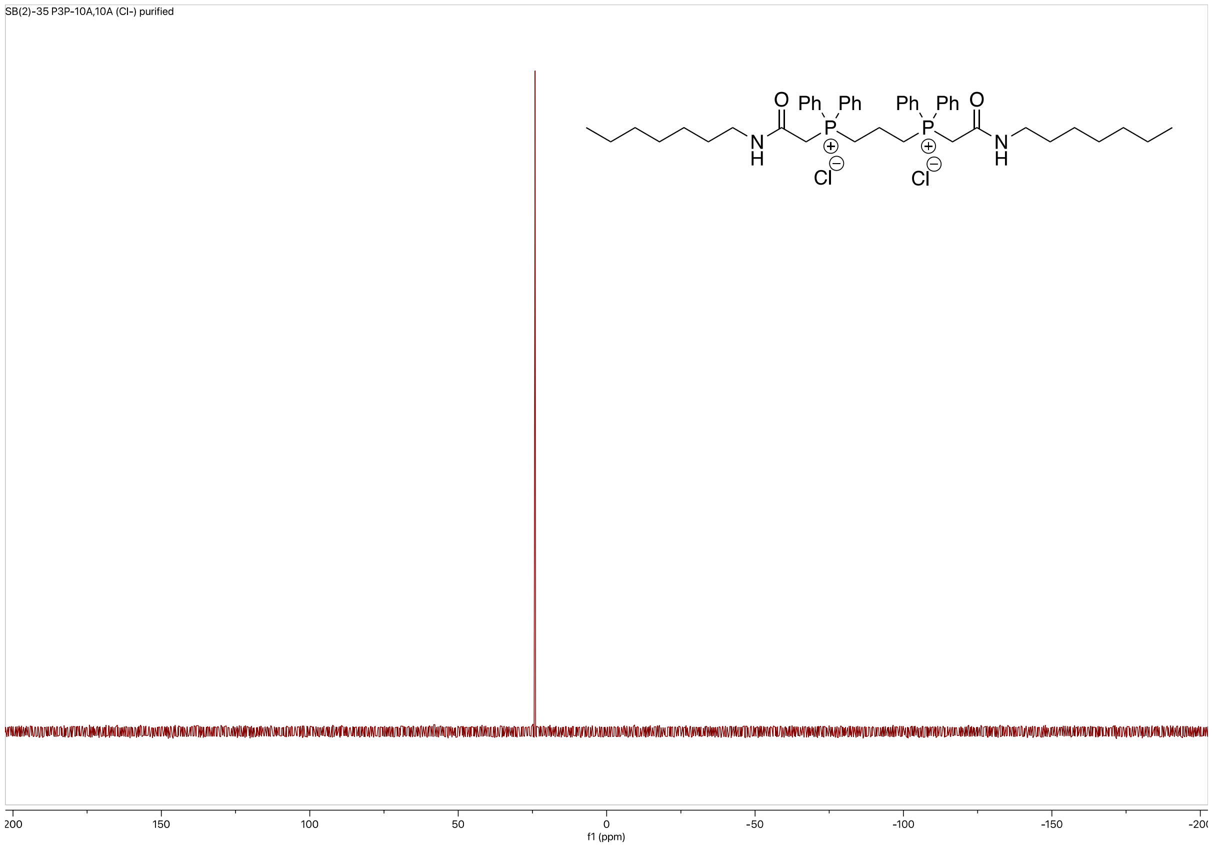

Figure S11c. ^{31}P NMR (162 MHz) of **P3P-10A,10A** in $CDCl_3$.

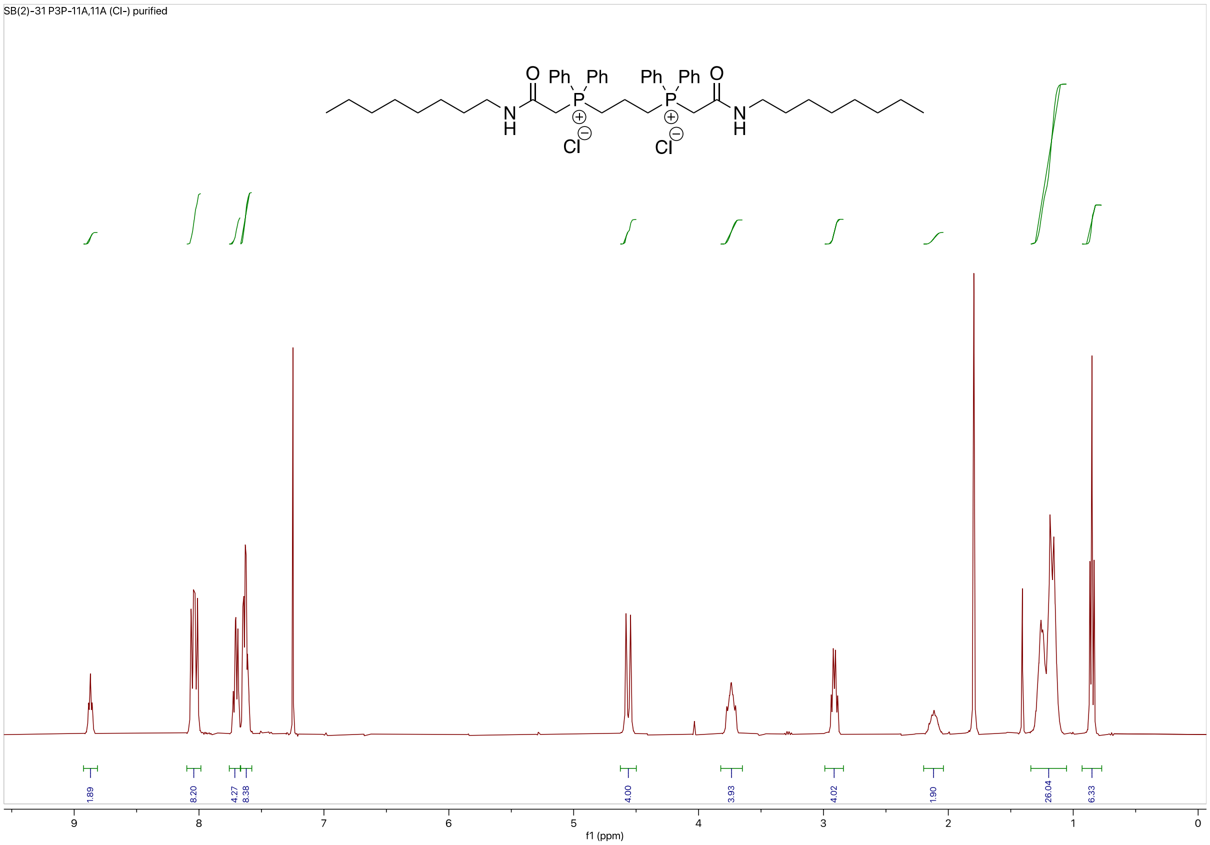

Figure S12a. ^{1}H NMR (400 MHz) of **P3P-11A,11A** in $CDCl_3$.

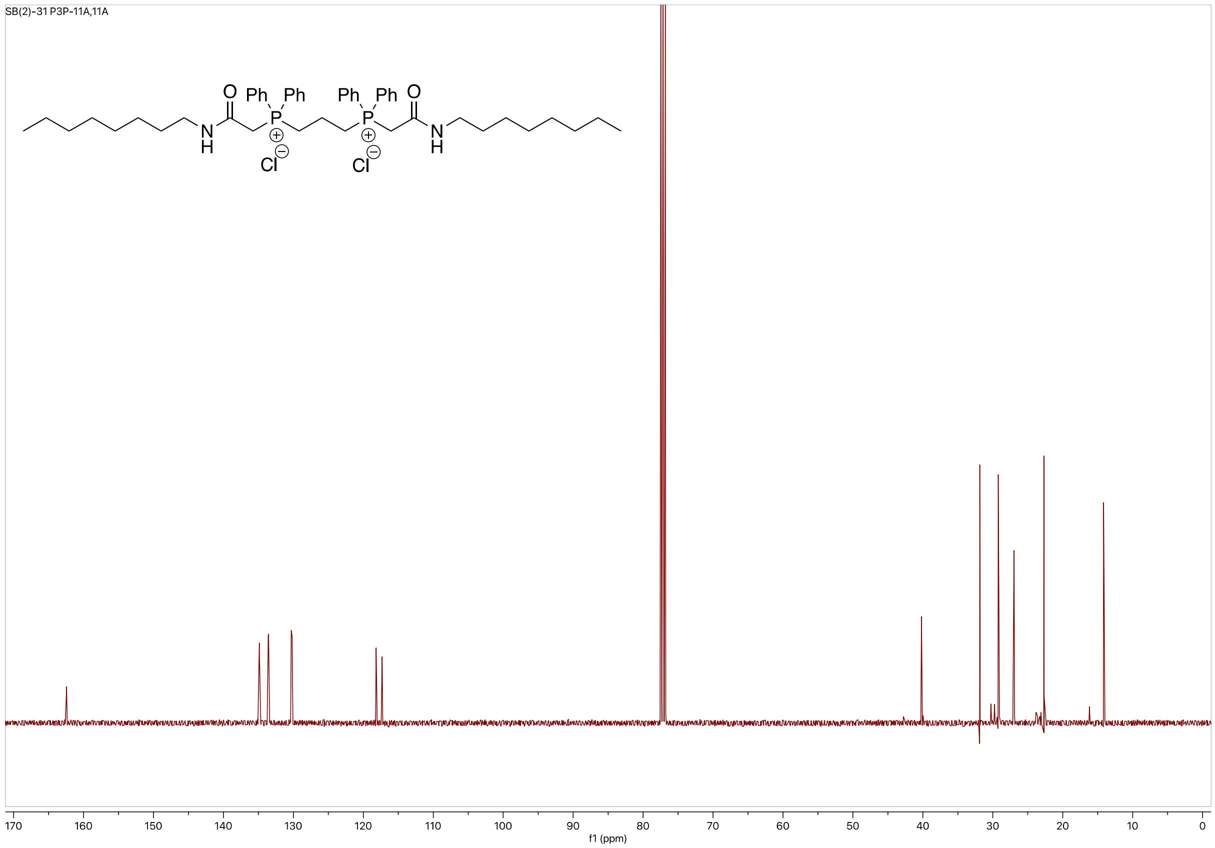

Figure S12b. ^{13}C NMR (100.6 MHz) of **P3P-11A,11A** in $CDCl_3$.

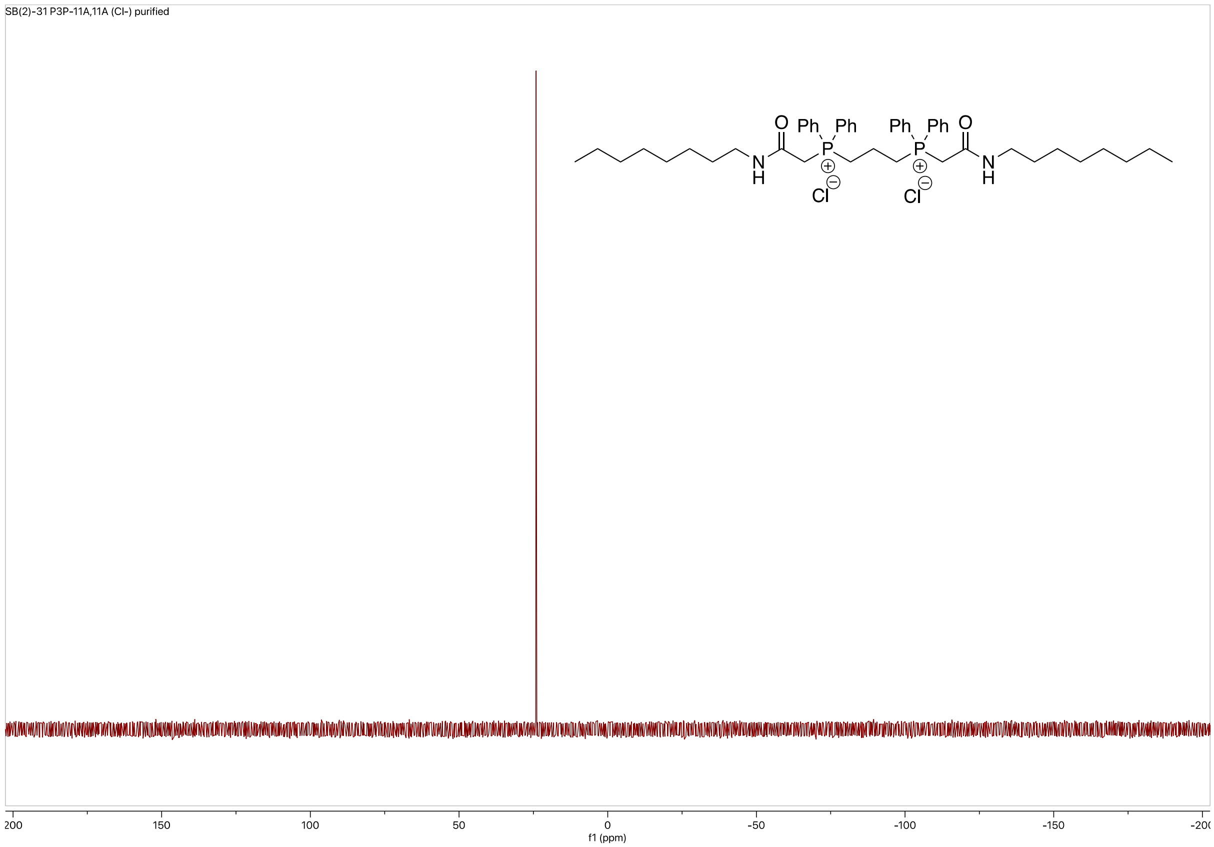

Figure S12b. ^{31}P NMR (162 MHz) of **P3P-11A,11A** in $CDCl_3$.

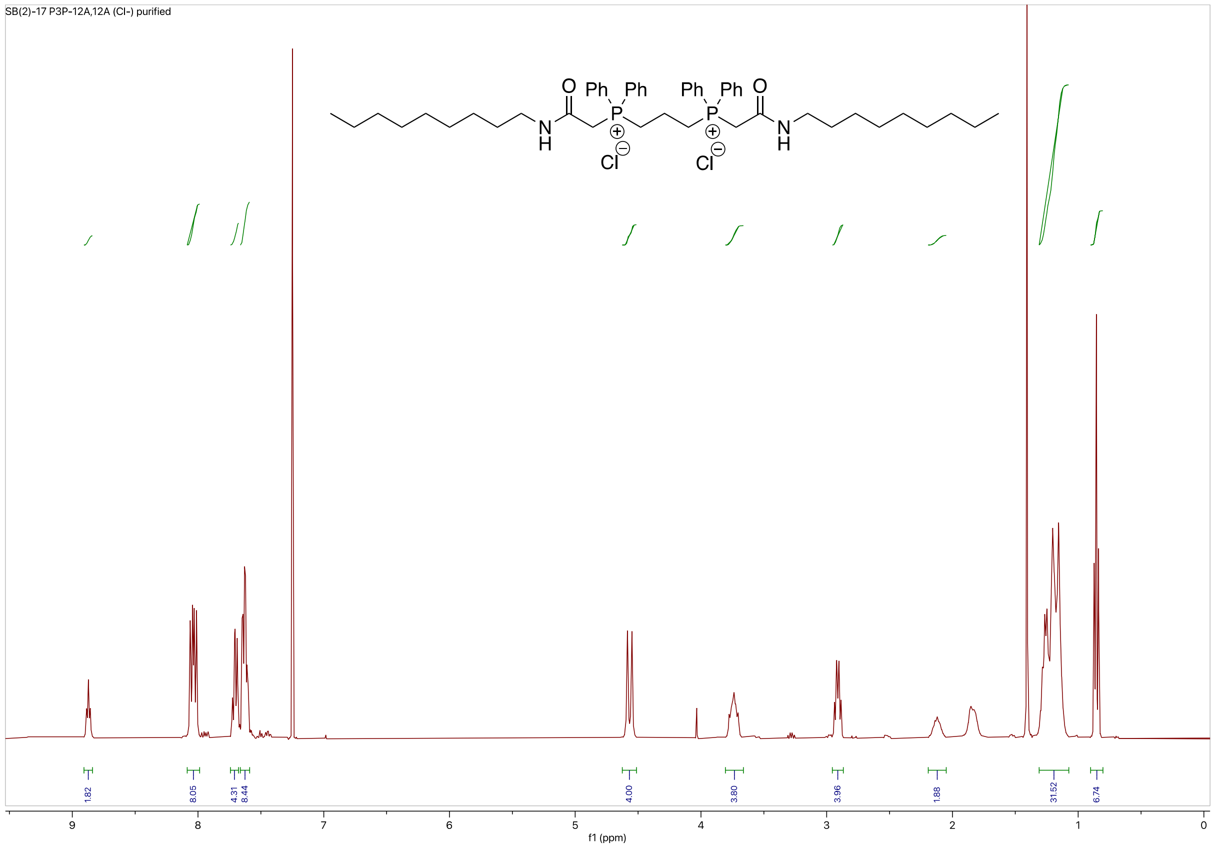

Figure S13a. ^{1}H NMR (400 MHz) of **P3P-12A,12A** in $CDCl_3$.

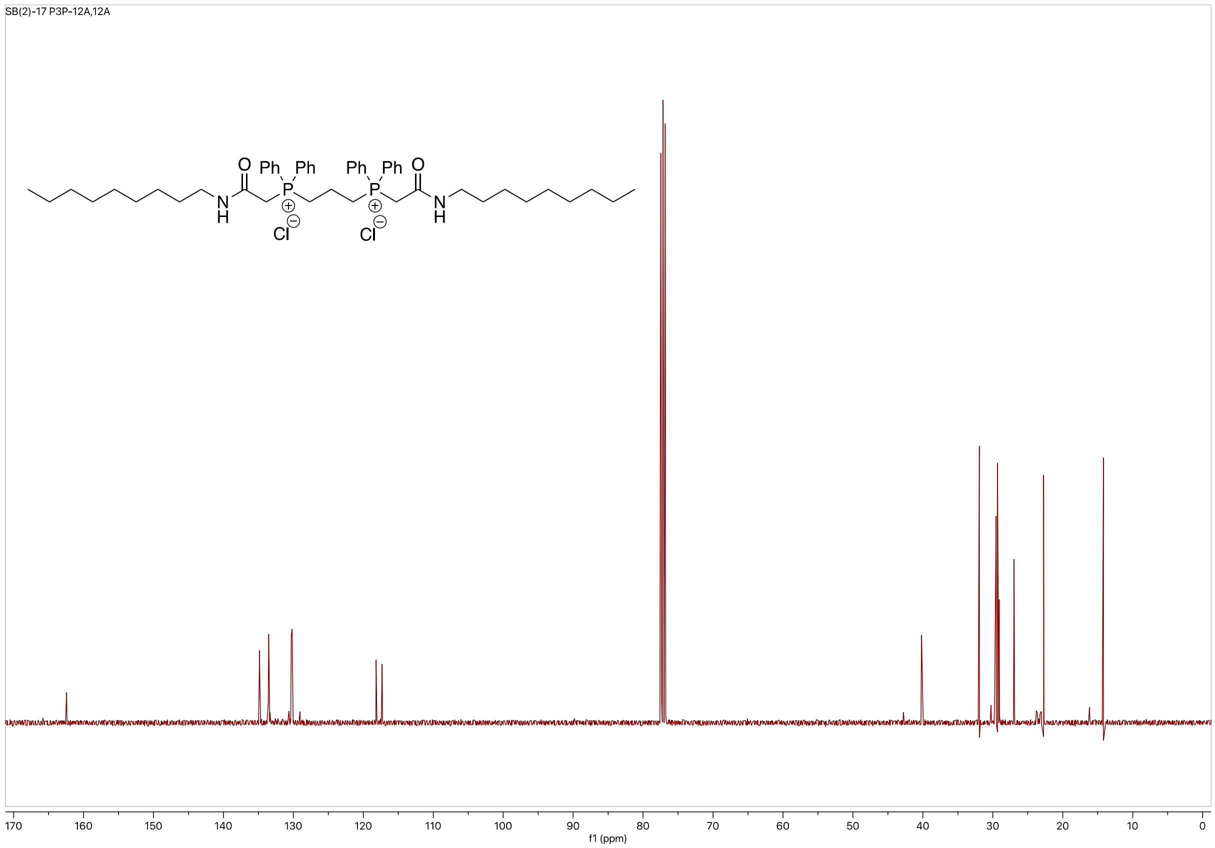

Figure S13b. ^{13}C NMR (100.6 MHz) of **P3P-12A,12A** in $CDCl_3$.

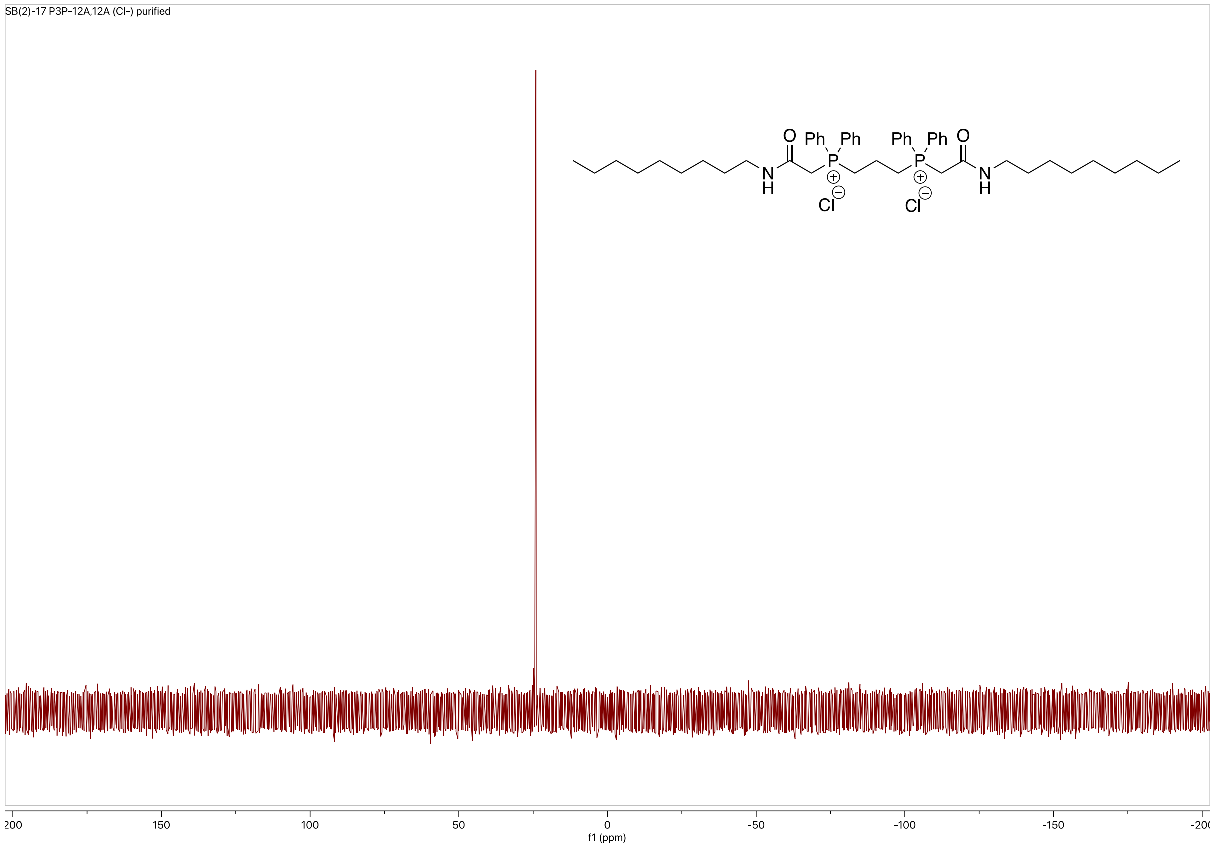

Figure S13c. ^{31}P NMR (162 MHz) of **P3P-12A,12A** in $CDCl_3$.

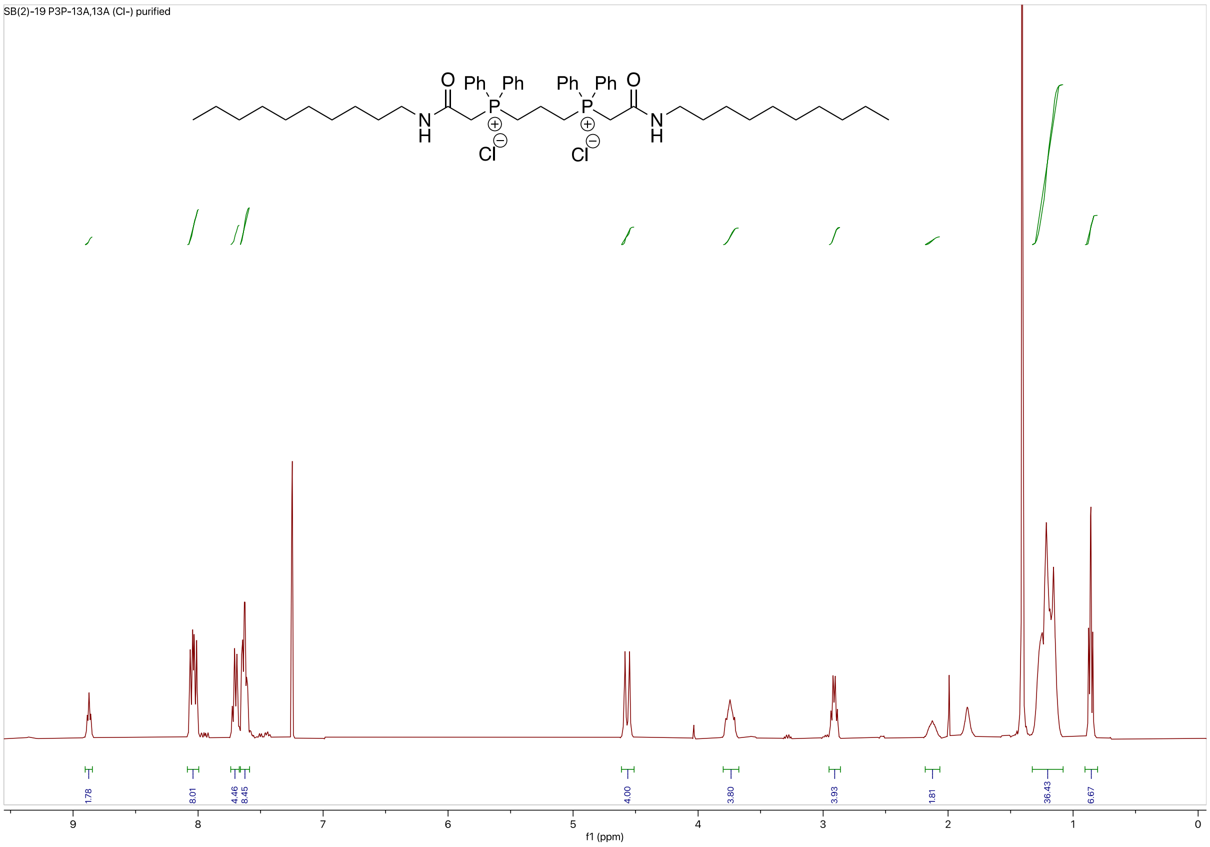

Figure S14a. ^{1}H NMR (400 MHz) of **P3P-13A,13A** in $CDCl_3$.

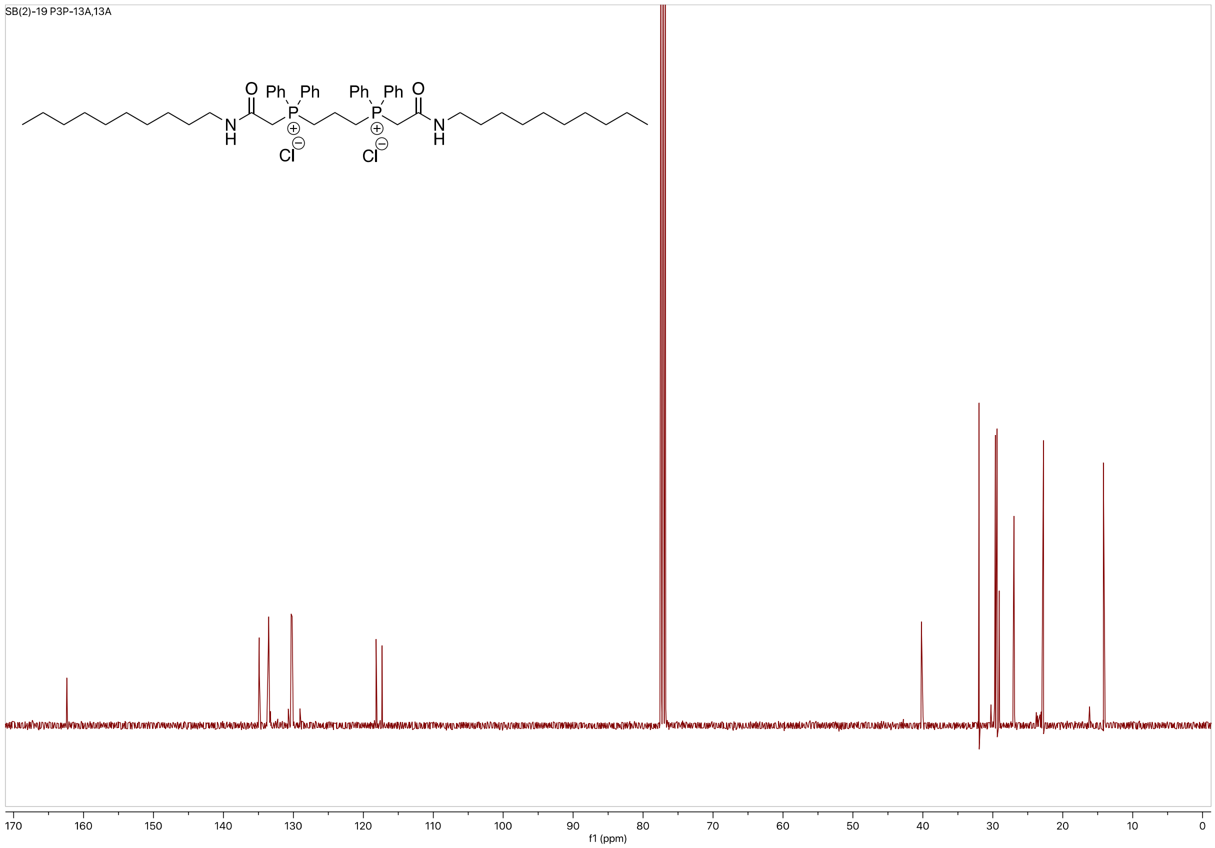

Figure S14b. ^{13}C NMR (100.6 MHz) of **P3P-13A,13A** in $CDCl_3$.

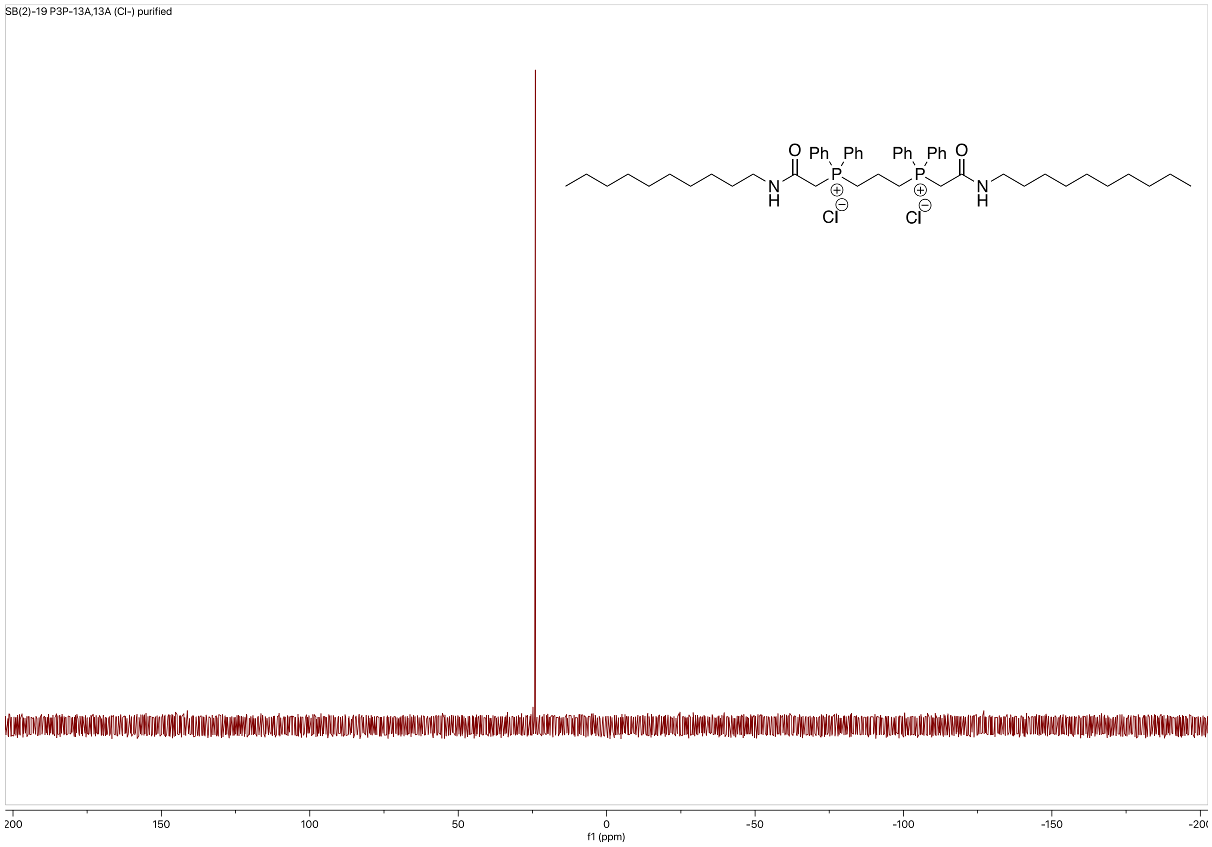

Figure S14c. ^{31}P NMR (162 MHz) of **P3P-13A,13A** in $CDCl_3$.

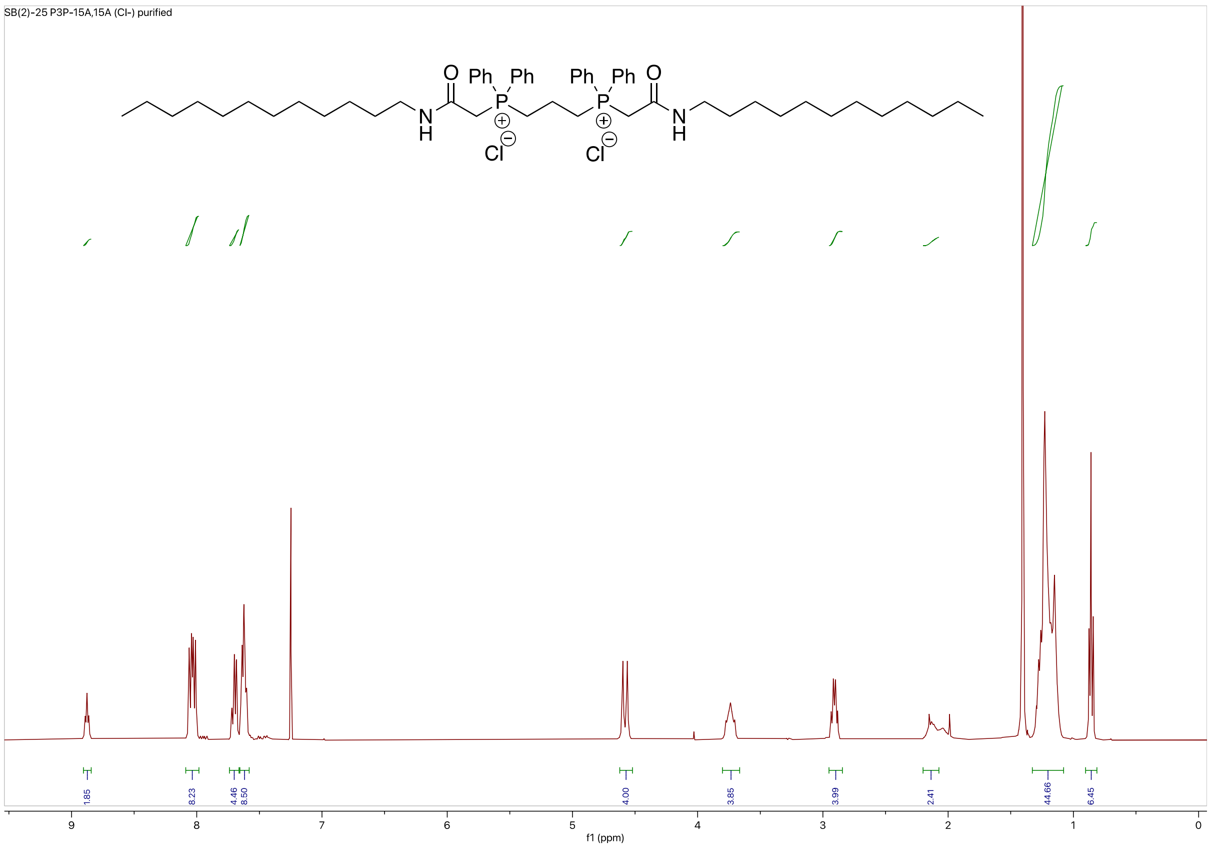

Figure S15a. ^{1}H NMR (400 MHz) of **P3P-15A,15A** in $CDCl_3$.

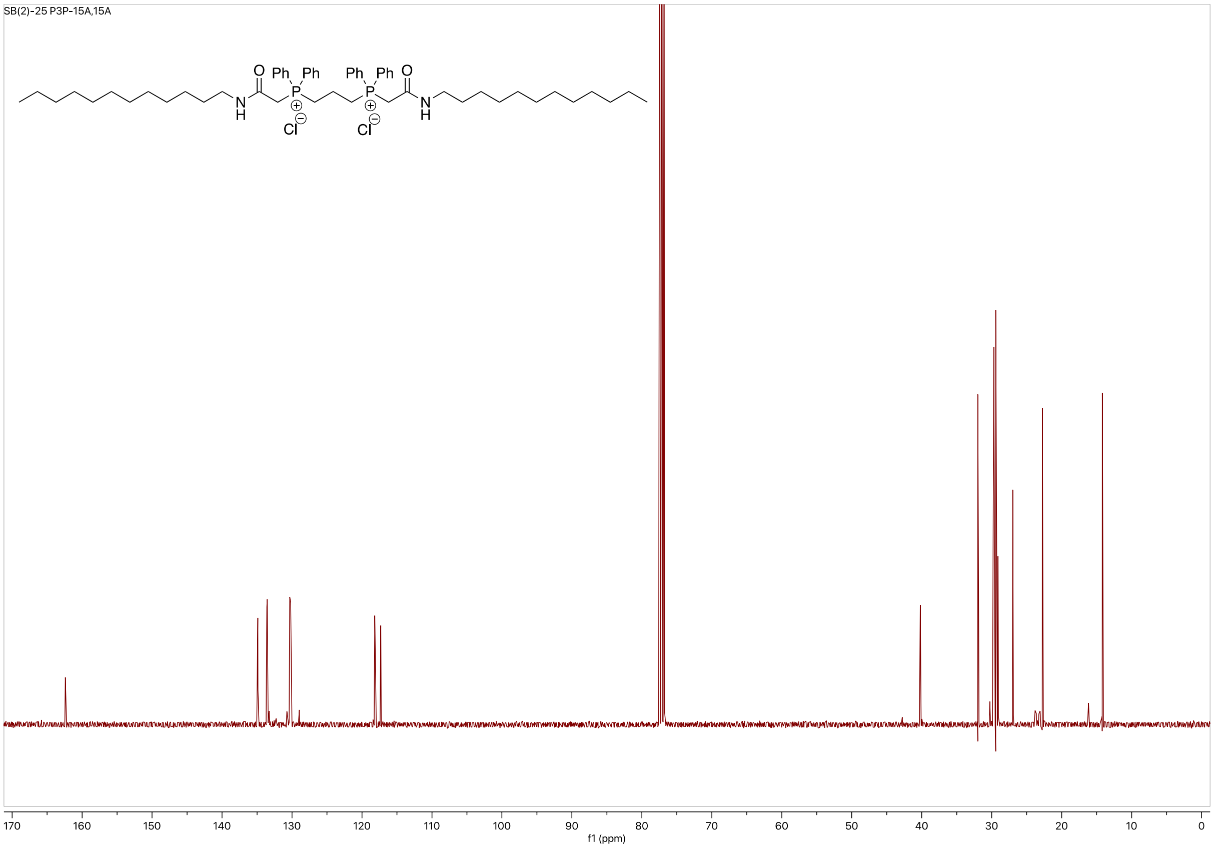

Figure S15b. ^{13}C NMR (100.6 MHz) of **P3P-15A,15A** in $CDCl_3$.

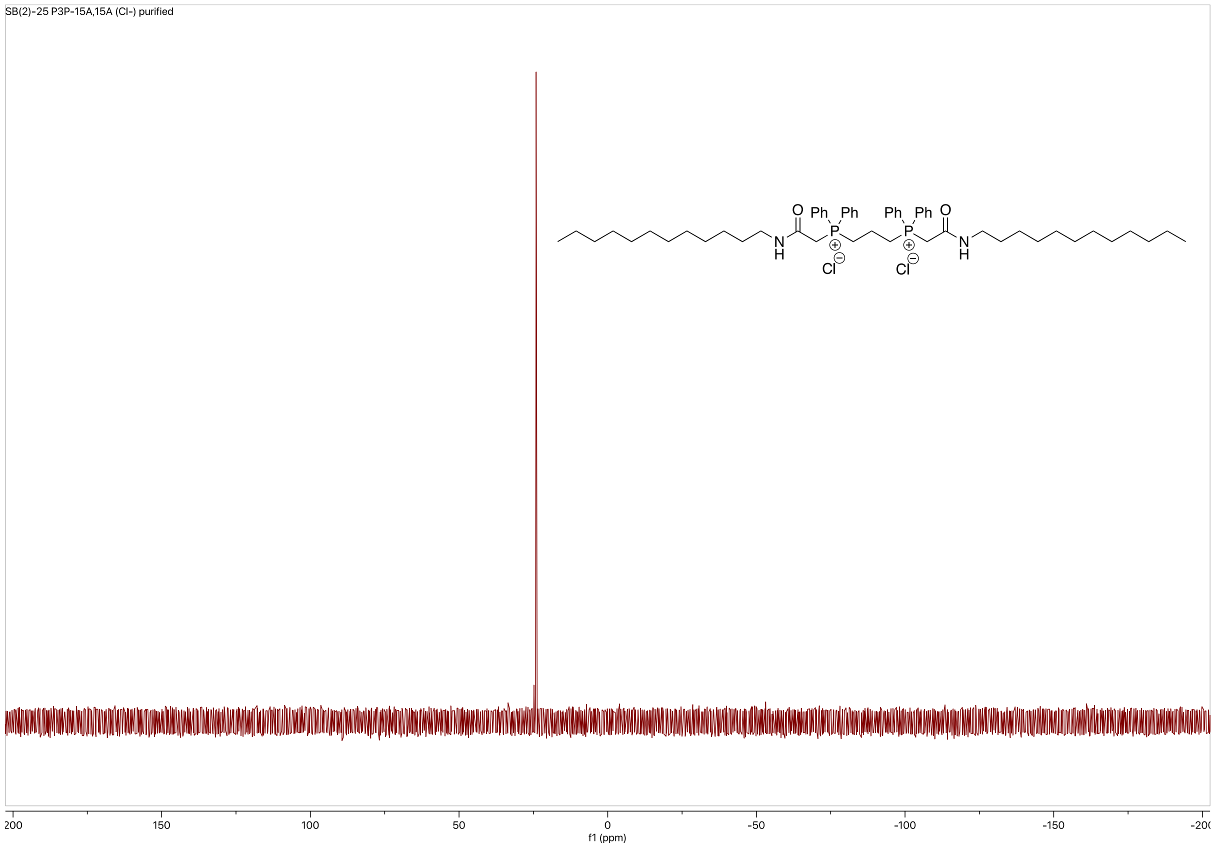

Figure S15c. ^{31}P NMR (162 MHz) of **P3P-15A,15A** in $CDCl_3$.

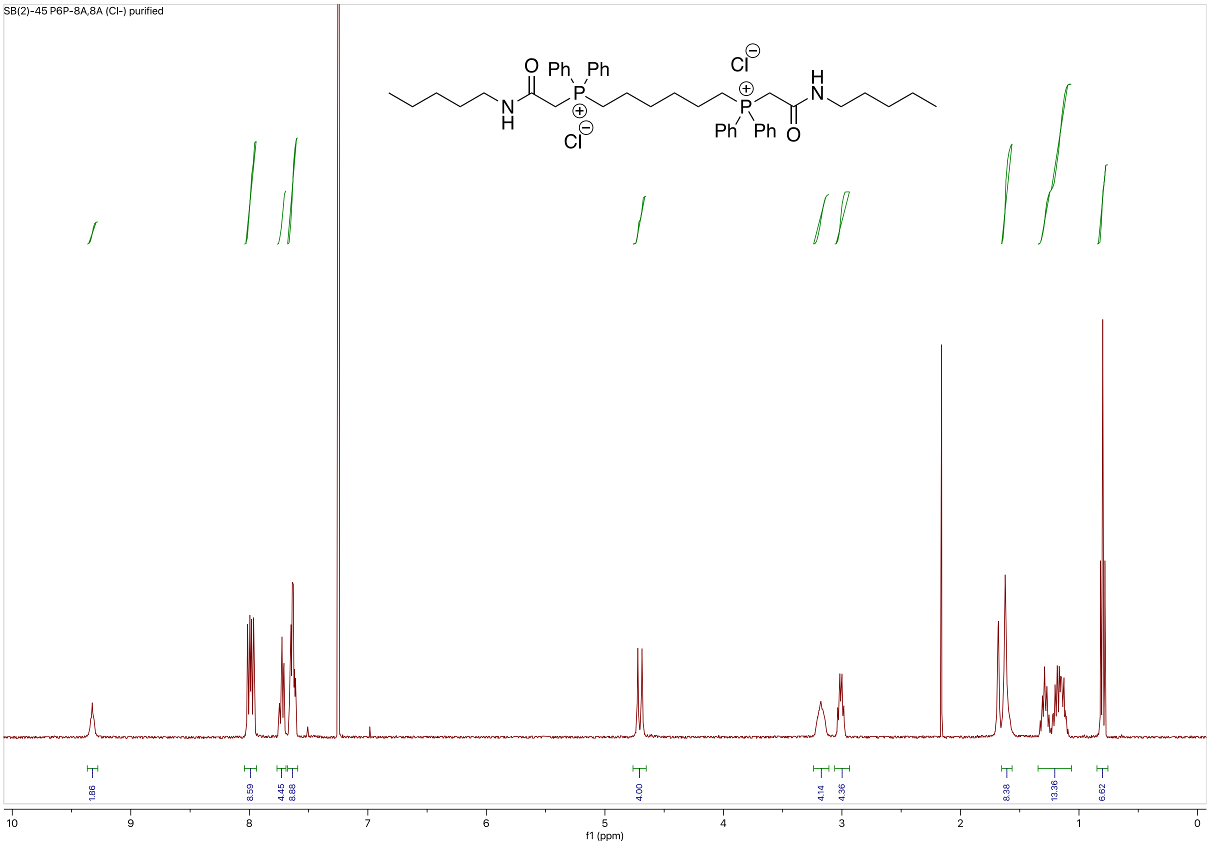

Figure S16a. ^{1}H NMR (400 MHz) of **P6P-8A,8A** in $CDCl_3$.

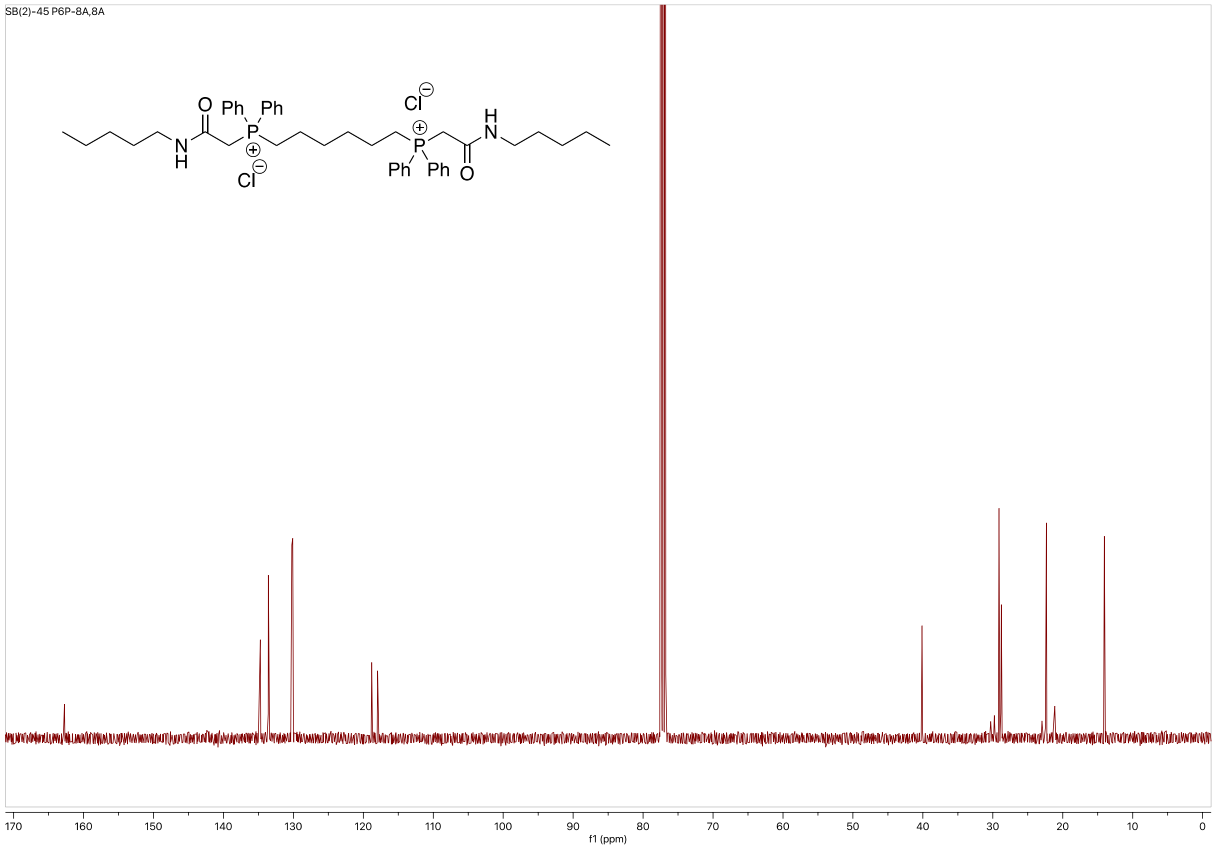

Figure S16b. ^{13}C NMR (100.6 MHz) of **P6P-8A,8A** in $CDCl_3$.

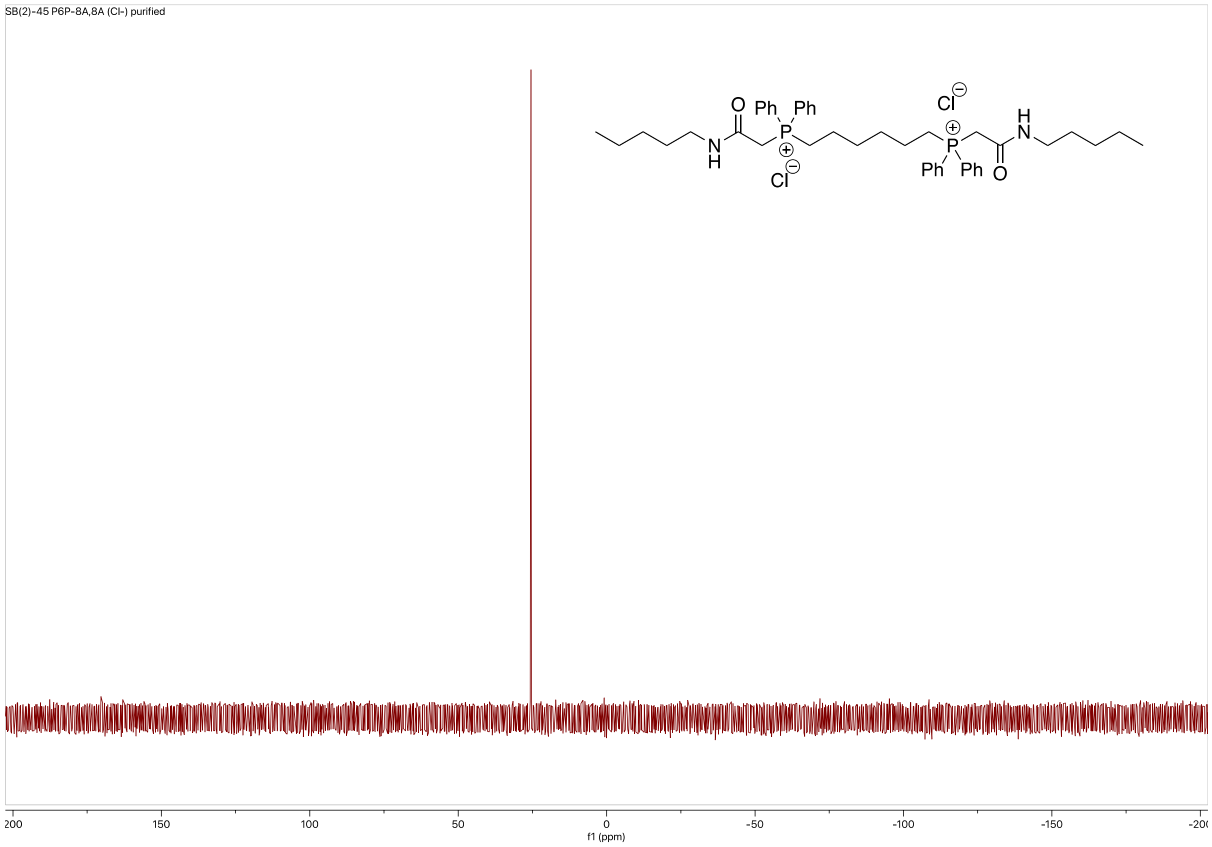

Figure S16c. ^{31}P NMR (162 MHz) of **P6P-8A,8A** in $CDCl_3$.

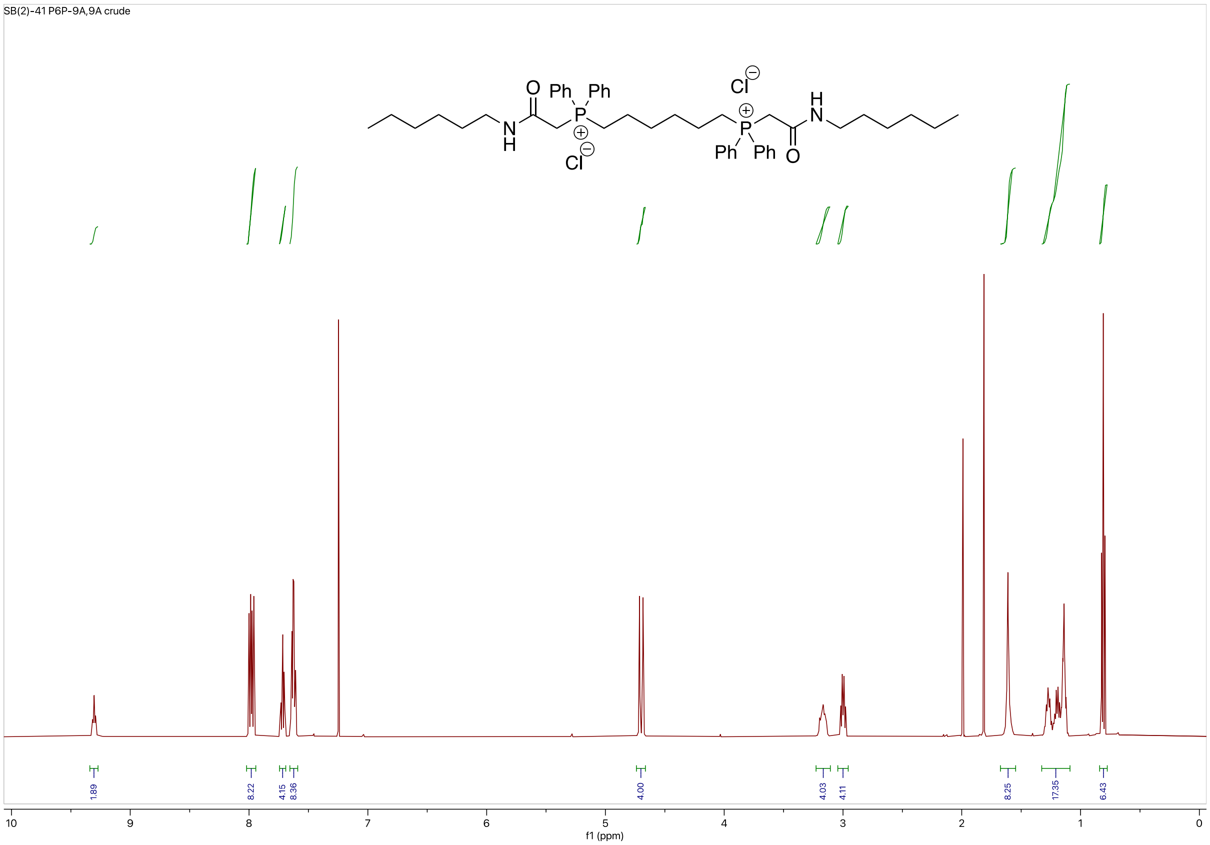

Figure S17a. ^{1}H NMR (400 MHz) of **P6P-9A,9A** in $CDCl_3$.

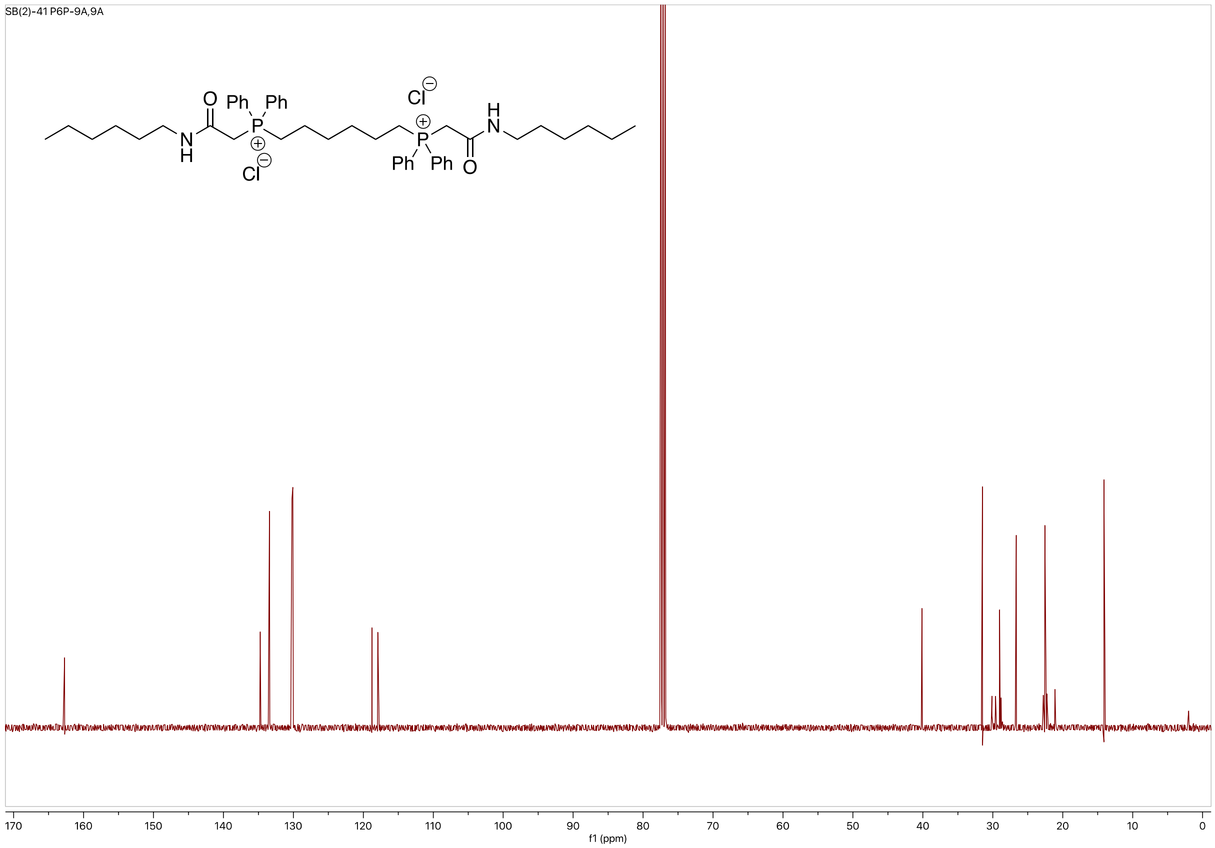

Figure S17b. ^{13}C NMR (100.6 MHz) of **P6P-9A,9A** in $CDCl_3$.

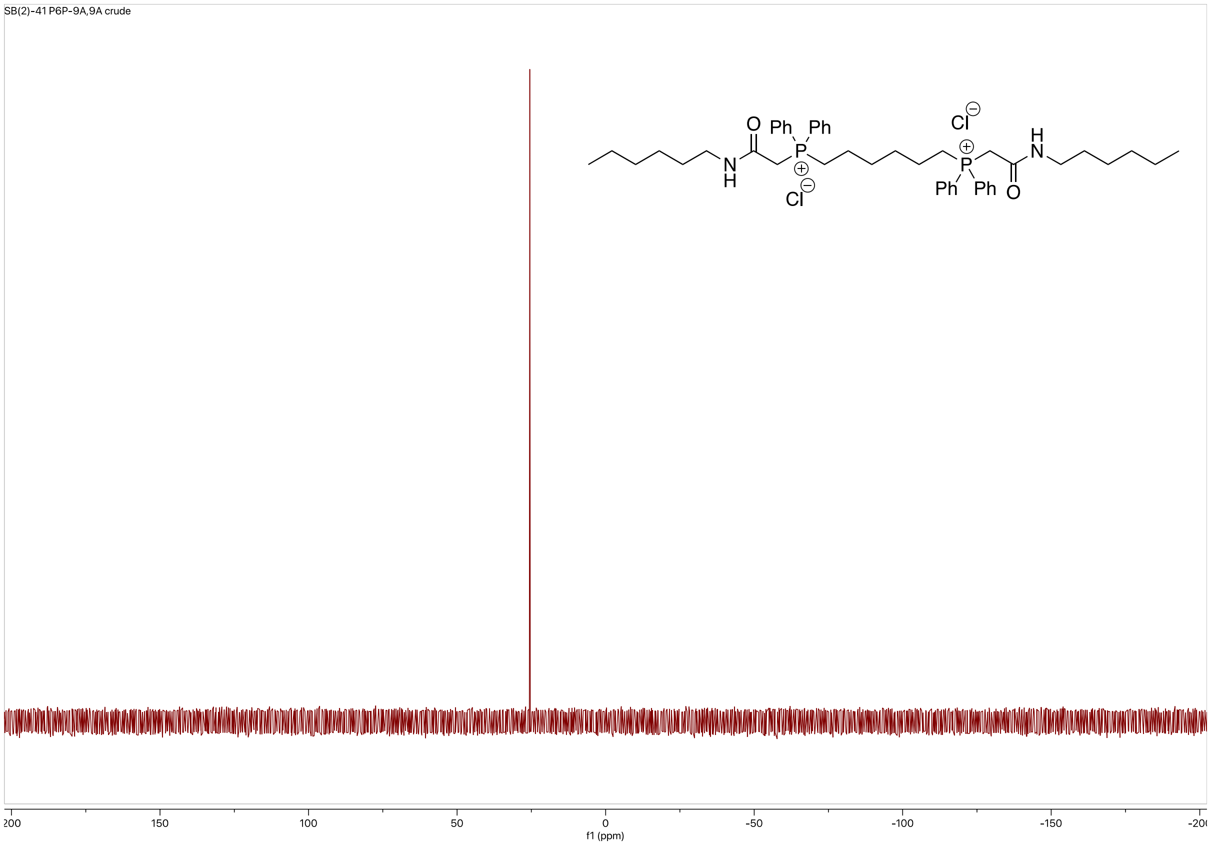

Figure S17c. ^{31}P NMR (162 MHz) of **P6P-9A,9A** in $CDCl_3$.

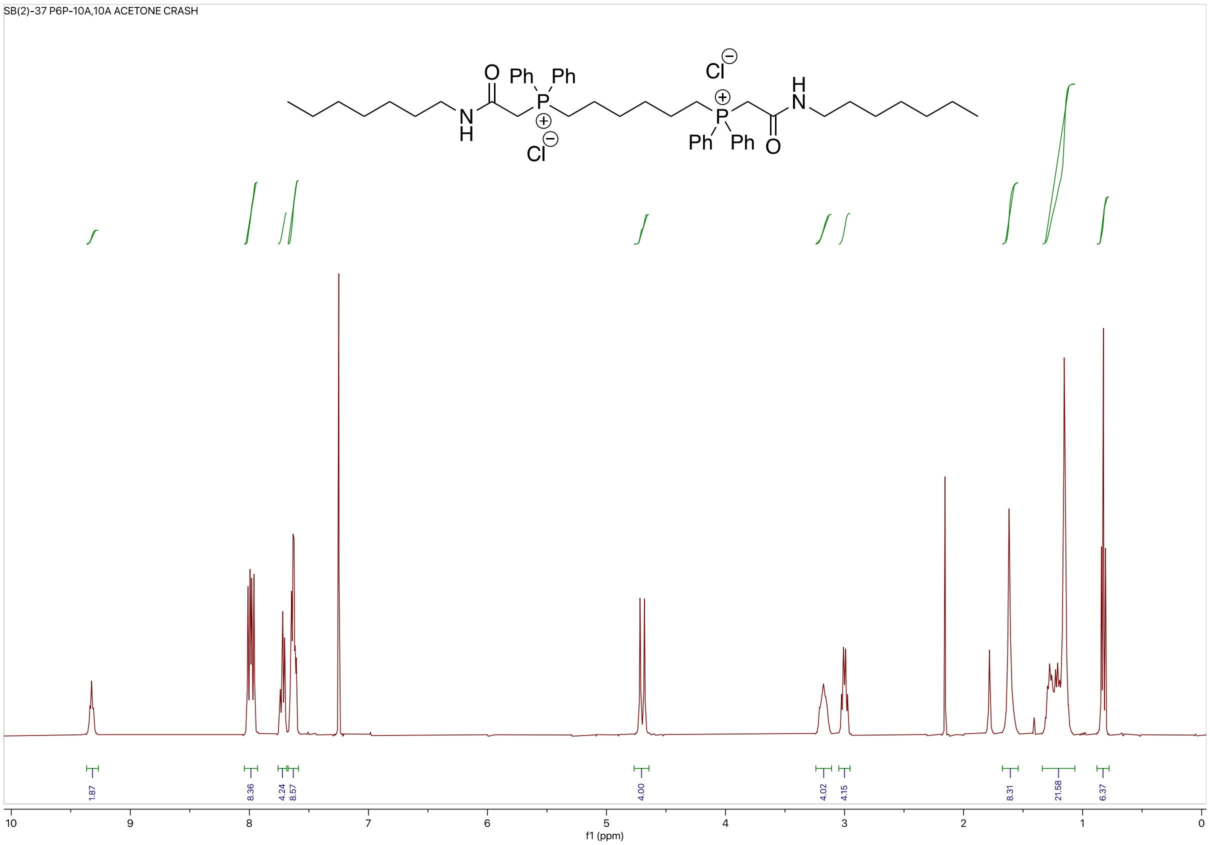

Figure S18a. ^{1}H NMR (400 MHz) of **P6P-10A,10A** in $CDCl_3$.

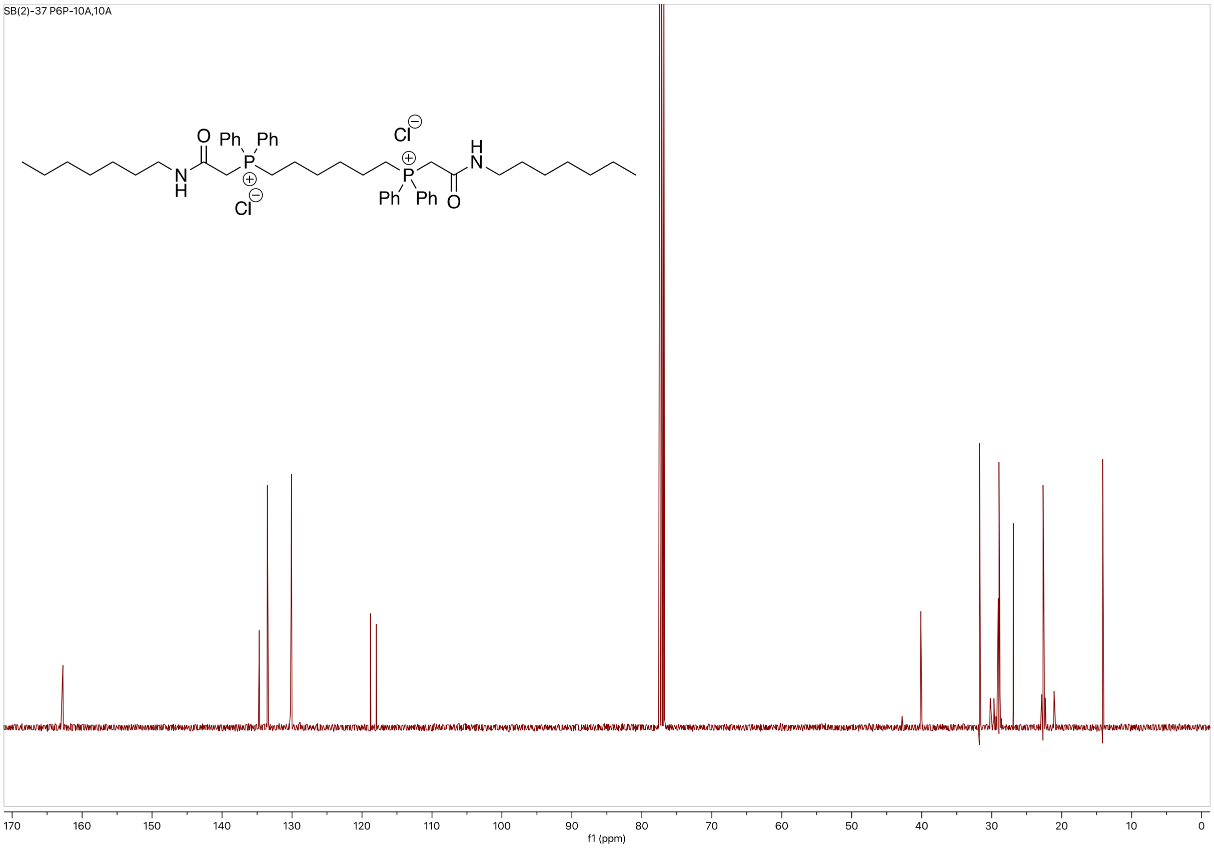

Figure S18b. ^{13}C NMR (100.6 MHz) of **P6P-10A,10A** in $CDCl_3$.

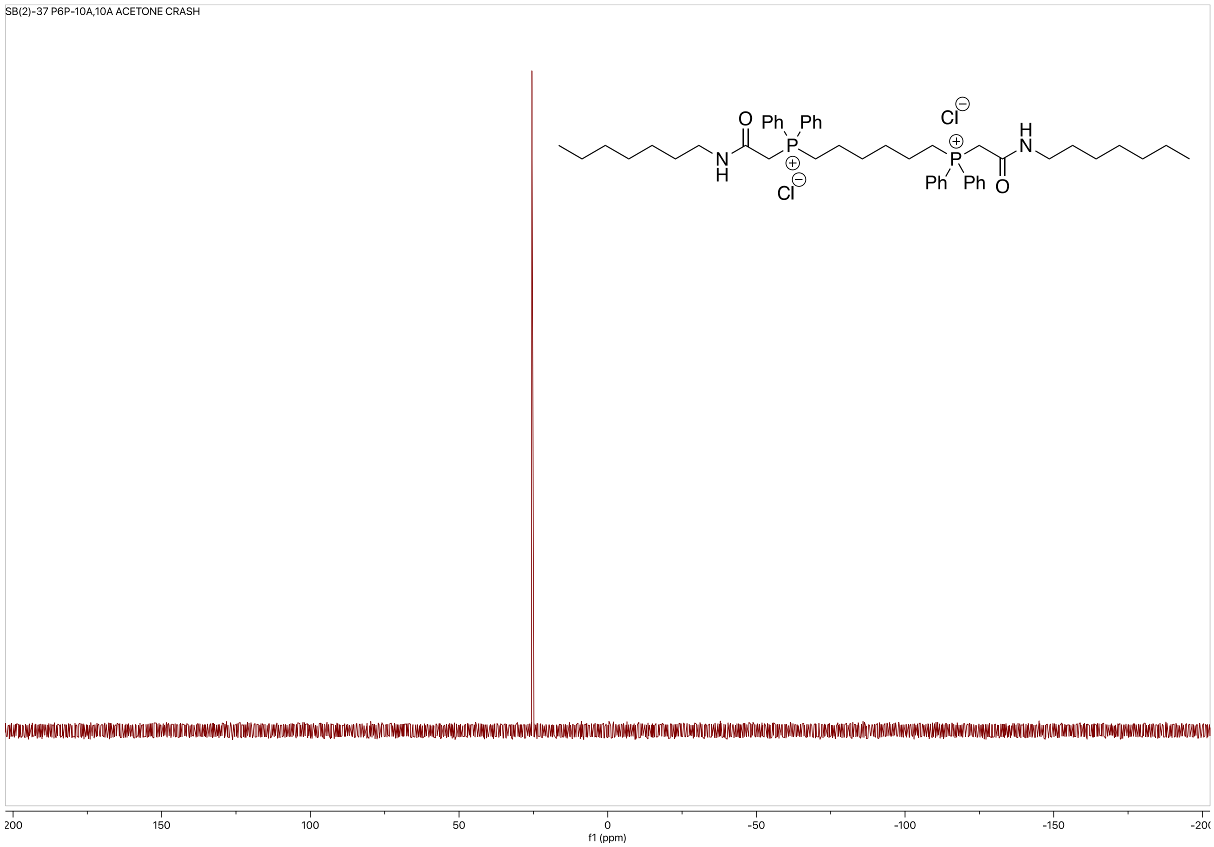

Figure S18c. ^{31}P NMR (162 MHz) of **P6P-10A,10A** in $CDCl_3$.

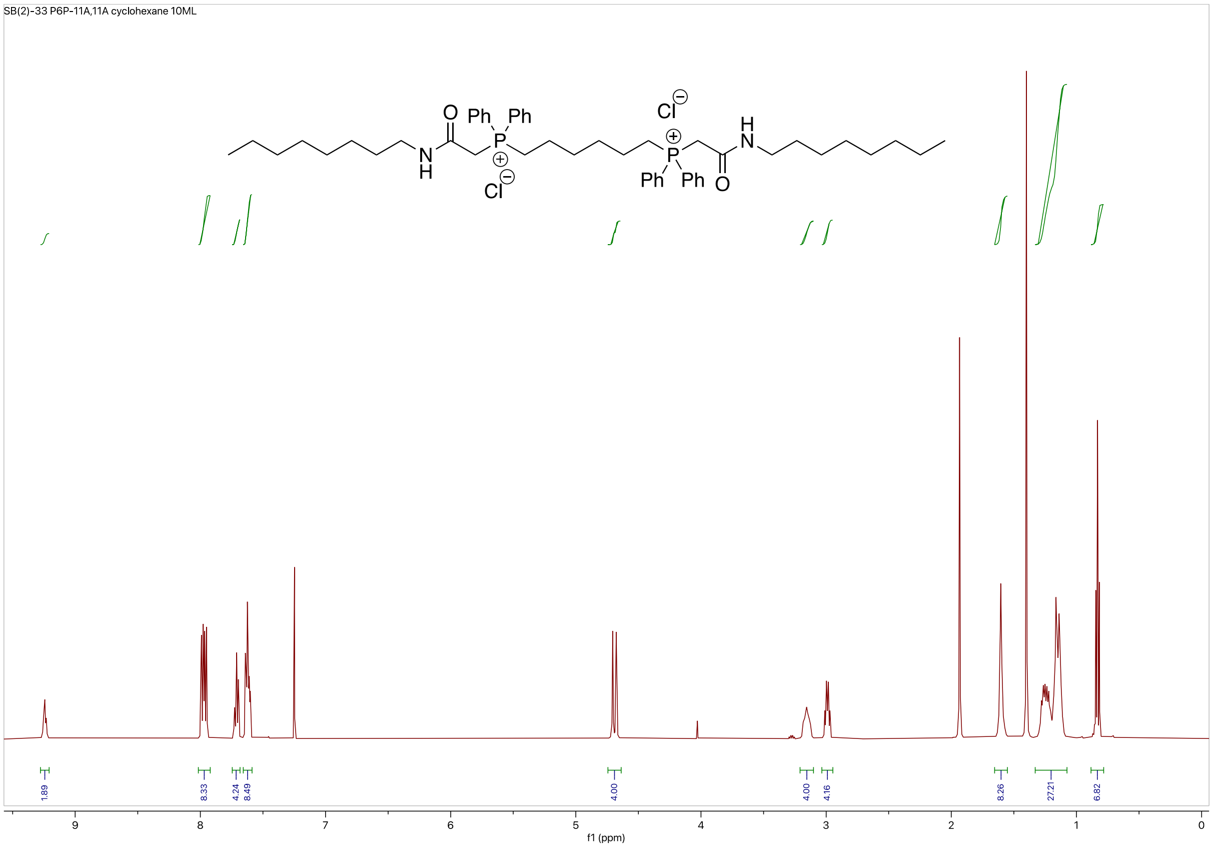

Figure S19a. ^{1}H NMR (400 MHz) of **P6P-11A,11A** in $CDCl_3$.

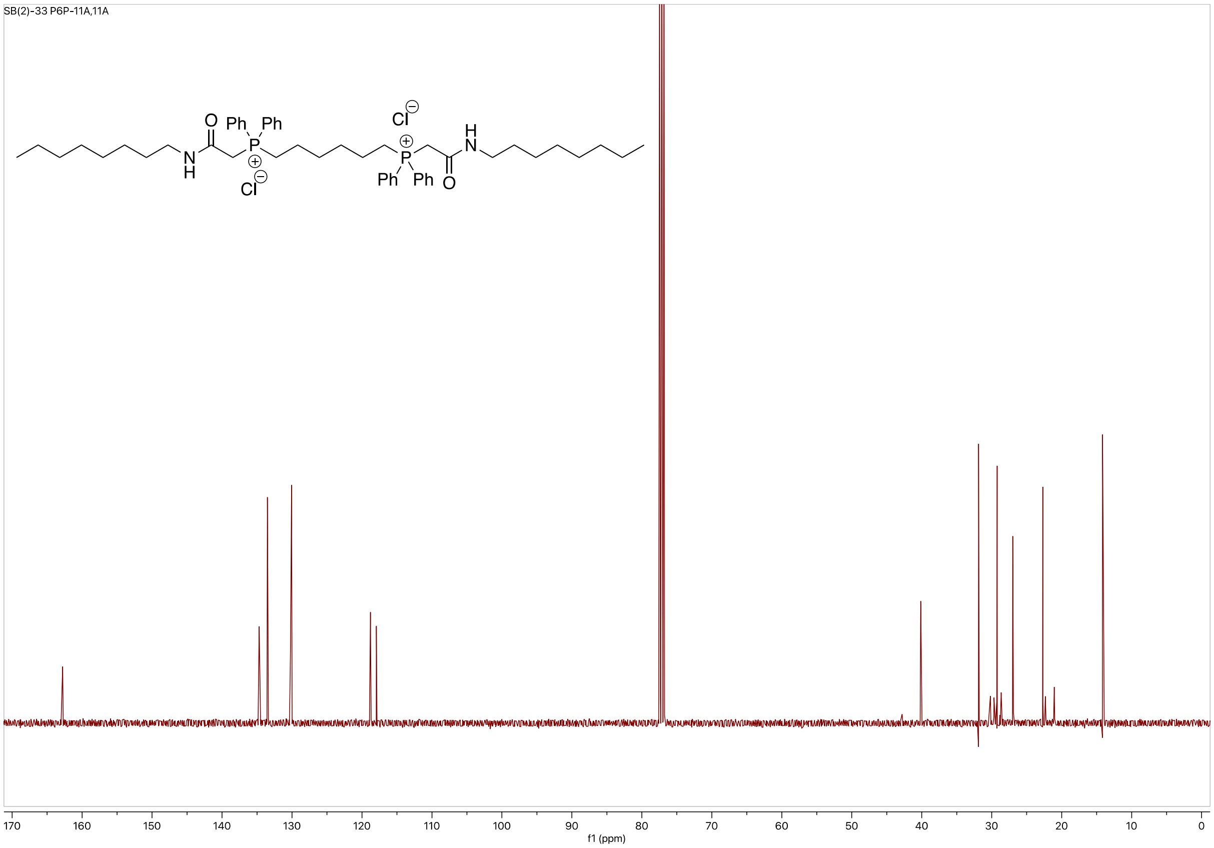

Figure S19b. ^{13}C NMR (100.6 MHz) of **P6P-11A,11A** in $CDCl_3$.

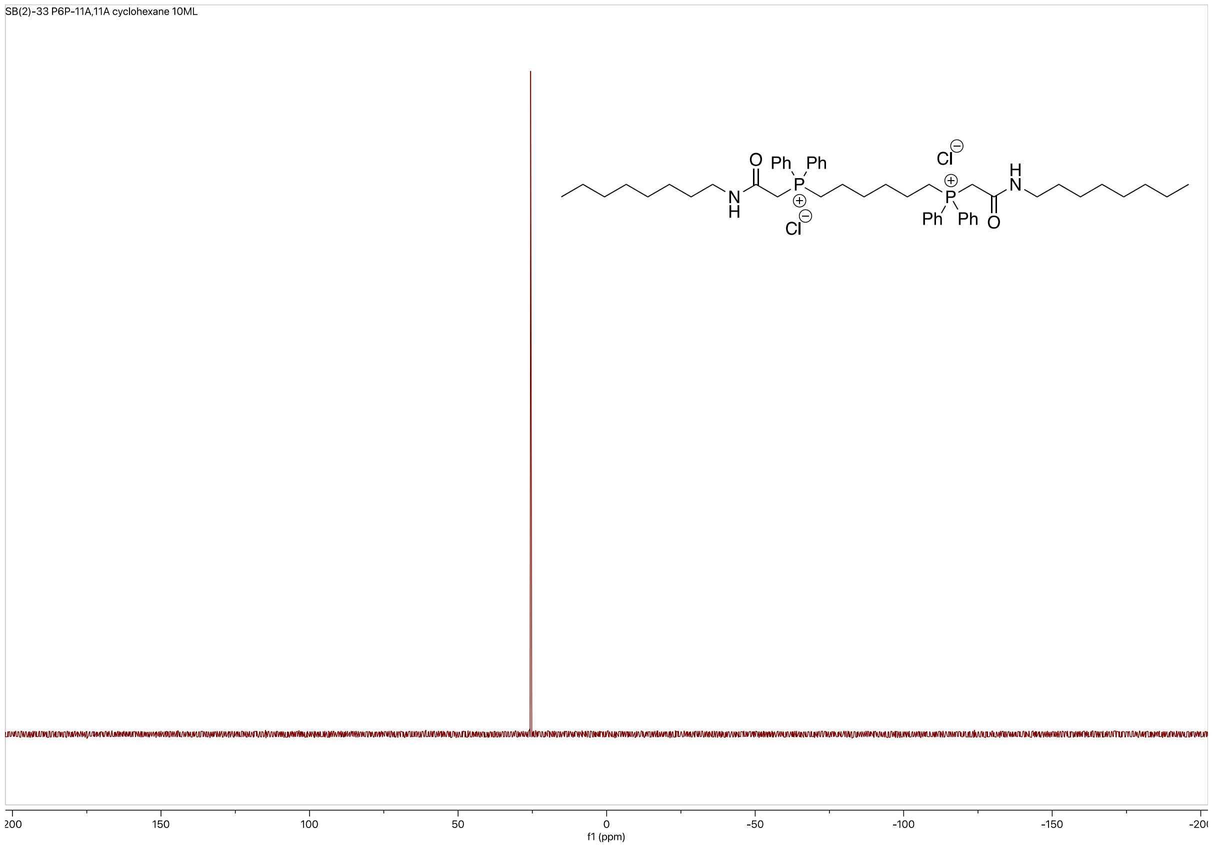

Figure S19c. ^{31}P NMR (162 MHz) of **P6P-11A,11A** in $CDCl_3$.

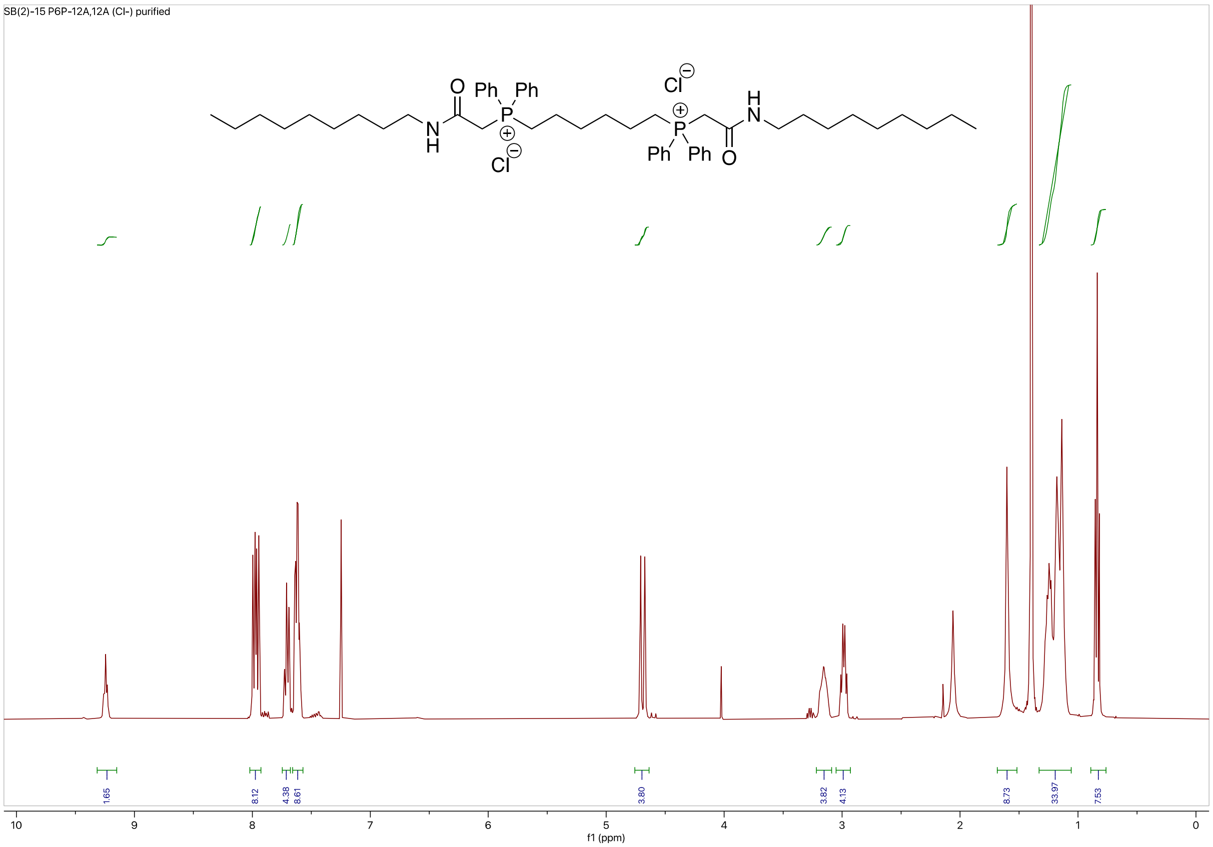

Figure S20a. ^{1}H NMR (400 MHz) of **P6P-12A,12A** in $CDCl_3$.

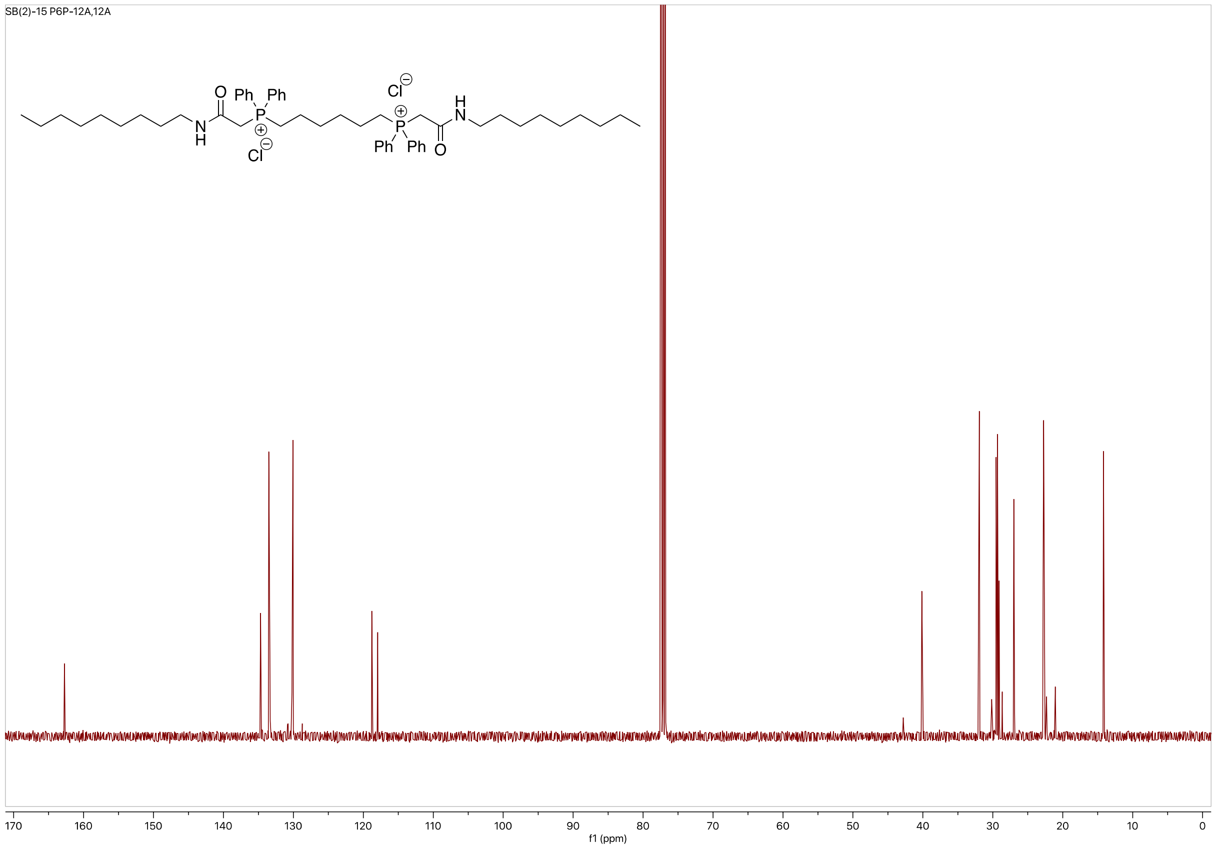

Figure S20b. ^{13}C NMR (100.6 MHz) of **P6P-12A,12A** in $CDCl_3$.

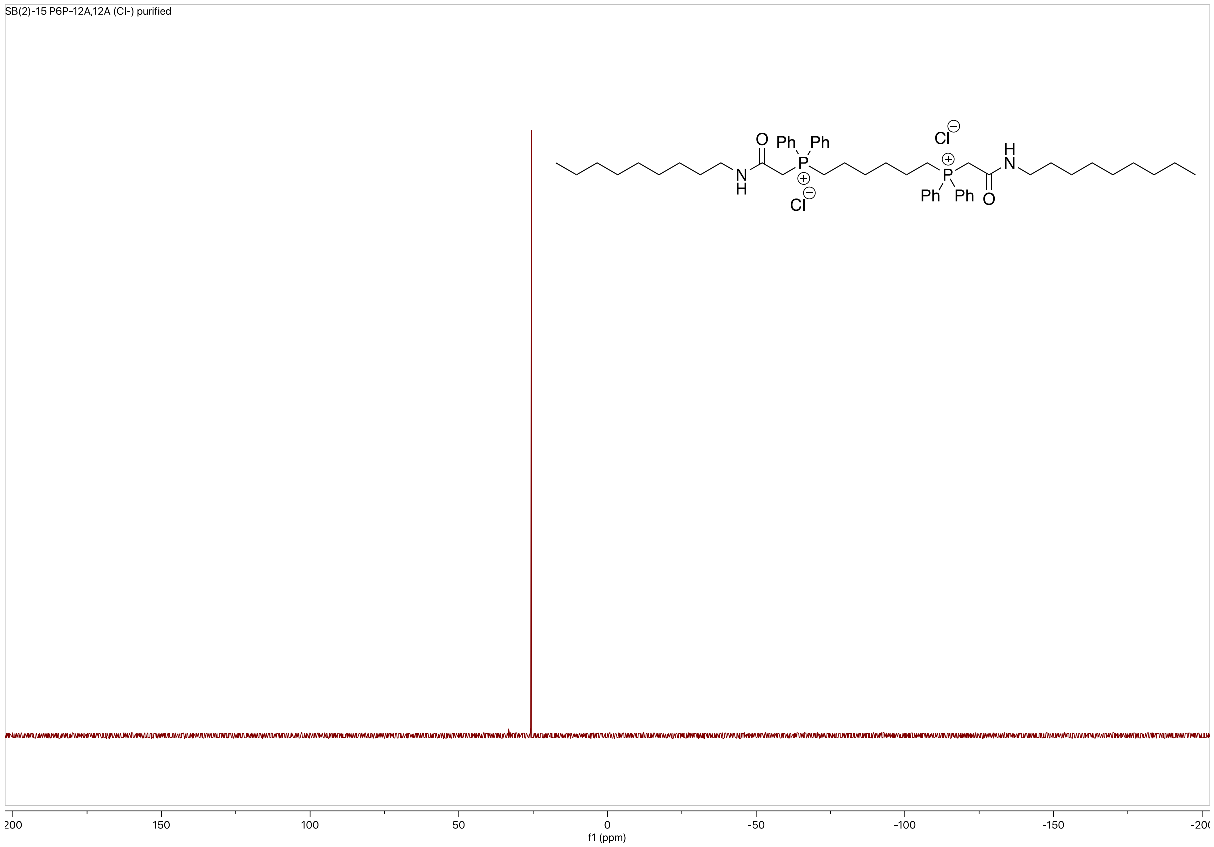

Figure S20c. ^{31}P NMR (162 MHz) of **P6P-12A,12A** in $CDCl_3$.

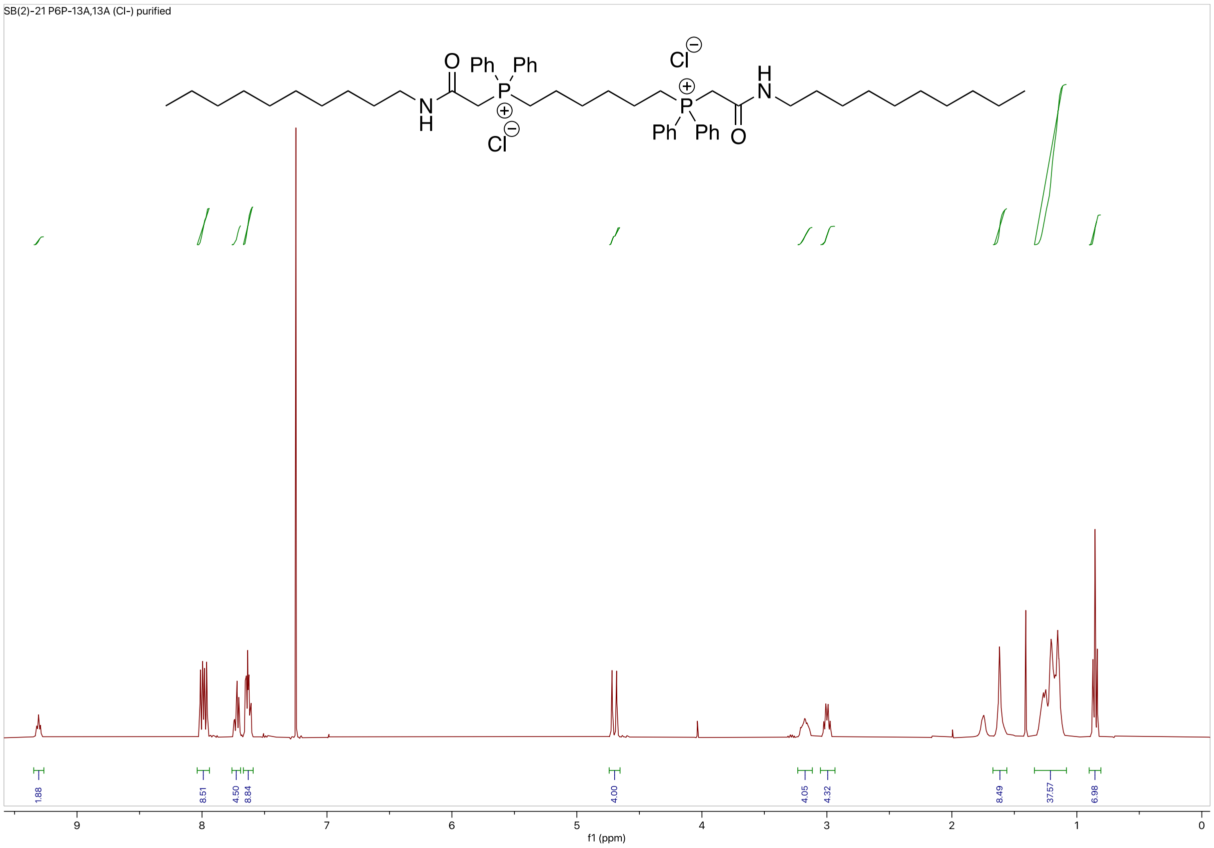

Figure S21a. ^{1}H NMR (400 MHz) of **P6P-13A,13A** in $CDCl_3$.

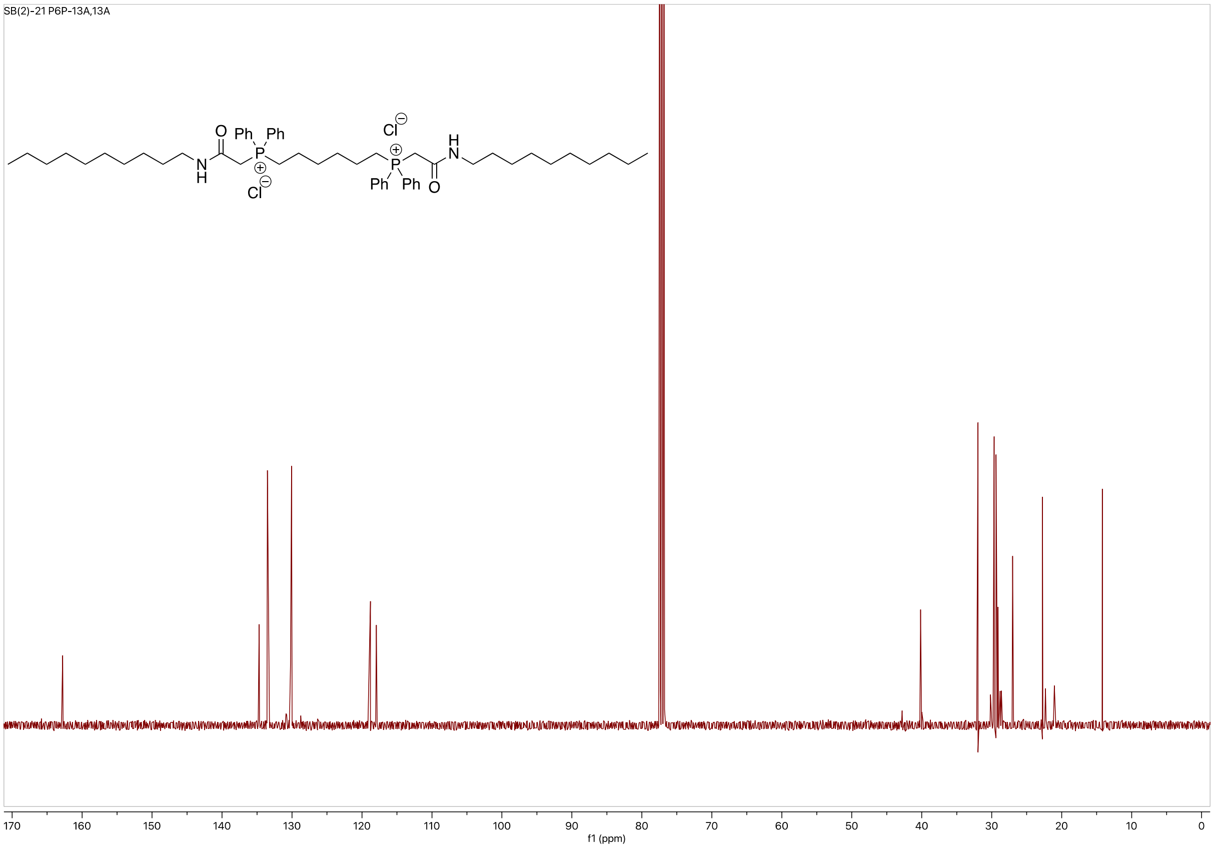

Figure S21b. ^{13}C NMR (100.6 MHz) of **P6P-13A,13A** in $CDCl_3$.

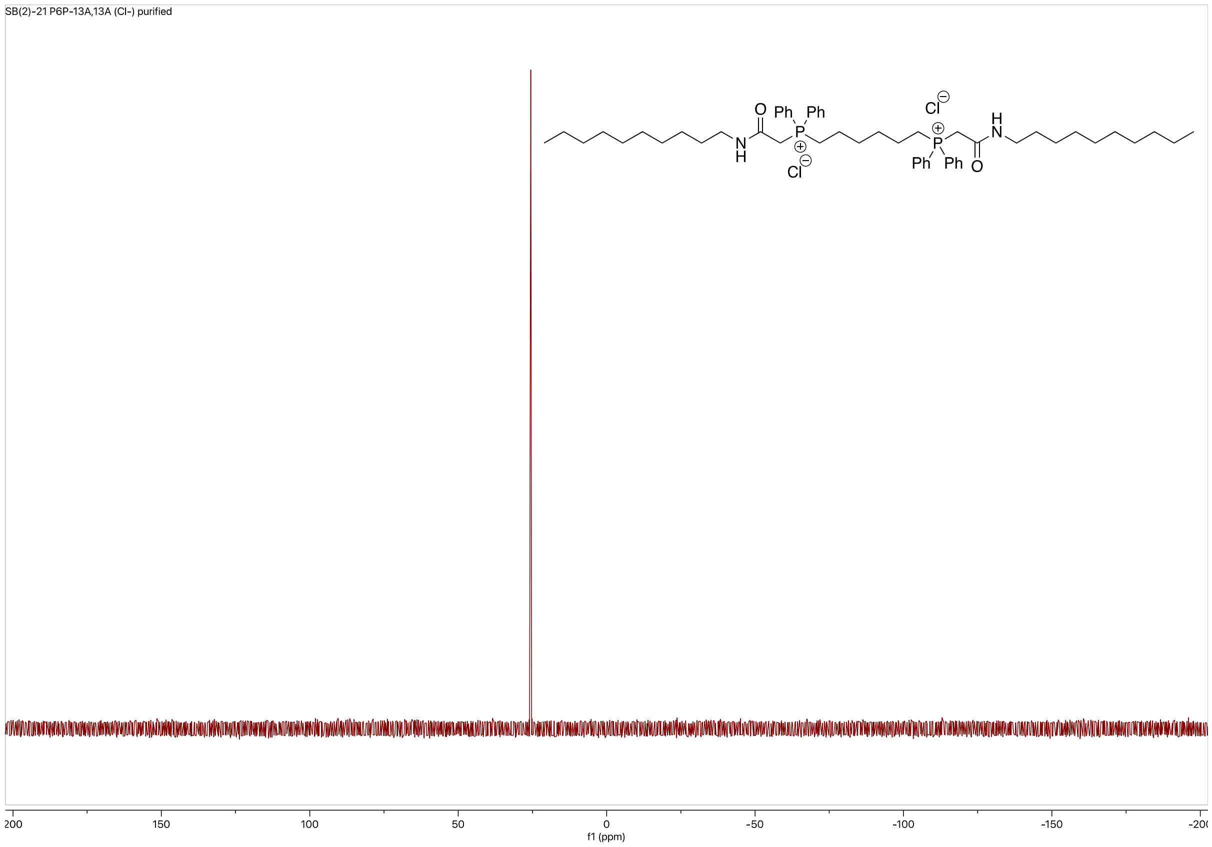

Figure S21c. ^{31}P NMR (162 MHz) of **P6P-13A,13A** in $CDCl_3$.

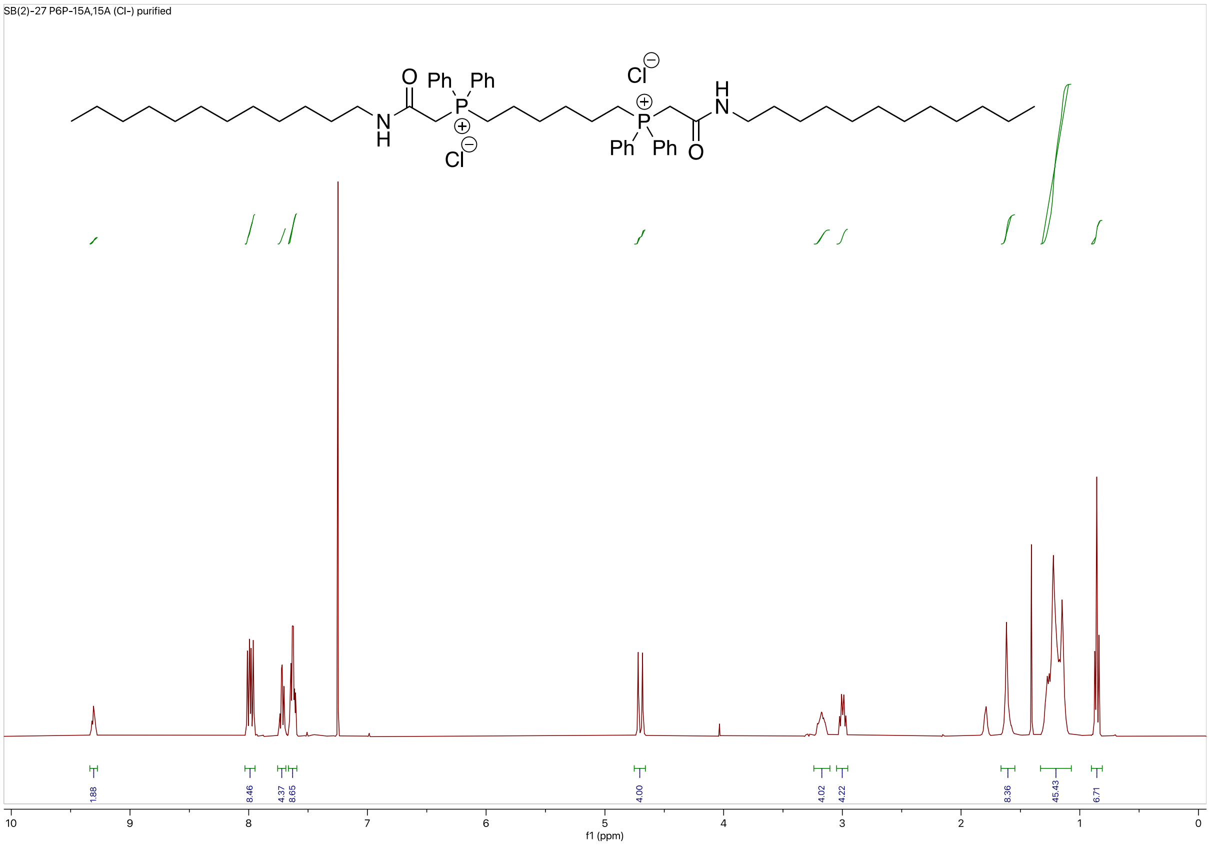

Figure S22a. ^{1}H NMR (400 MHz) of **P6P-15A,15A** in $CDCl_3$.

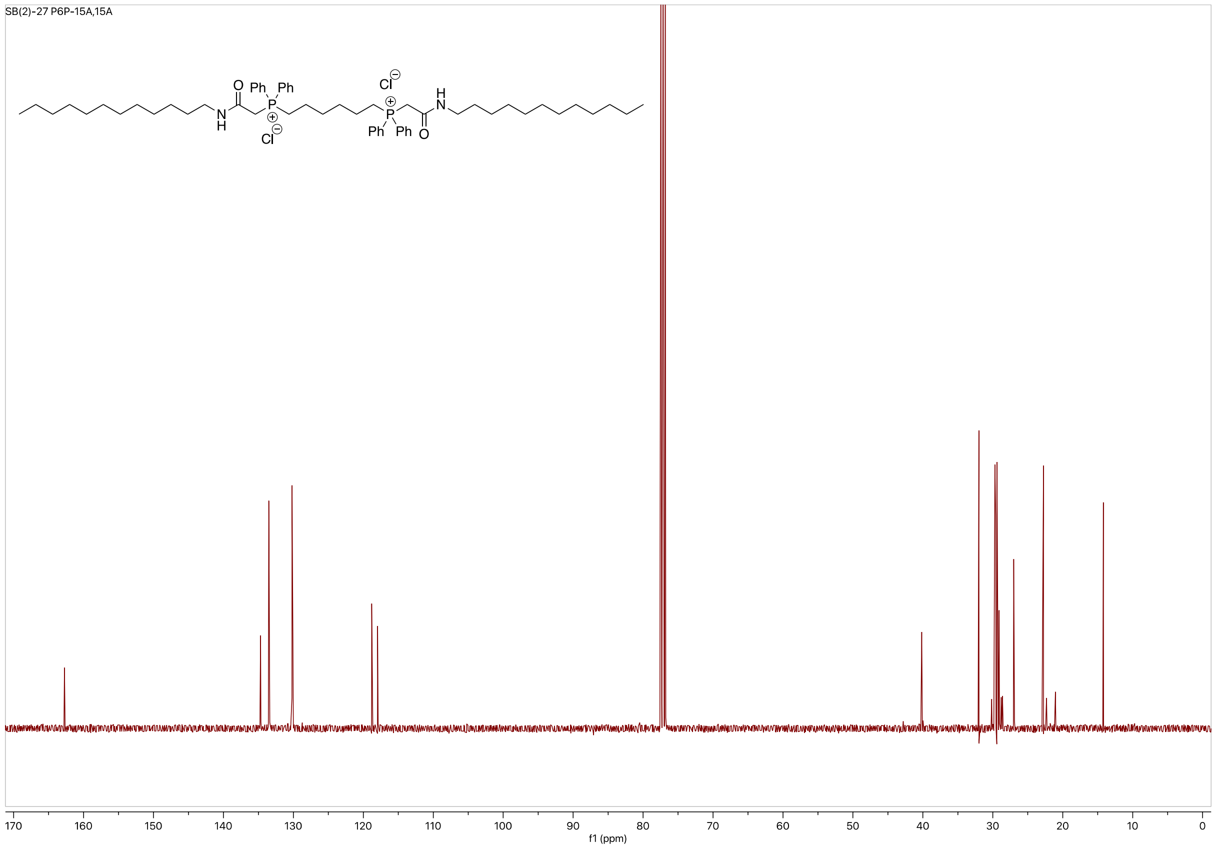

Figure S22b. ^{13}C NMR (100.6 MHz) of **P6P-15A,15A** in $CDCl_3$.

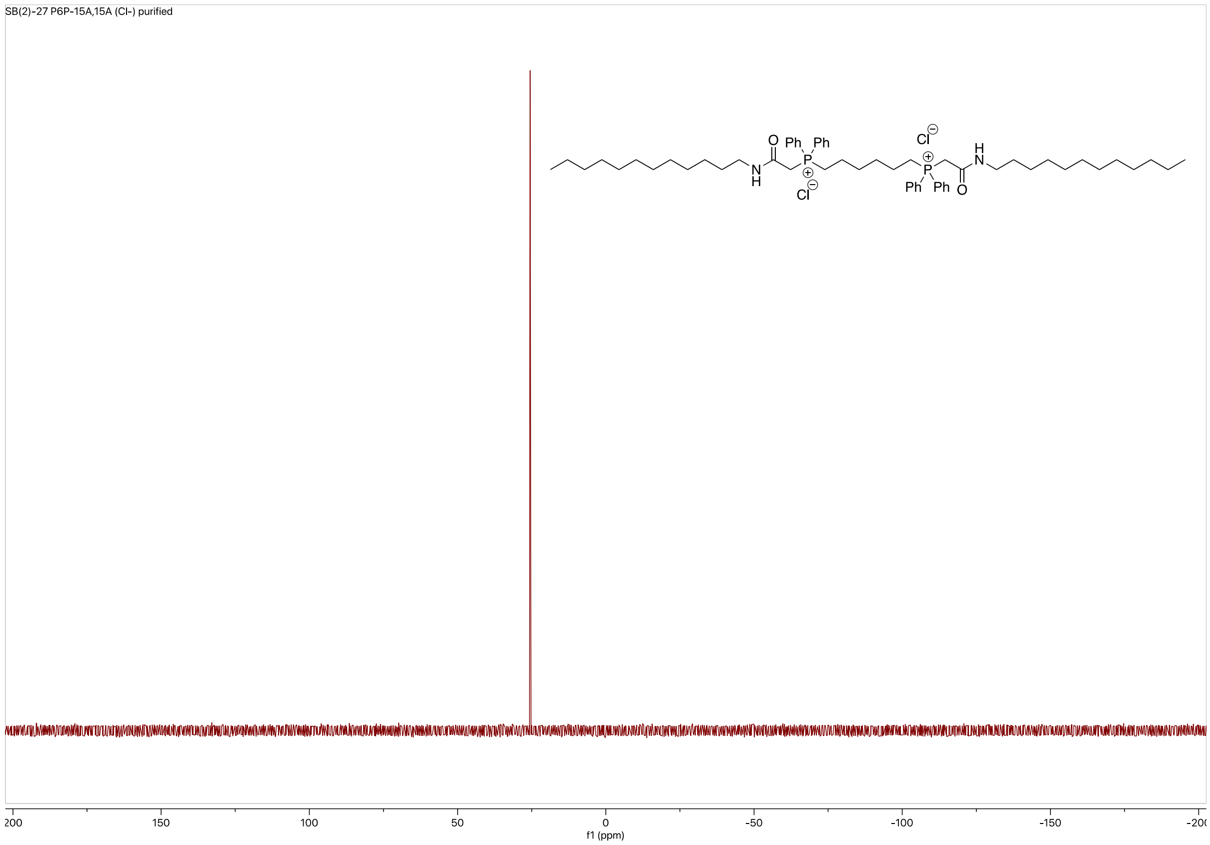

Figure S22c. ^{31}P NMR (162 MHz) of **P6P-15A,15A** in $CDCl_3$.

IV. Crystallographic Data

Table S1. Summary of structure determination for complexes P3P-8A,8A

	**P3P-8A,8A**
**Empirical formula**	$C_{42}H_{57.5}Cl_2N_{2.5}O_3P_2$
**Formula weight**	778.24 g/mol
**Diffractometer**	Rigaku XtaLAB Synergy-S (HyPix-6000HE)
**Temperature/K**	100
**Crystal system**	orthorhombic
**Space group**	Pnma
**a**	13.72662(9) Å
**b**	30.8902(2) Å
**c**	20.23413(18) Å
**Volume**	8579.64(12) Å^3
**Z**	8
**d_{calc}**	1.205 g/cm^3
**μ**	2.368 mm^{-1}
**F(000)**	3320.0
**Crystal size, mm**	0.58 × 0.02 × 0.01
**2θ range for data collection**	5.722 - 149.006°
**Index ranges**	$-16 \leq h \leq 17$, $-38 \leq k \leq 38$, $-25 \leq l \leq 23$
**Reflections collected**	138853
**Independent reflections**	8956 [R(int) = 0.1031]
**Data / restraints / parameters**	8956 / 43 / 519
**Goodness-of-fit on F^2**	1.020
**Final R indexes [I>=2σ (I)]**	$R_1 = 0.0518$, $wR_2 = 0.1426$
**Final R indexes [all data]**	$R_1 = 0.0603$, $wR_2 = 0.1492$
**Largest diff. peak / hole**	0.77 / -0.48 eÅ^{-3}
**CCDC**	2196105

V. Mitochondrial Toxicity Data

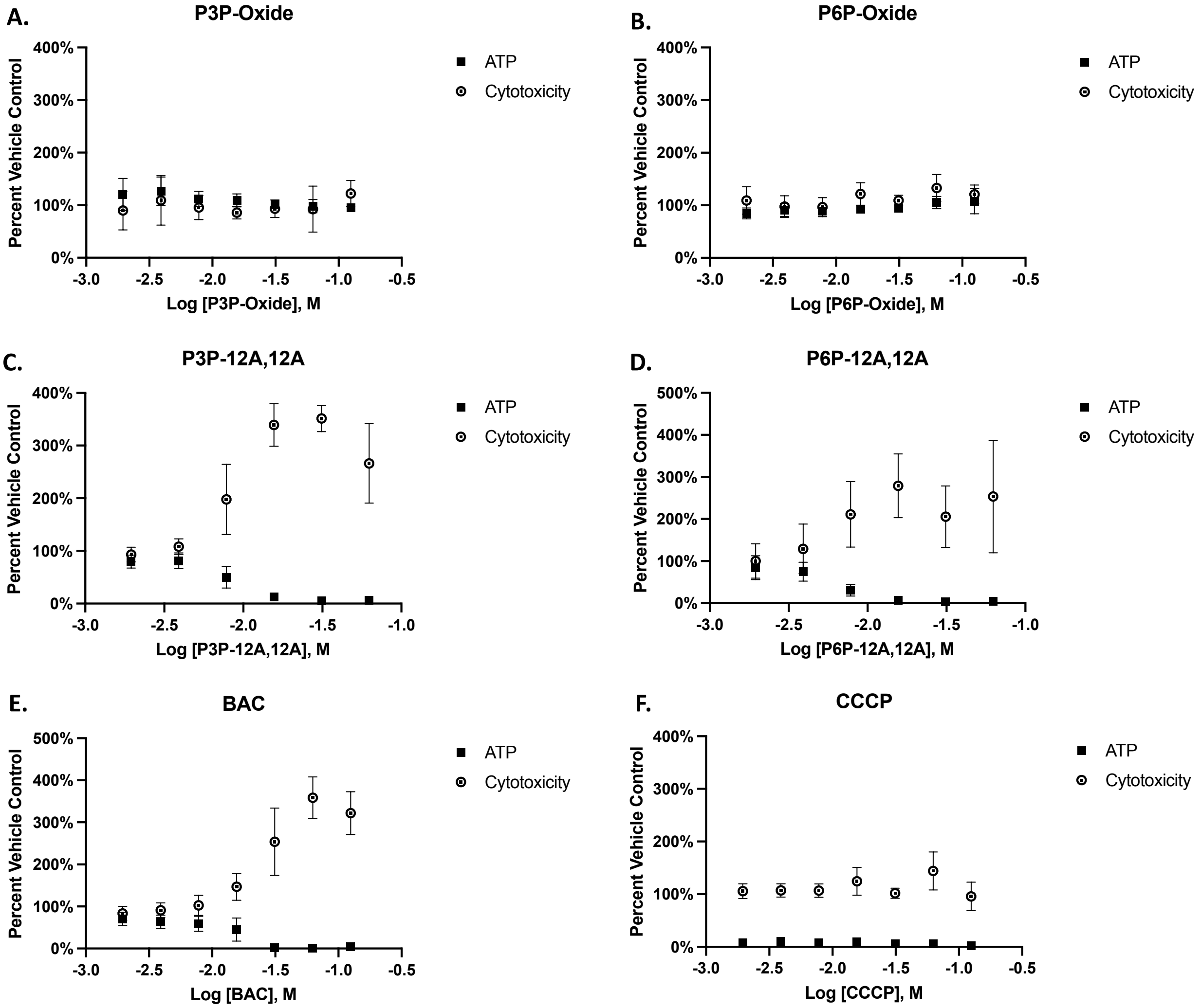

Figure S23. Mitochondrial toxicity of compounds with the Mitochondrial ToxGlo™ Assay. Panel A. and B. show **P3P-Oxide** and **P6P-Oxide**, respectively, with no changes in ATP or membrane integrity (MI). Panel C., D., and E. show **P3P-12A,12A**, **P6P-12A,12A**, and control benzalkonium chloride (**BAC**), respectively, with a reduction in ATP with commensurate MI changes indicating the occurrence of primary necrosis. Panel E. shows the protonophore **CCCP** reducing ATP concentration with no changes in MI, indicating mitochondrial toxicity.

Table S2.Raw Data for **Mitochondrial toxicity of compounds with the Mitochondrial ToxGlo™ Assay.**

	Percent Vehicle Control Cytotoxicity +/- SD %						
Conc. (∝M)	125	63	32	16	8	4	2
P3P-Oxide	122 ± 25%	110 ± 10%	93 ± 17%	86 ± 12%	96 ± 23%	109 ± 47 %	90 ± 37%
P6P-Oxide	121 ± 18%	133 ± 26%	109 ± 10%	122 ± 21 %	97 ± 18%	98 ± 21%	109 ± 26%
P3P-12A,12A	*	266 ± 75%	352 ± 25%	339 ± 41%	198 ± 67%	108 ± 15%	93 ± 14%
P6P-12A,12A	*	253 ± 134%	206 ± 73%	279 ± 76%	211 ± 78%	129 ± 59%	100 + 41%
BAC	322 ± 51%	359 ± 50%	254 ± 80%	147 ± 32%	103 ± 24%	91 ± 18%	84 ± 17%
CCCP	96 ± 27%	144 ± 36%	102 ± 10%	125 ± 26%	107 ± 13%	107 ± 13%	106 ± 14%

	Percent Vehicle Control ATP +/- SD %						
Conc. (∝M)	125	63	32	16	8	4	2
P3P-Oxide	95 ± 6%	98 ± 13%	100 ± 7%	109 ± 12%	112 ± 15%	126 ± 27%	120 ± 30%
P6P-Oxide	98 ± 4%	105 ± 12%	95 ± 7%	92 ± 5%	89 ± 7%	91 ± 13%	85 ± 10%
P3P-12A,12A	*	6 ± 3%	5 ± 2%	12 ± 5%	50 ± 20%	81 ± 15%	80 ± 13%
P6P-12A,12A	*	4 ± 2%	3 ± 2%	7 ± 4%	31 ± 14%	75 ± 22%	84 ± 29%
BAC	4 ± 2%	1 ± 1%	2 ± 1%	45 ± 27%	59 ± 18%	64 ± 16%	70 ± 16%
CCCP	3 ± 0.4%	5 ± 1%	5 ± 0.5%	9 ± 2%	8 ± 1%	10 ± 2%	8 ± 0.5%

* **P3P-12A,12A** and **P3P-12A,12A** interfere with the fluorescent probe at higher concentrations, therefore that data is not included.
